# The diversity of ignorance and the ignorance of diversity: origins and implications of “shadow diversity” for conservation biology and extinction

**DOI:** 10.1017/ext.2024.21

**Published:** 2024-11-22

**Authors:** Serena Turton-Hughes, George Holmes, Christopher Hassall

**Affiliations:** 1 School of Earth and Environment, Faculty of Environment, University of Leeds, Leeds, UK; 2 School of Biology, Faculty of Biological Sciences, University of Leeds, Leeds, UK

**Keywords:** unknown diversity, dark extinction, taxonomic bias, biodiversity shortfalls, scientific ignorance

## Abstract

Biodiversity shortfalls and taxonomic bias can lead to inaccurate assessment of conservation priorities. Previous literature has begun to explore practical reasons why some species are discovered sooner or are better researched than others. However, the deeper socio-cultural causes for undiscovered and neglected biodiversity, and the value of collectively analysing species at risk of unrecorded, or “dark”, extinction, are yet to be fully examined. Here, we argue that a new label (we propose “shadow diversity”) is needed to shift our perspective from biodiversity shortfalls to living, albeit unknown, species. We suggest this linguistic shift imparts intrinsic value to these species, beyond scientific gaze and cultural systems. We review research on undiscovered, undetected and hidden biodiversity in the fields of conservation biology, macroecology and genetics. Drawing on philosophy, geography, history and sociology, we demonstrate that a range of socio-cultural factors (funding, education and historical bias) combine with traditional, practical impediments to limit species discovery and detection. We propose using a spectrum of shadow diversity which enables a complex, non-binary and comprehensive approach to biodiversity unknowns. Shadow diversity holds exciting potential as a tool to increase awareness, appreciation and support for the conservation of traditionally less studied wildlife species and sites, from soil microbes to less charismatic habitat fragments. We advocate for a shift in how the conservation community and wider public see biodiversity and an increase in popular support for conserving a wider range of life forms. Most importantly, shadow diversity provides appropriate language and conceptual frameworks to discuss species absent from conservation assessment and at potential risk of dark extinction.

## Impact statement

Contemporary approaches to the study of biodiversity have emphasised the great complexity of natural systems in terms of the composition of biological communities and the many interactions within them. Practical conservation measures have reacted to these emerging themes, with the gradual introduction of new survey methods and new conservation approaches. In this review, we describe the ways in which our knowledge of biodiversity has arisen and the consequences of the gaps in our understanding of the living world. We argue that there is a need for a proactive approach to the conservation of nature that incorporates uncertainty and the unknown. We explore dark extinctions (those species that go extinct before being discovered) and unknown biodiversities (those species that are still unknown to science), offering “shadow diversity” as a collective term to broaden perspectives of what can be included in conservation considerations. The article provides a guide for future multidisciplinary considerations of causes of shadow diversity and taxonomic biases in conservation science and ecology. We conclude with a series of four challenges presented by shadow diversity with suggestions for directions of future research: (i) problems of detection, (ii) problems of language, (iii) problems of valuation and (iv) problems of practical application. Such findings should be of interest to academics who are examining interdisciplinary responses to conservation and extinction, as well as conservationists who are developing frameworks for the sustainable conservation of natural resources.

## Introduction

The contemporary phase of anthropogenic biodiversity decline has been described as a “mass extinction” on the basis of the rates of species loss (Barnosky et al. 2011). However, a sizeable but uncertain fraction of that loss is likely to comprise “dark extinctions” of species already extinct but never known to science (Tedesco et al. 2014; Chisholm et al. 2016; Lambdon and Cronk 2020). Despite our increasing ability to detect and monitor the natural world, there will almost certainly be further dark extinctions in the future (Pimm et al. 2014). Understanding and preventing dark extinctions is important for accurate historical records of environmental destruction (Boehm and Cronk 2021) and for uncovering hidden or cryptic species for conservation purposes (Milić et al. 2019). Beyond the practicalities of environmental protection, we also note the moral and emotional imperatives for seeing, mourning and processing the loss of unknown life (see, for instance, Head 2016; Jørgensen 2019). Here, we develop a complementary concept to dark extinction that we term “shadow diversity”. This concept is derived from an exploration of the significance of extant, unknown species but emphasises the presence of unknown taxa as opposed to their absence. Contemporary conservation science is aware of the need to incorporate uncertainty and unknowns, evidenced by numerous statistical tools and models for this purpose (for instance, see Chao et al. 2017; MacKenzie et al. 2017; Benoit et al. 2018). Here, we complement quantitative approaches, by providing a multidisciplinary analysis focussed on revealing processes beyond conservation that generate and maintain biodiversity’s unknowns. We offer a conceptual framework that can help researchers, policymakers and the general public to articulate those unknowns, explore their causes and mitigate their consequences to produce more taxonomically holistic conservation practices, systems and solutions.

This review extends past an acknowledgement of the existence of living and extinct unknown and unrecorded biodiversity, to examine social, cultural, political factors and psychological biases that contribute to ignorance that is unevenly distributed across the phylogenetic Tree of Life and different habitats. We begin by examining unknown extinctions and biodiversity in the context of conservation science. Next, we review the literature on unknown extinctions and unknown biodiversity. We argue for the concept of “shadow diversity” for two key purposes: firstly, as a useful tool to enable the discussion of otherwise nebulous absent lifeforms; and, secondly, as a framework to explore deeper multidisciplinary origins of taxonomic biases of unknown species that might inform future philosophical and scientific thinking. We examine literature dealing with reasons for this imbalance of knowledge across the Tree of Life and provide a model to deepen cross-disciplinary explorations of causes of ignorance and how to overcome this. Finally, we provide four challenges posed by shadow diversity and suggest how shadow diversity can impact conservation. By demonstrating the socio-cultural origins of our (conservation scientists and beyond) ignorance, we propose multi- and interdisciplinary approaches to understanding, managing and building societal relations with biodiversity, including biodiversity at risk of extinction that cannot be named but can be noticed.

## Unknown extinctions and unknown biodiversity in conservation science

Conservation biology is a crisis discipline (Soulé 1985), tackling the extinction crisis (Barnosky et al. 2011). To accomplish this aim, conservation biology answers questions about what life forms are in danger of extinction and how they can be saved, monitoring biodiversity at local and global levels using species population sizes, trends and extinction rates. Conservation scientists work with international bodies, such as the IUCN (International Union for Conservation of Nature), to provide data that informs the conservation status of assessed species. The IUCN conservation status of a species informs policies and legal protections for that species (Rodrigues et al. 2006). Since high proportions of biodiversity are thought to be unknown (Mora et al. 2011), experts calculate estimates for where these undiscovered species might be (Moura and Jetz 2021). The IPBES (Intergovernmental Science-Policy Platform on Biodiversity and Ecosystem Services) provides detailed and, whilst imperfect, well-founded quantitative estimates for biodiversity trends (IPBES 2019). Despite the robustness of the data, the interpretation of those trends has proven controversial in some areas of society so it was necessary to add a question-and-answer page explaining and defending the IPBES Global Biodiversity 2019 report (Purvis 2019b). That Purvis’s response was needed echoes Morton’s comparison that providing statistical data for government and public interpretation leaves it open to individuals’ “mental construct” of cause and effect (Morton 2016, 14). In spite of a large team of experts choosing “conservative” estimates for the IPBES (Purvis 2019a), the IPBES report continues to provoke extinction denial (Lees et al. 2020).

There is a growing body of literature in conservation that recognises the importance of what we have termed shadow diversity. The need to investigate unknown species for the purpose of improving conservation assessment accuracy has been thoroughly established (Bickford et al. 2007; Fišer et al. 2018; Cornwell et al. 2019). Scientists have repeatedly attempted to quantify the number of undiscovered species: one paper even thanked colleagues “supporting our decision not to provide yet another conjectural estimate of the total number of species on Earth” (Scheffers et al. 2012). Such estimates cover many taxa, from microalgae (Jouannais and Pizzol 2022) and ants (Kass et al. 2022), soil biodiversity (Anthony et al 2023), through to full global figures (Mora et al. 2011; IPBES 2019). However, we do not have calculations for every taxon (Purvis 2019b), such as fresh-water nematodes (Anthony et al 2023). Estimates that we have vary widely as researchers compensate in different ways for incomplete data, such as extinction data beyond IUCN red lists (Régnier et al 2015). The accuracy of and assumptions within these estimates means the number of undiscovered species remains a contested area. Scheffers et al. (2012) are right to urge caution towards global estimates, especially where small-scale local data is used to extrapolate large-scale estimates, due to the questionable estimates that this leads to. In the absence of precise numerical accuracy, we can say that there seems more than sufficient evidence of a current biodiversity crisis and within this, what we term shadow diversity is a global challenge. What these figures do not inform us of is how these species knowledge gaps occurred.

In addition to debates over the interpretation of existing data, undiscovered species for which there are no data may experience a higher likelihood of extinction (Liu et al. 2022). As Purvis (2019b) writes “Ignoring them would therefore be not only silly but decidedly unscientific. The question is, how best to consider them?” We ask the same question. In our review, we steer away from considering undiscovered species in a purely quantitative sense. Instead, we develop a different approach that examines the underlying causes of our lack of knowledge about certain taxa and involves an expansion of interdisciplinary enquiry into the practice of conservation. By working alongside longstanding quantitative estimates of biodiversity trends and extinctions, we hope to provide a multidisciplinary, qualitative framework for understanding the causes and consequences of the loss of those taxa that lie beyond the reach of conventional statistics. In developing this shadow diversity framework, we review literature specific to unknown extinctions and undiscovered biodiversity from different disciplinary angles. The results are not intended as a solution to any individual component of the extinction crisis, but rather as an alternative perspective that may be useful in evaluating the systemic drivers and consequences of the existence of poorly recorded, threatened taxa, grounded in a deeper understanding of where unknowns come from.

## A review of unknowns

It has long been recognised that human-induced extinctions are occurring before species are formally described. For example, Ainsworth (1976, 296) predicted numerous species of fungi and insects would be extinct before their scientific discovery. High rates of biodiversity loss present a race against time to find species before they are lost (Costello et al. 2013). Initially, specific species, particularly birds and snails, were posthumously recorded as extinct based on zooarchaeological techniques or physical remains (Olson and James 1982; Pimm et al. 1994; Steadman 1995; Bouchet and Abdou 2003; Richling and Bouchet 2013; Cooke et al. 2023). More recently, statistical models have been employed to estimate the quantities of undiscovered species extinctions (Tedesco et al. 2014; Chisholm et al. 2016; Kristensen et al. 2020; Lum et al. 2021). To comprehend how future unrecorded extinctions might occur and how this could be minimised, it is also essential to understand trends in research on extant unknown biodiversity.

Concerns around ignorance of what species are still unknown, where they are located, what conservation solutions are needed to conserve them, and the implications that might follow their extinction are well-known to conservation biologists as biodiversity shortfalls (Lomolino 2004; Bini et al. 2006; Cardoso et al. 2011; Diniz-Filho et al. 2013; Hortal et al. 2015). Though we note proposals for additional shortfalls (for example: Cornwell et al. 2019; Martin et al. 2023), we summarise the seven main shortfalls in Figure 2. These shortfalls highlight the types of knowledge lacking to enable improved conservation information useful in preventing and managing anticipated future extinctions. In order to review the lacking knowledge itself, rather than the types of knowledge lacking, we explored studies focused on unknown biodiversity, both extant and extinct, as we describe below.

### Methods

**Figure 1.**
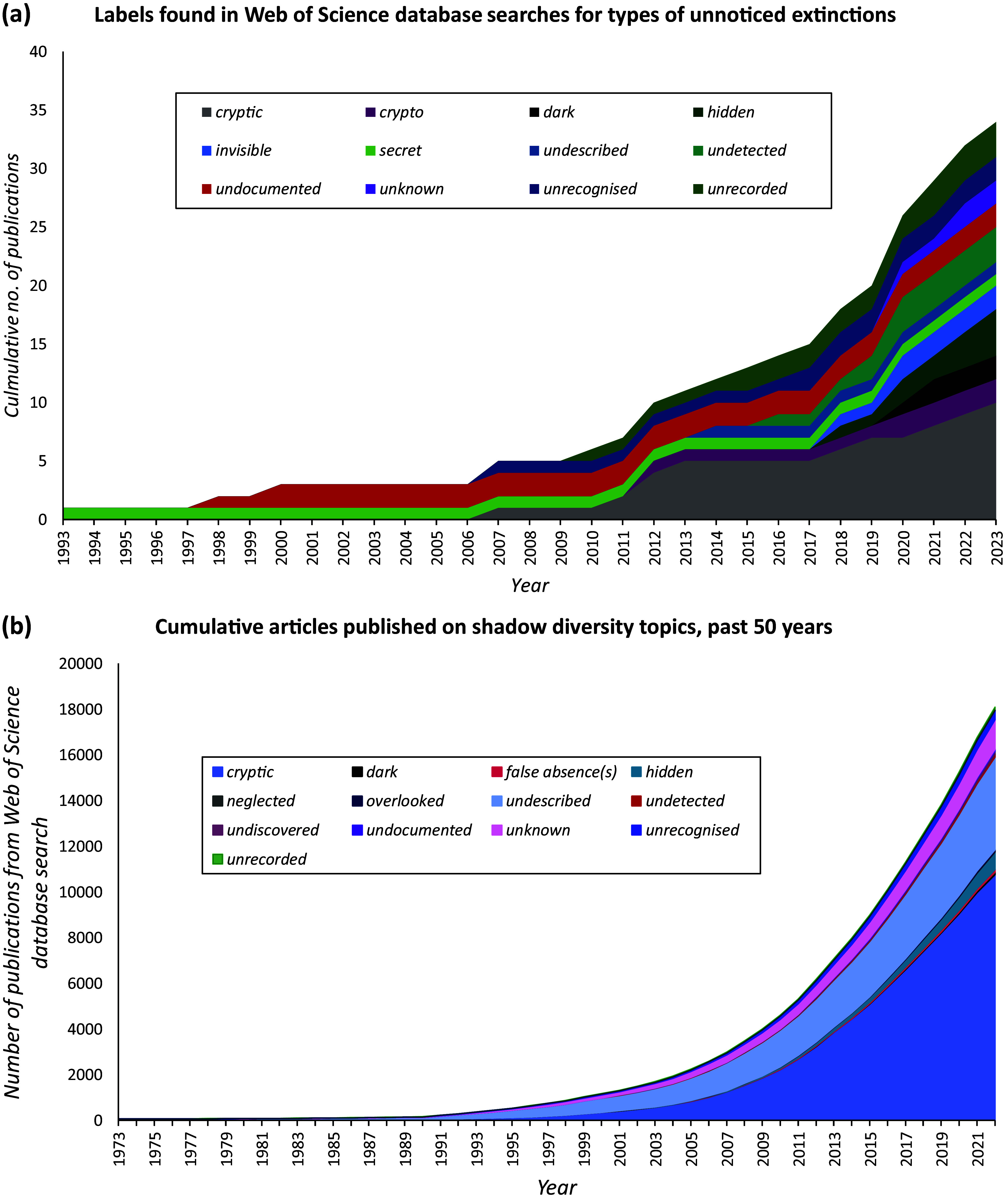
(a) A stacked area graph showing the number of publications on unknown extinction topics over time. The graph starts at the year 1993 with one publication on ‘secret extinction’ and tracks a further eleven terms published over time to the present day (2023). (b) A stacked area chart showing 13 labels found in literature relating to shadow diversity language and the cumulative total number of publications found in the Web of Science database for each term under a ‘topic’ search, plotted over time for the past 50 years. The shape of the graph becomes increasingly exponential towards the right-hand side.

We examined twelve terms used to describe unknown species extinctions. This provided 34 relevant results, which we analysed in full (see Supplementary Table S2 for details including search terms, exclusions and a full reference list). Additionally, we undertook a second review using thirteen terms hitherto used to describe unrecorded species, taxa and biodiversities. We searched the Web of Science database using a “topic” search for publications up to 2023, with exclusions for irrelevant subject areas, such as astrophysics. We used the search terms “unknown taxa” OR “unknown taxon”, “unknown species” and “unknown biodiversity” OR “unknown diversity” for each term, including American-English spelling alternatives where appropriate. We utilised a mixed-methods multi-level approach to the literature (Grant and Booth 2009). Within this, we explored quantitative trends (Figure 1b, Supplementary Figure S6), as well as a critical narrative analysis of pre-existing reviews, and a systematised approach to analyse literature for each of the thirteen terms, reviewing 188 papers in depth (Supplementary Table S3). As the number of results was high (see Supplementary Figure S6), we noted, but excluded from quantitative analysis, a further four synonyms with fewer than ten publications (Supplementary Table S3). The range of terms included hidden diversity, cryptic taxa, false absences and unknown diversity (see Figure 1 for a full list).

### Overview of results

According to our review, “cryptic extinction” was the most frequent term used in research regarding unknown extinctions. However, there was insufficient data to suggest it as a dominant term for unknown extinctions as the number of publications per term in this search was small (Figure 1a). The most used prefix for unknown biodiversity was “cryptic” (cryptic taxa, species and diversity), followed by “undescribed” and then “unknown” (Figure 1b, see also Supplementary Figure S6). Some results from this second search employed the term in the context of highlighting a species was a new discovery (and therefore had *previously* been cryptic or unknown) see Supplementary Table S3). Even with allowances considered for this limitation, to date, our results suggest there has been more research effort on unknown extant species in conservation sciences than anthropogenic unknown extinctions. The main trends apparent from our review were mixed patterns in approaches for unknown extinctions and a split macro-, microscale trend for unknown biodiversity; use of deficit language that emphasises gaps without recognising the anthropocentric causes of those gaps and failure to engage with what might be missing; and imprecise use of terms that complicate the creation of a coherent picture on neglected taxa. These results led us to conclude that a new term would provide a useful tool to support consideration of unnamed biodiversity, both within and beyond conservation.

### Patterns in approaches

The relatively small number of articles on unknown extinctions did not show many overarching patterns with certainty. Our review found that work on unknown extinction varies across disciplinary approaches and scales (Supplementary Table S2). Most work to date estimates lost species within a taxonomic group and there was a notable pattern in research towards using statistical approaches. Whilst Tedesco et al. (2014) tested their model on regional and global datasets, most statistical approaches worked at the mesoscale with islands as a key theme (unknown bird, plant and butterfly extinctions in Singapore and St Helena (Chisholm et al. 2016; Kristensen et al. 2020; Lambdon and Cronk 2020; Theng et al. 2020)). Exceptions to a species-based focus included loss of genetic biodiversity (Ledig 1993; Ayoub and Hayashi 2008) and habitat loss (Bastian 2020). By comparison, literature that examines specific elements of unknown diversity (see Supplementary Table S3) has broadly focused on one of two main approaches. Firstly, macro-scale quantitative estimates can be calculated using probability and extrapolation from current data to map where we are most likely to find hitherto undiscovered biodiversity. Secondly, recent studies have used microscale, very often genetic, approaches to describe and classify cryptic diversity.

Micro approaches focus on uncovering species new to Western scientific knowledge, with the largest proportion of literature focused on cryptic species (Figure 1b and Supplementary Figure S6) and on the discussion of novel methods used to find them. Some authors discuss microscale studies within the wider context of conservation biology (Sales et al. 2018; Milić et al. 2019; Kortmann et al. 2022), but a considerable proportion do not make explicit links to applied conservation and focus, instead, on the fundamental discovery of new taxa only (Grabowski et al. 2017; Morinière et al. 2019; Sharkey et al. 2021).

Numerous models attempt to quantify ignorance, both for imperfect ecological and conservation data collection (Chao et al. 2017; MacKenzie et al. 2017; Benoit et al. 2018); and particular taxonomic groups. These large, often macro-, quantified estimates frequently take the form of proportions of species discovered compared to species yet unknown (Joppa et al. 2011; Hawksworth and Lücking 2017; Stork 2018) and mapping at a global scale where the remaining undiscovered species are predicted to be (Moura and Jetz 2021; Gatti et al. 2022). Here, there is a repeated theme of using quantitative modelling to estimate past and anticipated extinctions and imagine scales of what might have been and what might yet be lost. Undiscovered biodiversity estimates provide practical implications by highlighting geographical areas that might benefit most from international conservation funding or taxonomic support (Moura and Jetz 2021).

On the other hand, models for both dark extinctions and undetected extant biodiversity can struggle to perform well with lesser-known taxa and rare taxa, the two categories most in need of conservation (Giam et al. 2011; Roberts et al. 2016; Liu et al. 2022). Chao and colleagues note that their model for calculating undetected diversity will only perform well for difficult-to-detect species where there is a similar chance of detecting those species during sampling (similar detection probability) (Chao et al. 2017, 2927). This makes it appropriate for estimating undiscovered tree species, for example (Gatti et al. 2022). However, their model does not work well for species with variable detection probabilities, such as microbes (Chao et al. 2017, 2927), though it has been argued that these should be a key focus for anticipated dark extinction conservation (Veresoglou et al. 2015; Guerra et al. 2021; Redford 2023). Occupancy models and false absences tend to focus on those species that conservationists already know and seek, rather than undiscovered species (Smith et al. 1996; MacKenzie et al. 2003). Rare species are known to be methodologically challenging in these models (Jeliazkov et al. 2022). In practice, the inadequacy of models to handle rarity means extremely rare species are sometimes removed from detection rate calculations altogether (Driscoll 2010). Each of the three models used to estimate dark extinctions is suited to examining species where we already have good records. For example, Tedesco’s (2014 p. 1363) tool requires data from taxa whose discovery curve has already levelled. This is not practical for huge portions of the Tree of Life, such as the kingdom of fungi, where the species discovery curve is still rising (Hawksworth and Lücking 2017, 4).

### Lack of clarity

Of the terms we reviewed, some have been used since the mid-twentieth century and earlier (Figure 1), yet the boundaries and definitions of these labels remain unclear (Supplementary Table S3). For example, “cryptic species” has been used in the past to refer to species that are hard to detect due to their physical location or appearance (Claridge et al. 2004), but more recent use has been related to the taxonomic splitting of morphologically identical species using genetic sequencing (Supplementary Table S3). Hidden diversity for plants can mean locally rare or dormant (Carrasco-Puga et al. 2021) as well as “overlooked by traditional sampling” (Pärtel 2014). Hidden diversity, other than in plants, sometimes refers to species that can be uncovered using genetic sequencing techniques (Sales et al. 2018), thus overlapping with one use of cryptic diversity. Hidden diversity is at times used as a combined term for unknown diversity and suspected cryptic diversity, or both within the same article (Milić et al. 2019).

Further inconsistent uses occur where a term is only used in the title or abstract of an article and not defined (Shipunov et al. 2008; Grabowski et al. 2017). Undefined terms cause ambiguity and the reader is left to infer or interpret the type of unknown biodiversity under discussion, leading to a multitude of understandings from these terms. This variety of meanings, many of which are employed interchangeably across different labels, make it difficult to compare within and across the body of research literature regarding currently unknown taxa, likely to be at high risk of extinction (Giam et al. 2011). Exacerbating problems of ambiguity, discussions of terms and concepts generally examine one element in isolation: Several reviews examine cryptic species only (Knowlton 1993; Bickford et al. 2007; Fišer et al. 2018; Chenuil et al. 2019). Other studies review one kingdom only, such as the communities of fungi hidden within plants and insects (Blackwell and Vega 2018). With such overlaps of definitions, a shared umbrella term that allows for a spectrum of unknowns would be helpful to link studies with different terms that are all focused on the same concepts.

### A deficit approach

Based on our review of studies describing the extinction of unknown taxa, most employ deficit language to refer to lost biodiversity, as we will discuss in detail below. However, we found “dark extinction” to be distinctive amongst the reviewed terms, because Lambdon and Cronk’s (2020) and Boehm and Cronk’s (2021) choice of language provides the first clearly defined non-deficit named term. Additionally, their publications incorporated historical and geographical factors specific to their study location of St Helena, such as the phases of and ecological impacts from European settlers (and their more-than-human fellow travellers) on the island. These location-specific historical factors provided time-bound context for inferred, estimated and documented species extinctions (see Supplementary Table S2). The name “dark extinction” is a play on “dark matter” from cosmology: “almost certain to exist but remains theoretical” (Boehm and Cronk 2021, 1), like the anthropogenic extinctions that have already occurred, many of which will remain theoretical with no tangible corpses, carcasses, or preserved detritus. Given that there will be future extinctions of unknown species, dark extinction represents both a useful, non-deficit term in conservation biology and an accessible label suitable for discussion with the wider public.

**Figure 2.**
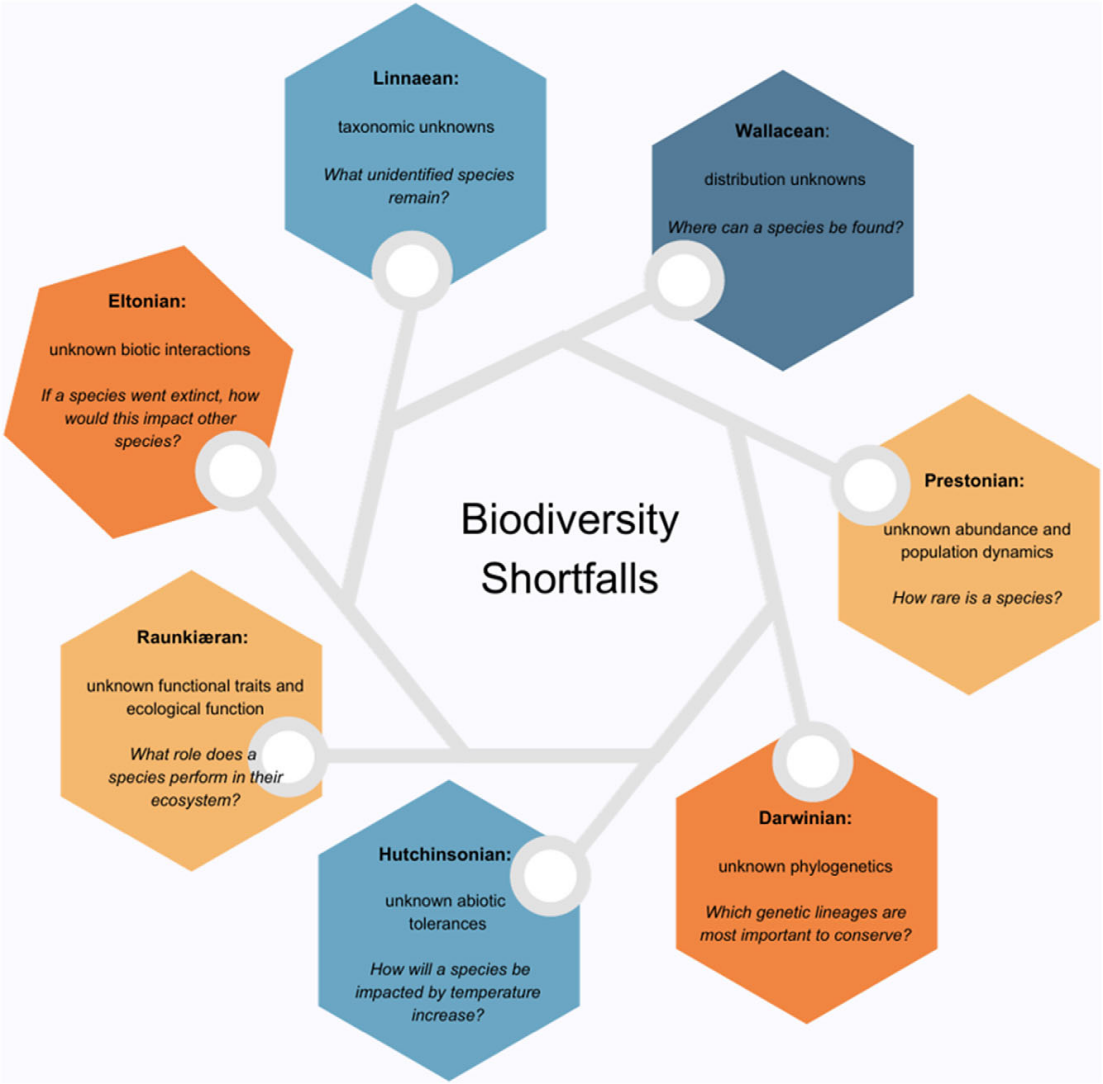
Summary of seven biodiversity shortfalls, adapted from Hortal et al.’ Table 1 (2015, 525) and wider article. Each hexagon provides the name of a biodiversity shortfall, a short definition, and an example of the type of extinction-related question that reveals the significance of that type of biodiversity data. Cornwell et al. (2019) suggest a further Venn shortfall where Linnaean, Wallacean, Darwinian and Raunkiæran intersect, arguing all four are needed for sufficient biodiversity knowledge for conservation decision-making.

Deficit language is arguably more problematic for undiscovered biodiversity since it could impact the way we think about extant biodiversity (Lupyan et al. 2020). This deficit terminology for known biodiversity comes in two forms: Firstly, research which refers to the biodiversity shortfalls outlined above (Figure 2) speaks to scientific knowledge deficits, not to the species implicated within this deficit. The second form of labels comprises specific terms for types of unknowns. Categories “cryptic” and “hidden” imply that the species is being cryptic or is in hiding. Limited to human senses, this is our perspective: we find fungi growing within another species hard to detect, so it seems that the fungi are hiding from us. However, fungi have no notion of hiding, fungi are not sentient, nor trying to be cryptic. As Fišer et al. (2018, 624) note, “cryptic species may not be cryptic to other community members”: mushroom hunters, for instance, utilise dogs to help them locate matsutake mushrooms (Tsing 2015), hinting matsutake are less cryptic to at least some nonhuman species. “Hidden” and “undiscovered” are terms placed upon species and situations to express the difficulties experienced by Western scientific, human knowledge-making in trying to find these species. It suggests the problem is with the undetected species.

The same notion of deficit language applies not just to labelling unknown species, but also to macro-focused models that highlight the absence and emptiness of our ideas. This focus means the causes of the obscurity of this life go overlooked. Crucially, if we think of unknown species in terms of absence from records, or emptiness, these species are more difficult to relate to, imagine relationships with, create policies for, value in ethical terms and ultimately, to address their loss. Van Dooren (2022) provides an example of building relationships with unknown forms of life: he can imagine extinct snails through the assistance of their taxonomic names which provide some individuality and semitangibility for him to use in snail storying. There are multitudes of nameless lifeforms potentially threatened by extinction. Until now, we lacked an umbrella term to discuss these.

## Shadow diversity

**Figure 3.**
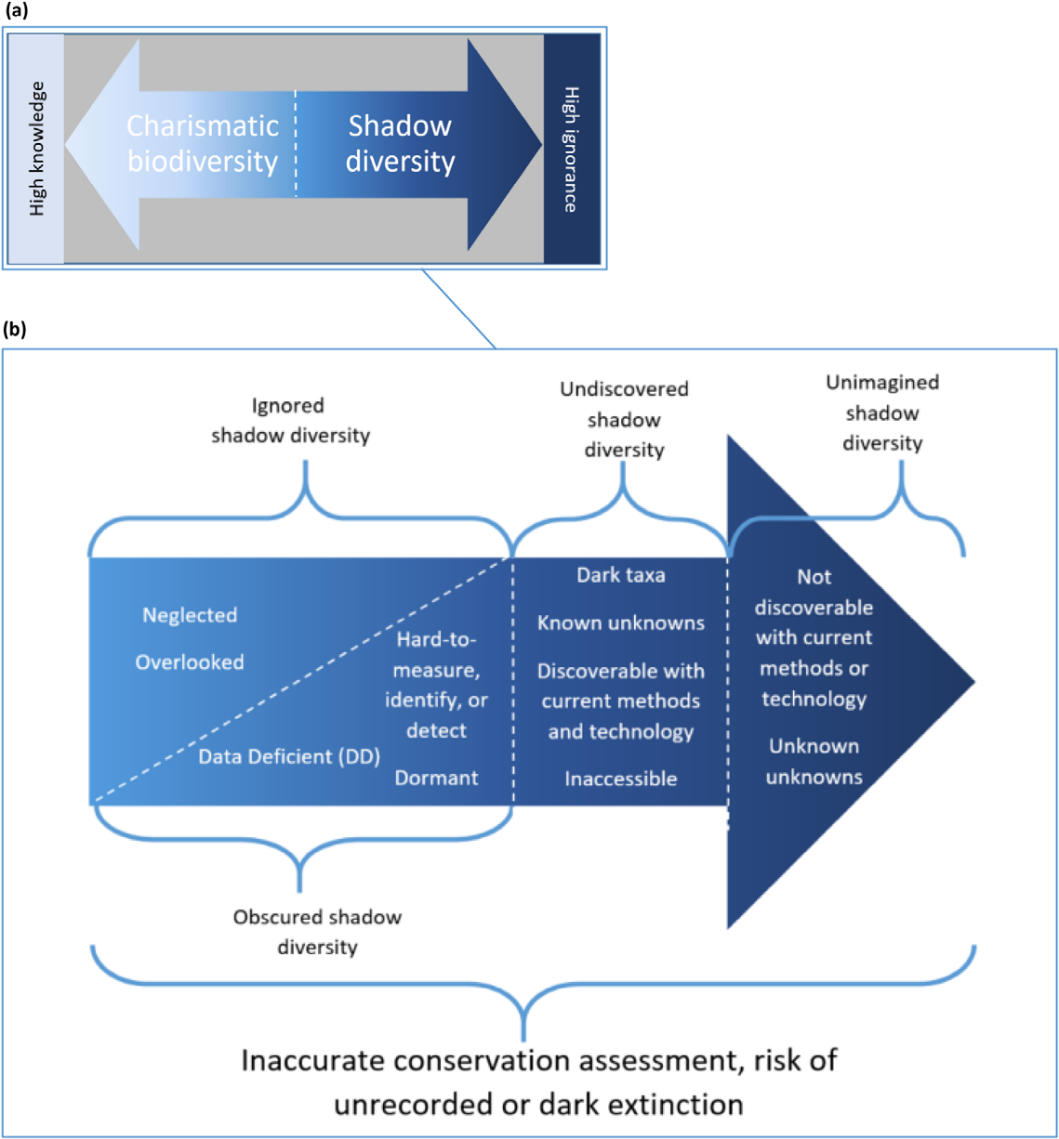
(a) The spectrum top-left shows increasingly known charismatic biodiversity extending towards the left side and shadow diversity, increasingly unknown, extending to the right. (b) We zoom in to the shadow diversity side of the spectrum, encompassing four subcategories of shadow diversity, with deeper levels of ignorance as we travel towards the right-hand side of the spectrum.

We suggest the terms and concepts from the above review of unknown extinction literature (Figure 1b) should be examined collectively as a spectrum of “shadow diversity” (Figure 3). In a linguistic sense, this new term, “shadow” acknowledges the otherness of these species more positively: shadows are caused by another object obscuring light. This shifts the “problem” from the biodiversity in question to the obscuring object. Rather than implying that the action of un-seeing is caused by the species in question, the action might be the human gaze, priorities, choices, biases, tools utilised, or methods employed to survey an ecosystem. This adjustment does not seek to remove agency from shadow species, but to remove any blame or negative connotations to these forms of life falling into shadow diversity. Employing constructive language, positively referring to forms of life previously confined to unnamed, undescribed forms, may prove helpful in shifting approaches to communicating dark extinctions and shadow diversity. Whilst the nature of much of shadow diversity means individual species may not yet or ever be nameable species, by using the term “shadow diversity” we have an appropriate, non-deficit, linguistic tool with which to discuss, both within and beyond conservation biology, life forms which are at potentially substantial risk of extinction.

In an ecological sense, we define shadow diversity as those species that are present in an area but that are not known to occur there. This definition is sufficiently precise for use by conservation biologists, yet also appropriate for wider public communication and engagement. Shadow diversity incorporates invisible, neglected, overlooked and unrecorded known or unknown species that are likely to be missed by current ecological sampling. In this sense, we have focused on taxonomic biodiversity, but the shadow diversity spectrum (Figure 3) is hopefully adaptable for others to use to discuss and incorporate all biodiversity types (genetic, taxonomic, functional). Undiscovered biodiversity cannot be categorised as homogenous “unknown unknowns” and our knowledge of undiscovered species does not remain stationary: in Figure 3 we provide labels to communicate this heterogeneity and allow discussion of movement within a spectrum of shadow diversity. We subdivide the spectrum of shadow diversity into four categories: ignored shadow diversity, which can be known but humans deem unimportant; obscured shadow diversity, where we have evidence that a species exists, but it remains undescribed; undiscovered shadow diversity, where we are aware of a lack of knowledge and actively plan to account for this, as for dark taxa, unnamed or formally undescribed species with only fragments of DNA recorded (Page 2016; Ryberg and Nilsson 2018), as well as functions performed in an ecosystem suggestive of undiscovered species; and unimagined shadow diversity, that lies completely beyond our current capabilities and awareness (see Figure 3 and Table 1 for full details). Shadow diversity is, therefore, an umbrella term that incorporates all terms in Figure 1b, but enables accessible communication of the varying levels of unknowns for different types of biodiversity.

**Table 1.** Exploring categories of ignorance from Gross (2007) in relation to shadow diversity subcategories

Term	Definition	Interpretation for shadow diversity	Example
Ignored	*Negative knowledge* is “Knowledge about what is not known, but considered as unimportant or even dangerous” (Gross 2007, 751)	*This transposes neatly onto scenarios and species that are neglected in biodiversity sampling or studies: species known to science, yet not considered sufficiently valuable or significant for measurement or study. It refers to a level of intentional choice (see Proctor 2008).*	Distribution of urban rabbits or rats. This category is in danger of unnoticed extinctions, as in the case of Berger (2008).
Obscured	Missing from Gross’s definitions and process of ignorance, we compare this subcategory to *unknown knowns.*	*For the purposes of biodiversity, this relates to species for which there are scientific records, but where practicalities prevent further knowledge or make this difficult to achieve. Examples of this would collected species in herbariums and museums, yet to be described and lacking taxonomic expertise; holding a comprehensive list of species of a certain kingdom on record, but no taxonomic expertise available to survey a region being studied (both considered forms of “taxonomic impediment”); or habitats in extreme locations where species inhabiting the area may already be known to science, but cannot be surveyed accurately due to physical inaccessibility of a site, also referred to as “habitat impediment”(Mammola et al. 2021).*	Missing data for bryophytes, orchids, asters, and begonias (Ratnasingham and Hebert 2007; Cornwell et al. 2019; Bánki et al. 2022); herbarium and museum specimens, yet to be described and lacking taxonomic expertise.
Undiscovered	*Non-knowledge* “Knowledge about what is not known but taking it into account for future planning” (Gross 2007, 751)	*This speaks to the work of predictions for undiscovered biodiversity at global (Moura and Jetz 2021) and national scales (such as Lum et al. 2021). The scientific community knows undiscovered species remain and has estimated numbers and locations for this, although they have yet to be found. This shares similarities with Kerwin’s (1993) known unknowns but goes further in the inclusion in planning and prioritising future research. This action-oriented aspect is particularly relevant for the field of conservation which is action-oriented and calls for urgent work.*	Cone Snails (Chivian *et al.* *20*03); mycorrhizal fungi (Society for Protection of Underground Networks (SPUN) 2021); likelihood of new fungal species (Banchi et al. 2018; Cheek et al. 2020); suspected additional cryptic species amongst insects thought to be generalists (Sheikh et al. 2022) and lichens (Lücking et al. 2014); habitat type: inaccessible tropical location (Scheffers et al. 2012); deep sea (Jamieson et al. 2021).
Unimagined	*Nescience* “Lack of any knowledge: a prerequisite for a total surprise beyond any type of anticipation – can lead to ignorance and non-knowledge but belongs to a different epistemic class from the above terms.” (Gross 2007, 751)	*Reserved for extreme unknown-unknown forms of biodiversity, beyond our current capabilities of awareness and only accessible in retrospect.*	Awareness during (or prior to) the 1970s of the *Chromista* taxonomic kingdom, established in 1981 (Cavalier-Smith 2018).

Shadow diversity represents not only an umbrella term for groups of taxa but also a framework within which we can critique and enhance systems of biological knowledge. For instance, we should explore why some species remained obscured, whilst others have not. Whilst heterogeneous, species falling within these components of hitherto unclearly named biodiversity share a common potential fate: a lack of accurate conservation assessment. Such species are unable to enter the conservation system that could afford them effective legal protection (Betts et al. 2020) and public interest due to the current conservation discourse centred on the IUCN Red List that requires identifying species in order to monitor them (Lorimer 2015). Undiscovered biodiversity cannot gain entry to this system until it is “discovered” and formally described via Western scientific and taxonomic protocols (Ryberg and Nilsson 2018). We can see evidence of conservationists’ frustrations towards “taxonomic impediments” (barriers which delay taxonomists’ work, such as insufficient experts and resources) where conservationists suggest alternative interim systems to speed up species’ entry to the conservation system with strikingly similar patterns in the past 40 years (Ramsay 1986, Sharkey et al. 2021). An overarching term would enable an integrated approach to species excluded from the conservation system, with better acknowledgement of sliding scales of knowledge about unknowns.

It is worth spending a moment to delineate the concept of shadow diversity that we develop in this work from other concepts associated with taxonomy and conservation. For instance, shadow diversity stands in contrast to the concepts of umbrella species and flagship species. Both concepts single out one species as a focal taxon for conservation, either to propel a conservation campaign to the public (flagship species) or with the assumption (or hope) that in protecting that one species with its habitats and ecological networks then the remaining biota will be successfully preserved (umbrella species) (Simberloff 1998). Umbrella and flagship species tend to be macrofauna, to the detriment of focusing on other important species, such as invertebrates (Cardoso 2011). Shadow diversity, by contrast, forces our gaze to the smaller, the overlooked, the microscopic life and does not try to quantify that diversity. This shift in perspective builds on work from neglected biodiversity, such as soil ecologists (Wyckhuys 2021) and deep-sea specialists (Fanelli et al. 2021). Hence, shadow diversity does not require yet more quantification of diversity, but rather a call to rethink conservation practice by critiquing the origins of these unknowns. A second instructive comparison is between shadow diversity and “dark diversity”, which is already a term in conservation biology. Dark diversity refers to (scientifically described) species that we expect to find in a given location based on ecological conditions, but which are absent from that location (Pärtel et al. 2011). It therefore focuses on understanding and quantifying the absence of known, expected species in contrast to shadow diversity, which focuses on life that is present in a location but remains unobserved, unrecorded, or unknown and the reasons for that lack of knowledge.

**Figure 4.**
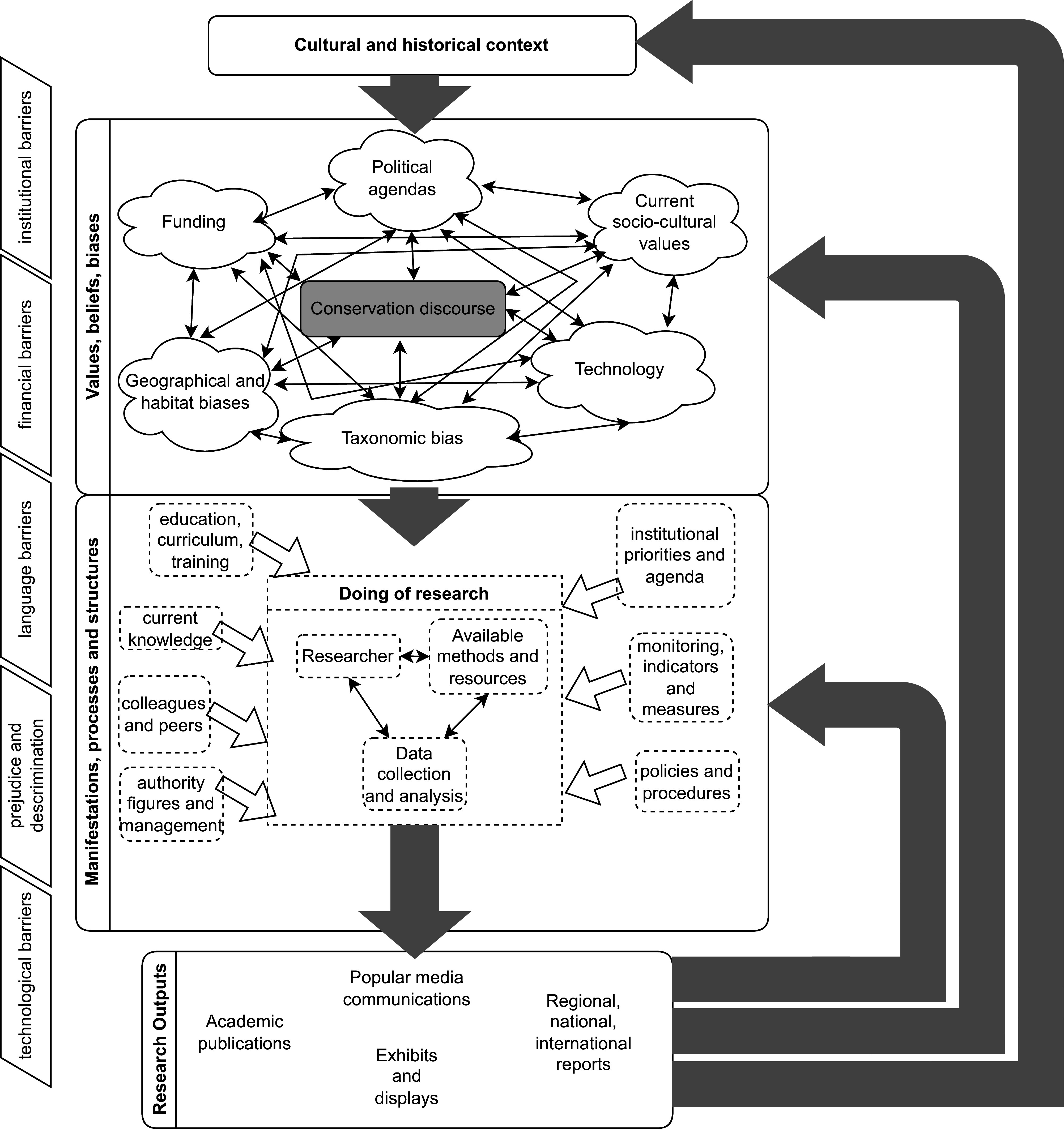
A diagram showing the cycle of factors perpetuating ignorance of shadow diversity.

Shadow diversity builds on work completed on biocultural extinction which frames culture as inextricably linked to biological extinctions (Ladle et al. 2023). Biocultural extinction seeks to understand how socio-cultural processes and values contribute to extinction. Shadow diversity asks similar questions about the origins of extant biodiversity ignorance, albeit shadow diversity sees a more dual system of influence where culture influences conservation, as per biocultural extinction, but conservation practices also influence culture (Figure 4). Finally, whereas biocultural extinction aims to measure the cultural value of species at risk of conservation, shadow diversity aims to change the perception of how we can engage with unknown biodiversity.

In addition to accurate data regarding how many species remain and where they are, there are numerous examples where studying previously neglected biodiversity has demonstrated a significant ecological role of that species, which had been overlooked. For instance, a review of pollinator projects revealed that non-syrphid *Diptera* (flies excluding hoverflies) are important pollinators (Orford et al. 2015). Recent studies of underground and phyllosphere microbiota highlight the important ecosystem functions performed by previously unknown species, including nematodes, fungi and bacteria (Mercado-Blanco et al. 2018; Custódio et al. 2021; Chialva et al. 2022). Looking ahead, many functions and ecosystem services have yet to be realised. Fungi are a good example of such unknown potential, such as “mycomediation” which uses currently known fungi to decompose harmful agents in the environment (Harms et al. 2011; McCoy 2016). It is highly likely that there will be many future uses of currently unknown fungi, given estimates suggest 90 percent of fungi remain undiscovered (Cheek et al. 2020) and predictions that these fungi will be useful for future medicines (Hyde et al. 2019).

Behind arguments for improving conservation outcomes, lie arguments around our limited understanding of global biodiversity (Treurnicht et al. 2017; Fisher et al. 2018; Stork 2018; ter Steege et al. 2020; Moura and Jetz 2021). Despite awareness of these shortfalls, factors such as false absence and undetected species (Chao et al. 2017, 2020) frequently remain excluded from smaller-scale community ecology estimates (Devarajan et al. 2020; Richter et al. 2021). Not only are more local biodiversity estimates therefore not showing the full picture, but this is not always being accounted for in studies used to inform conservation decisions. Despite arguments for the importance of studying shadow diversity, despite awareness of biodiversity shortfalls and practical steps to remedy these, what remains to be explored are the deeper reasons why shadow diversity continues to be neglected by researchers, funders and wider society.

## Origins of shadow diversity

### Existing discussions

An overview of shadow diversity literature (Supplementary Table S3) revealed numerous gaps in fully connecting causes for overlooking types of lifeforms with their wider contexts and impacts of these contexts. We will outline those issues here, showing why a broader and deeper multidisciplinary perspective is needed to understand why conservation data is more complete for some areas of the Tree of Life.

The majority of reviewed literature focused on themes of technical and practical reasons why some species have been described and studied for longer with more effort. These reasons include our knowledge of taxonomic boundaries and the technical aspects of determining those boundaries (Fišer et al. 2018; Chenuil et al. 2019) and taxonomic impediments (Engel 2021). Similarly, Hortal et al. (2015) cite practical elements of methods and available technology as limiting factors to human knowledge, whilst other studies focus on overcoming these practical technological limits, as in the case of increased species descriptions due to molecular sequence data (De Clerck et al. 2013). In some cases, we can infer social factors from elements such as the high rate of cryptic species identified in temperate regions, linked to the location of taxonomists and resources (Bickford et al. 2007), yet those social factors must be inferred since they are not explicitly discussed.

A second less common theme in the literature was the roles played by individual taxonomists and researchers. For example, findings in taxonomic disparities for genomic sequencing of non-human primates suggested a combination of factors influenced the choices of the researcher, including the feasibility of a study, and interest in the species (Hernandez et al. 2020). De Clerck et al. (2013, 217) emphasise the key contributions of a few prolific, named taxonomists in describing algal species. Their analysis overlooks what might have enabled those particular male German, Russian and Irish taxonomists to provide disproportionate species descriptions. These issues are relevant when we know, for instance, that during the nineteenth-century surge in the taxonomic description of species, women were excluded from formal education, the professionalization of science and scientific publication (Maroske and May 2018). One example is the noted British author Beatrix Potter: as a female, self-studying lichenology and mycology in nineteenth-century Britain, she was excluded from the Linnaean Society of London due to being female, despite her advanced illustrations, microscopy, studies of fungi and lichens and submitting a paper in 1897 (Schmid 1999; Watling 2000; Breedlove 2019). This is an example of historical context, discrimination and prejudices contributing to who became or did not become, professional taxonomists. Thus, who was encouraged and able to become part of the “disproportionate taxonomic effort” was not determined purely by chance or interest. Overall, superficial or proximate explanations for variation in the rates of species discoveries miss historical context and potential root causes for what might initially seem objective practicalities of location (Moura et al. 2018) or individual preferences.

We can find engagement with more socially focused causes in studies that concentrate on specific taxonomic groups and scenarios rather than macro-studies. For instance, Blackwell (2011, 434) discuss increased interest in medical mycology due to a rise in fungal infections caused by AIDS cases, along with an increase in chytrid systematics driven by amphibian biodiversity loss, attributed to this fungal group. There is no discussion, however, explaining how concern with fungal infection feeds back into the system of taxonomic and conservation knowledge creation. In a further example, De Clerck et al. (2013) cite World War I and World War II as factors for explaining changes in the rate of descriptions of algal species. De Clerck et al.’ consideration is not extended, however, to discuss how or why wars impacted algal species discoveries or algal taxonomists; they are treated as isolated practicalities. Still, consideration of such questions is pertinent given that other areas, such as mycorrhizal fungi, continued to see research and publications through the wars and interwar period (for instance, Rayner and Neilson-Jones 1944), suggesting that other factors impacted which species discovery rates accelerated or decelerated from 1914 to 1945.

A small number of previous studies within the literature on species discovery rates and taxonomic bias include social and cultural causes for species discovery and non-discovery. However, social causes are constrained as isolated, often quantitative, factors, such as proximity to a research institution (Meyer et al. 2015), human density and the number of taxonomists working in an area (Moura and Jetz 2021). These factors are treated separately and there is no deeper exploration of what may have caused more taxonomists to work in a given location, nor the powers, legacies and traditions that accompany research institutions, such as the imperial legacy of Kew Botanical Gardens (Supplementary Table S4). In a similar fashion and using quantitative methods, Stefano et al. (2023) emphasised socio-cultural causes of the uneven distribution of studies and interests in different species. However, many of the socio-cultural elements hold a practical focus, such as utility to humans. Stefano and colleagues rightly note that aspects of utility can be linked to culture, but they provide no more depth around how different cultures may impact views or values of utility, such as Brown’s investigations of valuing different species of fungi in the Yunnan Province of China (Brown 2019). Finally, focusing on a lack of care for deep sea knowledge by non-scientists, Jamieson et al. (2021) consider some psychological, philosophical and ethical factors as causes for the unpopularity of deep-sea conservation. However, they dichotomise between the objective scientist and subjective non-scientist: “While scientists approach the deep sea in a highly objective way…the layperson is more likely to develop different perspectives based on alternative, nonscientific sources” (Jamieson et al. 2021). In their work, scientists retain immunity to these factors, working to correct misinformation and cultural myths of non-scientists, rather than acknowledging scientists as individuals impacted by some of the same factors. This is despite work that points to the importance of history, culture and education in values people construct of species (Czech et al. 1998; Genovart et al. 2013; Heise 2016) and the choice of study species (Wilson et al. 2007).

What is missing is a consideration of the complex underlying societal and individual factors that contribute to the formation of values and motivations (see Eccles and Wigfield 2020). With these exceptions noted, direct, pragmatic considerations, such as species accessibility (Mammola et al. 2021), take precedence in other academic literature. We agree that such practicalities as new methods and technology play a significant role. However, our review adds to this dialogue by adding multi- and interdisciplinary root causes of shadow diversity and using this understanding to provide new solutions, avenues of research and insights for approaching biodiversity conservation which account for a network of deep historical and socio-cultural factors.

### A deeper, multidisciplinary approach

Various disciplinary approaches have been used to explore why ignorance exists. Smithson’s review of ignorance notes “ignorance can be understood only in reference to a social context, and that a great proportion of ignorance is created and maintained by people themselves, rather than being imposed on us by an intractable universe” (1989, 219). The materials we have reviewed suggest methods already exist that might enable us to better study shadow diversity. This includes technological advances, particularly DNA barcoding and metabarcoding from environmental DNA (eDNA), which began in the 1990s (Giovannoni et al. 1990). These methods provided new ways to sample for and identify taxon. New methods for imagery, such as microCT scans (Stoev et al. 2013; Akkari et al. 2015), make previously hard-to-see species more visible, and acoustic monitoring provides nonvisual methods for data sampling (Mooney et al. 2020; Parsons et al. 2022; Wood et al. 2021). Despite these methods, taxonomic bias toward studying well-known species persists (Titley et al. 2017; Stefano et al. 2023). In other words, shadow diversity and anticipated dark extinctions with it will not be solved by overcoming practical limitations and new methods alone; we must additionally consider deeper factors that impact how species are valued by societies, academic disciplines and individuals.

To account for the persistence of taxonomic and conservation biases, Figure 4 shows a complex web of factors that act in a cyclical nature. We are not the first to point to cyclical, self-perpetuating knowledge in the Western conservation system (Lorimer 2015) or taxonomic knowledge (Stefano 2023). Beyond conservation, we note the traditions in history, philosophy and science studies to explore the historicity and cultural origins of ignorance (Proctor 2008; Schiebinger 2008) and acknowledge the scientist as an individual tied to their experiences of society as a human (Latour 1993, Prescod-Weinstein 2022). Our diagram, however, is distinct in showing the role of wider historical, socio-cultural and political factors in this looped system in conservation, and to this end, we hope it will be of use to highlight the multidisciplinary effort required to explore shadow diversity at the edge of ignorance. Whilst this model is not exhaustive and inevitably takes a broad and therefore somewhat generalised view, its purpose is to illustrate, first, the range of factors influencing knowledge-making around the study of species, ecology and conservation; second, how some of these factors interact; and thirdly, the feedback loop inherent within the social, cultural and political networks which have the potential to exacerbate pre-existing biases within the system if left unchecked.

The overall cycle in the diagram (Figure 4) shows the importance of historical context in informing values, beliefs and biases with which the conservation discourse is imbued (Heise 2016). This, in turn, informs the structures and manifestations of the conservation discourse: the predecided content of an ecology module’s curriculum, the biodiversity indicators and the institutions, documents and individuals which hold power. Working under and within all of these factors and affected by them on multiple levels (Czech et al. 1998; Eccles and Wigfield 2020), is the “doing of research”: the individual people, methods and tools used to generate “new” data. It is this doing of research, spurred by requirements for meeting targets, funding, personal and institutional requirements and personal desires to share new findings, which leads to the formation of research outputs. These in turn provide materials that feed into political thinking, challenging or supporting previously formed views.

The nature of the system makes unorthodox thinking or research difficult and leads to a pattern of studying what has already been well-studied because it has supportive infrastructure, colleagues and knowledge records already in place. To the left, Figure 4 gives examples of barriers, for people and ideas whose entry into the system of knowledge is impeded. Barriers of prejudice and discrimination based on language, colonial legacies and non-western forms of knowledge in access to academic publishing and power have been documented elsewhere (Canagarajah 2002; Lillis and Curry 2013; Politzer-Ahles et al. 2020; Hernandez 2022; Sumida Huaman 2022). It is beyond the scope of this article to give copious examples here. Instead, we provide a range of references and examples to accompany and support this diagram in Supplementary Table S4. Below, we will present a worked example of the rise in understanding and study of mycorrhizal fungi to illustrate the dynamics of some of the factors shown on the diagram.

### Worked example: mycorrhizal fungi

Prior to the 1850s, mycorrhizal fungi fell under unimagined shadow diversity and we have good historical records of its move leftwards on the shadow diversity spectrum (Figure 3). Mycorrhizal fungi are now known to be a symbiotic partnership between a colonised plant root and its colonising fungus (Smith and Read 2007) in the majority of terrestrial plants. They demonstrate one characteristic of shadow diversity, in that it is hard to see the fungus-root interface with the naked eye and is less well-known to the public due in part to its subterranean nature (Tsing 2015, 139). The discovery of mycorrhiza required technological elements, such as the microscope. The botanist, Frank, also played a key role being credited with preparing detailed drawings and the theory which recognised the symbiotic nature of the fungus-plant partnership (Trappe 2005). However, multiple factors created the circumstances under which this discovery was made, so moving mycorrhizal fungi to everyday ecosystem discussions today. For instance, Frank’s research was funded by the German State Forestry Department with the intent to commercialise truffle production in Prussia (Ainsworth 1976, 100), thus revealing factors of funding; the cultural and economic value of truffles in that place and time; and a political situation which provided means, infrastructure and impetus to fund such a venture.

Once Frank’s discovery had been made and published in 1885, social and historical contexts caused a slow uptake of the idea of a mutual relationship between plant and fungus (Rayner and Neilson-Jones 1944). For Frank’s peers, overarching negative attitudes towards fungi had persisted for centuries in wider society, where fungi were seen as lower forms of plants, such as the famous descriptor of fungi as “excrement of the earth” (Thacker 1758, 67). This contributed to a poor reception of the notion that fungi could be anything other than pathogenic to plants. Two further contextual factors illustrate the controversial nature of Frank’s assertions: Firstly, although fungi formerly remained under the plant kingdom until 1969 (Whittaker 1969), contemporary amateur and professional botanists already recognised fungi as distinct from other plants and cryptograms. In 1864, for example, Plues used separate sections for mosses, ferns and fungi in her book *Rambles in Search of Flowerless Plants* (Plues 1864). Secondly, Frank’s work appeared 26 years after Darwin’s 1859 *Origin of the Species* when the biological principle of competition between species was gaining attention. In this context, Frank’s suggestion that two different organisms, assumed to be in competition, were actually operating to the mutual benefit of one another, was highly controversial. This in turn forms part of a wider struggle through time for acceptance of symbiosis, over competition-based species interactions (Gontier 2016). Frank persisted in his research and publications (Trappe 2005) and the notion of mycorrhizal fungi as valuable aids to their host plants slowly developed. Visibility of this value, however, was initially quite restricted to a specialised scientific community (see Ainsworth 1976; Bonfante 2018; North American Conference on Mycorrhizae 1985).

Our journey now reaches a different turning point in the rise of mycorrhizal fungi’s status and knowledge in science: the work of Francis and Read (Francis and Read 1984) and Simard (1997). Francis and Read showed the transfer of carbon between plants through fungi and Simard’s work showed this was happening beyond laboratory experiments, in real forest ecosystems, between different species of trees with different quantities of carbon transferred depending on the situation of the receiving tree (Sheldrake 2020, 168–169). Simard mentions personal struggles with peers in her forestry network in North America, who were heavily invested in free-to-grow policies, which supported the eradication of other species not selected for cultivation (Simard 2022). However, in the wider scientific community, reception for these works was vastly different and was received more positively overall compared to that received by Frank a century earlier. Sheldrake emphasises the serendipitous timing of Simard’s work with network theory, the expansion of the internet, combined with the metaphor of “wood wide web” (Sheldrake 2020, 170). Nixon (2021) argues that society’s hope for mutualistic interspecies forest relations was a reaction to neoliberal capitalism. In addition to these sociocultural elements suggested by others, we argue the situation had changed significantly due to several rounds of conservation knowledge production via the process shown in Figure 4.

Firstly, infrastructure, in terms of institutions, organisations and communicated written knowledge grew via multiple rounds of conservation knowledge production (Figure 4). For instance, Smith and Read (2007, ix) note the profound impact of Harley’s first book dedicated to mycorrhizal fungi in 1959, providing a guide for new researchers entering the field. Similarly, a more positive reception for Francis and Read’s and Simard’s work was in part due to the respectability of studying mycorrhizal fungi: appropriate academic journals welcomed mycorrhizal knowledge, partially under Harley (a well-respected botanist with a specialism in mycorrhizal fungi), who became an editor of *New Phytologist*, making it a key place for mycorrhizal fungi publications (Bonfante 2018, 984) and increased the respectability of mycorrhizologists (Koide and Mosse 2004). Finally, economic factors further bolstered impetus and interest in mycorrhizal fungi. The first half of the twentieth century saw a rise in appreciation for mycorrhizal fungi, due to their potential in improving forest productivity and subsequent financial benefits from this. This was especially important for Western Europeans during settler colonialism, planting fast-growing pines on ill-suited soils for this purpose, and replacing timber stocks following world wars (Rayner and Neilson-Jones 1944; Harley 1957).

Mycorrhizal fungal knowledge also includes examples of barriers to entry in action: we glimpse the potential exclusion of non-western-scientific knowledge when Simard (2022, 66) gives an example of fungal mutualism known by Coast Salish people. Due to the oral nature of this knowledge system, we cannot be sure when the earliest understanding of symbiotic relations occurred beyond the Western scientific knowledge systems and conventions, but it seems likely this originated before the nineteenth century. Precluded from entry into the current knowledge on fungi by language, cultural barriers, power imbalances from the colonial takeover of North America, and prejudices instilled in settler-colonists that alternative modes of knowing were inferior, such knowledge is only recently beginning to gain recognition.

Overall, we can see how utilising this model can support a rich understanding of the complexities behind discoveries and hope this diagram may be useful to inspire further multidisciplinary explorations regarding why some parts of the Tree of Life are better known than others. To conclude Figure 4’s looped system: the origins of shadow diversity are inherently multi- and interdisciplinary. Investigations into shadow diversity need to mirror this: it is not sufficient to examine biological methods, entirely separated from the societies, structures and people utilising those methods.

## Challenges and directions for further research in shadow diversity

Biodiversity shortfalls discussed earlier provide a breakdown of the challenges of biodiversity ignorance for conservation and conservation scientists. Examining shadow diversity, for conservation scientists, researchers working across other disciplines and the general public, brings its own set of challenges that will need to be addressed. The greatest challenge of shadow diversity is the problem of handling unknown unknowns, or unimagined shadow diversity. This is a pragmatic issue regarding how to detect the undetectable, yet it is also a cognitive challenge in how to conceptualise total unawareness. These unknowns go beyond individual minds and into communities of practice and knowledge: conservation biology, biogeography and ecogeography communities, for example, have an awareness of the limitations of known biodiversity (Lomolino 2004; Hortal et al. 2015). We, therefore, utilise Gross’s (2007) A sociological exploration of knowledge and ignorance, as this offers nuanced understandings, but also helps to capture the dynamism and at times active processes involved in ignorance and knowledge-making, see Table 1.

Returning to our challenge of unknown unknowns, firstly, we can see that by using a spectrum and categories within shadow diversity (Figure 3), we can be more specific about how undetectable a type of biodiversity is. Whilst some deep-shadow diversity falls into nescience, numerous categories are within reach. We can visualise the movement of a species from non-knowledge to unknown knowns as it is discovered to be two or more genetically distinct species (previously a cryptic species). Echoing van Dooren’s (2014) call to work at the “edge of extinction”, we can further acknowledge that understanding the boundaries of our knowledge is itself a step towards discoveries and knowing. The unknowns of shadow diversity therefore span nescience and ignorance (Figure 3, Table 1). These specific types of unknowns highlight four challenges of working with shadow diversity, which will be outlined below. Throughout, the key is mapping boundaries of known unknowns for future use.

### Challenge 1: Cognitive challenges of handling incomprehensible life forms

There are limits and differences to human senses compared to nonhumans. Examples of this include insects’ ability to see ultraviolet light which the human eye cannot (Cronin and Bok 2016; Mazza et al. 2010); human inability to detect and interpret chemical signals released by plants (Manetas 2012, 181–182); and the ability of *Anopheles gambiae* (malarial mosquito) to detect carbon dioxide (Jones et al. 2007), which unaided humans cannot. The limits of human senses mean the way we interact with the world provides us with different cognitive experiences compared to nonhumans and our senses cannot detect signals that might otherwise alert us to the presence of other life. Our senses make us prone to overlooking forms of biodiversity invisible to the naked eye, or where their life cycle means they are easily overlooked by humans, such as desert plants with long, dormant lifecycles (Carrasco-Puga et al. 2021). The lack of direct experience with shadow diversity presents problems for phenomenological approaches to knowledge, which require direct experiences (Rehorick and Bentz 2008, 3). In addition to this, measurability is arguably a key feature of biodiversity (Sarkar 2019). If we cannot sense it, we cannot measure it. This raises questions of whether it is possible for us to meaningfully engage with that which we cannot sense. Some of the new ecological techniques, such as eDNA discussed in A deeper, multidisciplinary approach section, seem ripe for exploring the boundaries of what we can sense and how.

### Challenge 2: Ineffability of mid- and deep-shadow diversity

“Die Grenzen meiner Sprache bedeuten die Grenzen meiner Welt” (Wittgenstein 1922/2010, 144), translated to English as “the limits of my language mean the limits of my world” (Wittgenstein 1922/2010, 74). This quote relates to the notion that since we use language to consciously think, if we do not have language, we cannot think about it and therefore it cannot exist to us. The absence of language impacts public engagement (Richling and Bouchet 2013) with nameless shadow diversity, particularly given the evidence that language impacts the way we perceive what we see (Lupyan et al. 2020).

Language limitations share parallels with the knowledge-making system for biodiversity (Figure 4) in that it is only once a species is catalogued and named by science that it enters the system to qualify for conservation assessment and potential protection (Lorimer 2015). In this case, we echo Wittgenstein by saying the limits of binominal nomenclature mean the limits of our current conservation system. The lack of scientific names and records for species has been a repeated “taxonomic impediment” which has arisen from genetic sequencing (Ryberg and Nilsson 2018; Zamani et al. 2022a, 2022b). Examining NGOs (non-governmental organisations) and datasets that deal with conservation, language plays an important role in classifying a species’ conservation status (IUCN 2022) and in placing a species in its “correct” place on the phylogenetic tree in its taxonomic kingdom (Bánki et al. 2022; OneZoom 2022). Life forms that cannot be classified for extinction risk due to lack of data are labelled “data deficient” (DD). Prior to “shadow diversity”, there was no positive way of referring to this missing biodiversity within the system. This extends beyond the conservation system and entangles public perception of biodiversity and what we are able to mourn for (Barnett 2019).

Without stories, categories and labels of individual species, it is unclear how to communicate meaningfully about shadow diversity so that someone without highly specialised knowledge can allow shadow diversity to enter their imagination, a problem highlighted by Bastian’s (2020) work on unknowns. However, absence of language does not need to be so limiting: perhaps, it provides opportunities to explore language used in other forms of knowledge. One exemplar is Robin Wall Kimmerer’s examples of “the grammar of animacy”, providing an innately different approach to conceiving other living beings around us (2015, 55–59) through their movements not just their names, and new ways to consider the movement of species through learning the Anishinaabe word “Puhpowee” from Keewaydinoquay’s work, meaning “the force which causes mushrooms to sprout from the earth overnight” (Kimmerer 2015, 49). There is also the potential for working with known species of similar types to those we do not know, to bring us closer to the edge of our knowledge, as van Dooren (2022) exemplifies in his explorations of lost snails. How we communicate shadow diversity, particularly beyond conservation experts, may feed into the conservation system for knowledge (Figure 4) in new ways and is, therefore, an area deserving of further exploration.

### Challenge 3: Valuing and caring for unknowns

As discussed earlier, academic papers have suggested that shadow diversity may hold resources of value to society (Wheeler et al. 2012). Until they are fully mapped and discovered, this is only speculation and cannot be easily valued within ecosystem services. Due to the Raunkiæran shortfall of shadow diversity (Hortal et al. 2015; see Figure 2), we can only speculate how the loss of ecosystem services might interact with other extinction risk factors. Our not knowing about these functions does not mean they do not exist, as is exemplified by studies that highlight species previously neglected but providing key ecosystem functions such as pollination (Orford et al. 2015; Lopes et al. 2021). How we care for parts of ecosystems we do not know exist presents a dilemma for how we attribute value to unquantified biodiversities. Puig de la Bellacasa’s (2015) research explores human interactions with soil as a form of care, thus early investigations into valuing a form of shadow diversity have begun.

### Challenge 4: Cognition to tangible action

One potential necessary element of defining the bounds of biodiversity is that of measurability (Sarkar 2019) and this is particularly relevant regarding conservation policies, such as the Aichi targets (Mcowen et al. 2016) and the most recent Kunming-Montreal Global Biodiversity Framework (Convention on Biological Diversity 2022). Shadow diversity’s nebulous qualities are challenging for policies that require measured progress and targets. When handling potential and probabilities for new biodiversities, how can we advocate for shadow diversity so that it warrants reflection in conservation policies, despite inherent uncertainties? We do not advocate creating new quantitative estimates or models for shadow diversity. The challenge instead is to consider tangible actions that might arise from inherently non-tangible parts of shadow diversity.

## Implications of shadow diversity for conservation

**Figure 5.**
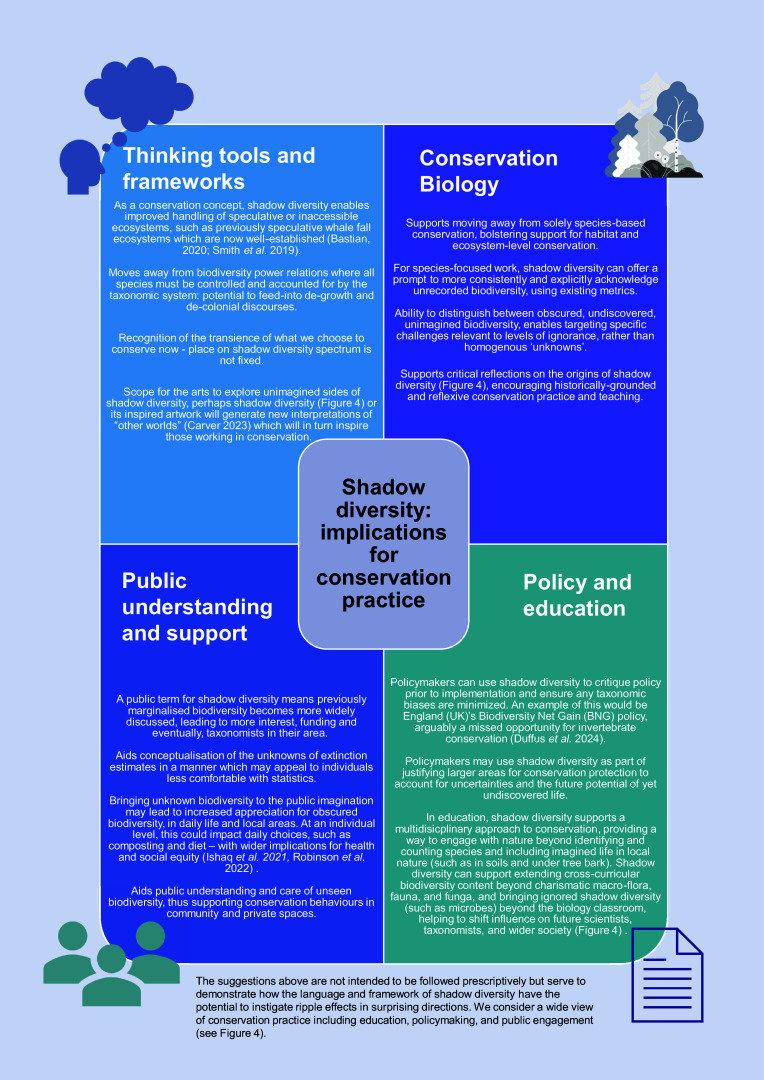
A diagram showing specific examples of shadow diversity as a useful tool for conservation, covering a spectrum from small-scale, individual, through to metacognitive discourses at wider levels.

Whilst shadow diversity includes numerous conceptual and practical challenges, it also provides opportunities for various shifts in conservation and beyond. We aim for shadow diversity to act as a useful catalyst for further discussions and creativity regarding how to use this term and framework. We offer a range of suggestions in Figure 5 for how implementing shadow diversity as a term and a framework can benefit conservation biology and beyond. It is insufficient and impractical to demand ecologists and those working in conservation sciences ‘solve’ shadow diversity alone: it has come about from society-wide, culture-wide and history-wide factors. Failure to engage in multidisciplinary approaches to improve awareness of shadow diversity will result in continued neglect of those domains of life.

The continued existence of shadow diversity is not, we suggest, necessarily a problem for solving outright, but rather a way of thinking to add to our conservation toolkit. This way of thinking strengthens existing calls to abandon species-based approaches to research. For example, soil scientists argue for a shift to food-web-based approaches to research to overcome previous taxonomic biases (Wyckhuys et al. 2021). Where more taxonomic approaches to research are needed or used, such as in nationwide conservation strategies, shadow diversity can be employed as a reflexive tool to check for adequate coverage of various groups of life (Figure 5), echoing calls made on behalf of a historically underfunded taxon, such as fungi (Oyanedel et al. 2022) and a more conscious employment of the factors perpetuating ignorance of shadow diversity (Figure 4). By grouping 13 terms (Figure 1b) under the umbrella of shadow diversity, complementary concepts are more visible and we can more readily share advances in one area of shadow biodiversity as a potential model for elevating greater inclusion for other lifeforms still deeper in shadows (such as success in momentum for improved inclusion of fungi, outlined in Haelewaters et al. (2024)). Rather than discussing “unknown unknowns”, shadow diversity further provides shared linguistic means (Figure 3 and Table 1) to distinguish more precisely the *extent* to which a particular area of biodiversity is unknown and for whom, compared to the overlapping and unclear set of terms highlighted by our review (Lack of clarity section). Shadow diversity also supports wider public engagement through accessible terminology to articulate unknowns, as well as recognise the role of wider society in shaping what biodiversity remains unknown (Figure 4), thus supporting arguments to routinely engage the wider public more systematically in soil biodiversity (Byrne 2022), for instance.

## Summary and further research required

The literature reviewed for this overview review suggests firstly that conservation biology and numerous specialist fields including ecogeography, biogeography and macroecology are aware of shortfalls in biodiversity data, which means a recognition of our inability to “complete the picture” of biodiversity is already in place. We argue that the term “shadow diversity” is a useful way of referring to missing and undiscovered biodiversity. Shadow diversity provides a comprehensive term to explore multidisciplinary aspects of anticipated dark extinctions and neglected organisms. Whilst some work has been done that begins to highlight socioeconomic causes for shadow diversity, we have argued a wider web of root causes, including historical, sociocultural and biopolitical factors also impact reasons why some biodiversities are left understudied despite the availability of methods to investigate them. The multidisciplinary nature of the causes of shadow diversity makes a compelling case for a multidisciplinary approach to further research in this area.

Dealing with nescient levels of unknowns may seem like an impossible task. However, rewards from progress regarding current total unknowns can be most impactful for the progression of scientific knowledge (Loxdale et al. 2016). We hope that this review and subsequent research will lead to a greater focus on overcoming human limitations through a richer understanding of shadow diversity’s origins and by shifting our language to reflect this. Work in extinction studies leads us to suggest there will need to be innovative approaches to slow the extinction crisis. Van Dooren (2014) uses the notion of working at the edge of extinction. We suggest working at the edge of ignorance of extinction through shadow diversity may become a useful part of this approach.

## Supporting information

Turton-Hughes et al. supplementary material 1 Turton-Hughes et al. supplementary material

Turton-Hughes et al. supplementary material 2 Turton-Hughes et al. supplementary material

Turton-Hughes et al. supplementary material 3 Turton-Hughes et al. supplementary material

Turton-Hughes et al. supplementary material 4 Turton-Hughes et al. supplementary material

Turton-Hughes et al. supplementary material 5 Turton-Hughes et al. supplementary material

Turton-Hughes et al. supplementary material 6 Turton-Hughes et al. supplementary material

Turton-Hughes et al. supplementary material 7 Turton-Hughes et al. supplementary material

## Data Availability

The authors confirm that the data supporting the findings of this study are available within the article and its supplementary materials.

## Supplementary material

The supplementary material for this article can be found at http://doi.org/10.1017/ext.2024.21.
