# Supplementary figures and images for: The diversity of ignorance and the ignorance of diversity: origins and implications of “shadow diversity” for conservation biology and extinction
Source: Camb Prism Extinct. 2024 Nov 22;2:e18. doi: 10.1017/ext.2024.21 (PMC11895729; doi:10.1017/ext.2024.21)

Figure 1:

(a)

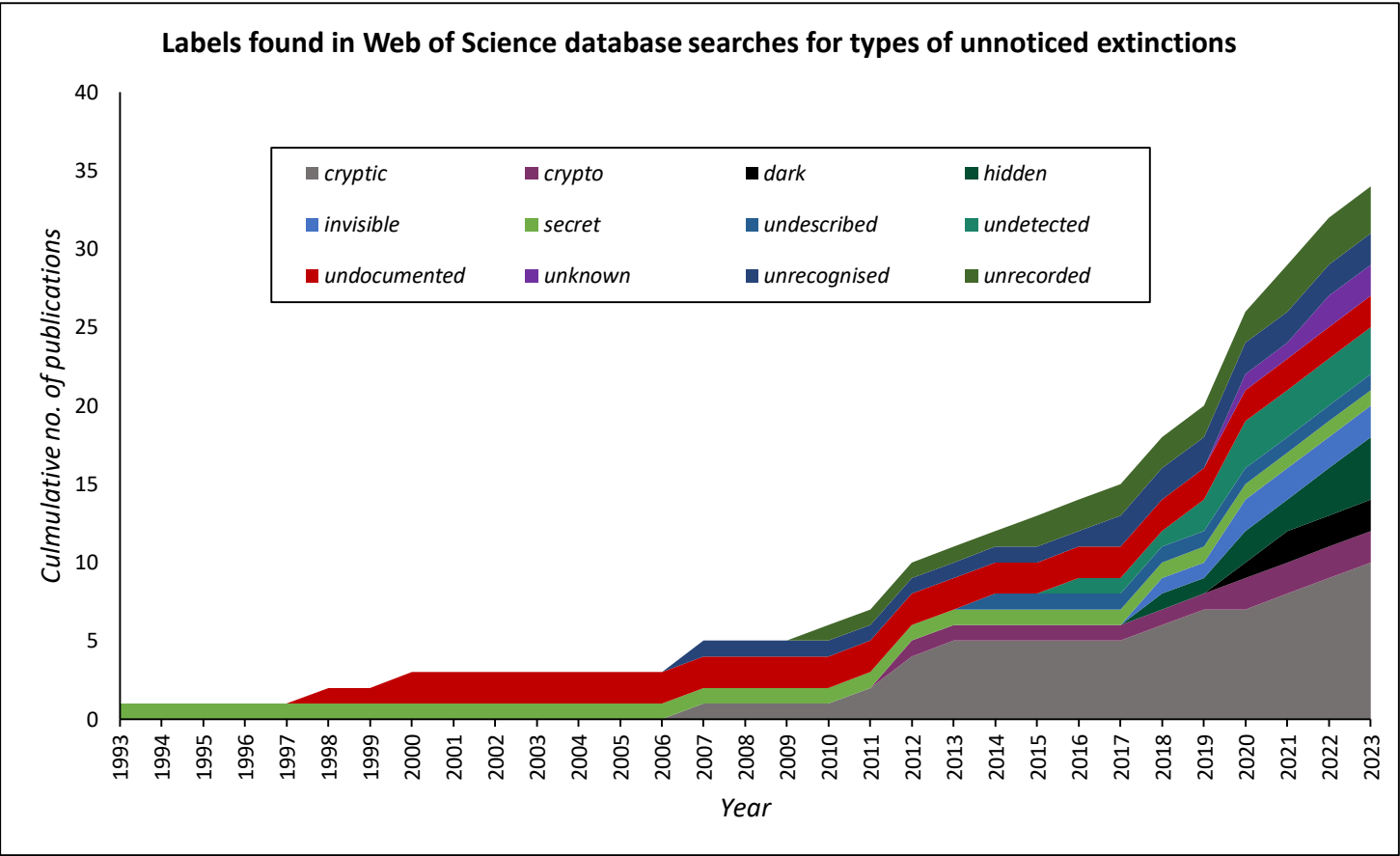

(b)

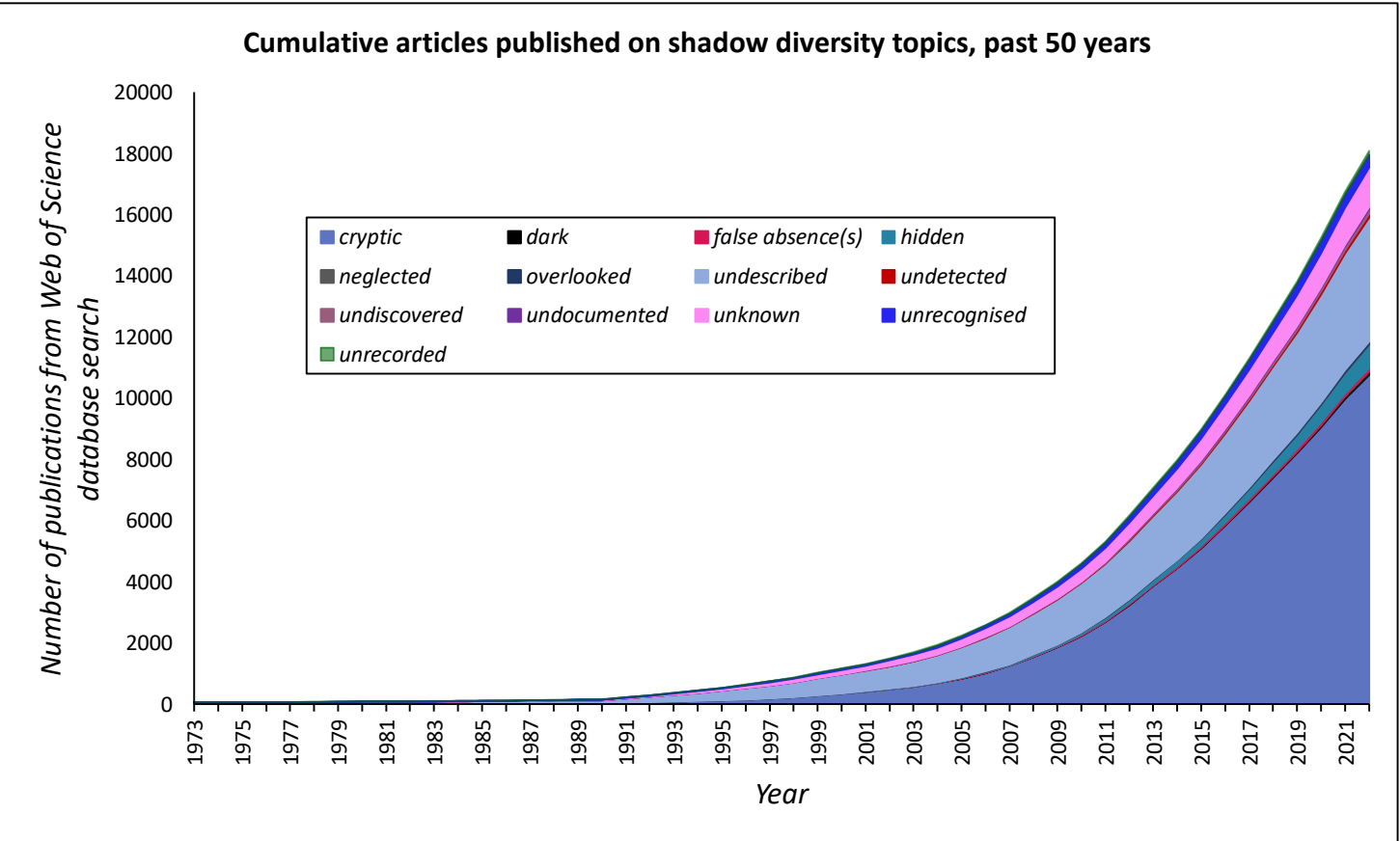

Supplement: Turton-Hughes et al. supplementary material 1 — Turton-Hughes et al. supplementary material [file S2755095824000214sup001.pdf]

Figure 2:

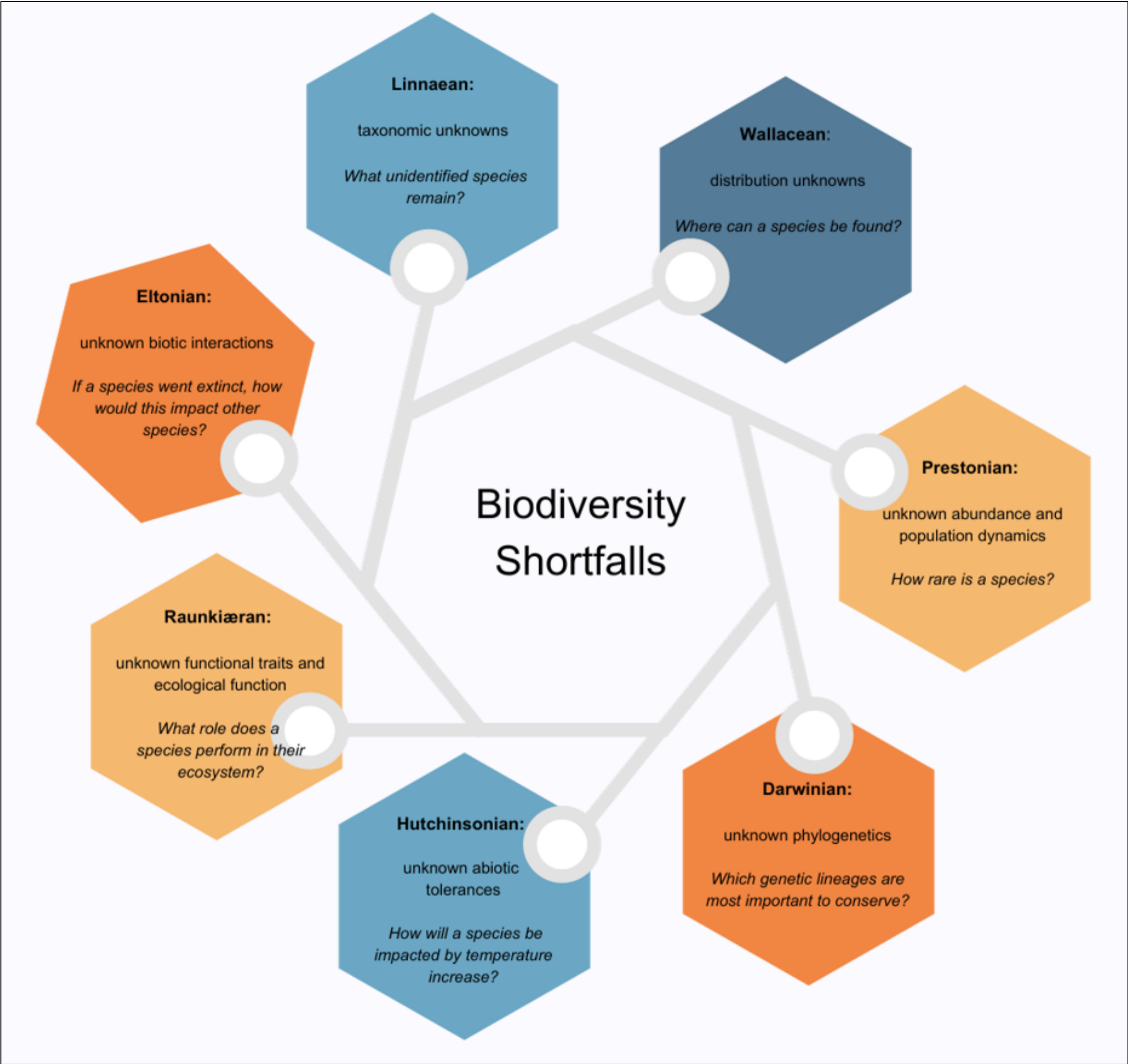

Supplement: Turton-Hughes et al. supplementary material 2 — Turton-Hughes et al. supplementary material [file S2755095824000214sup002.pdf]

Figure 3:

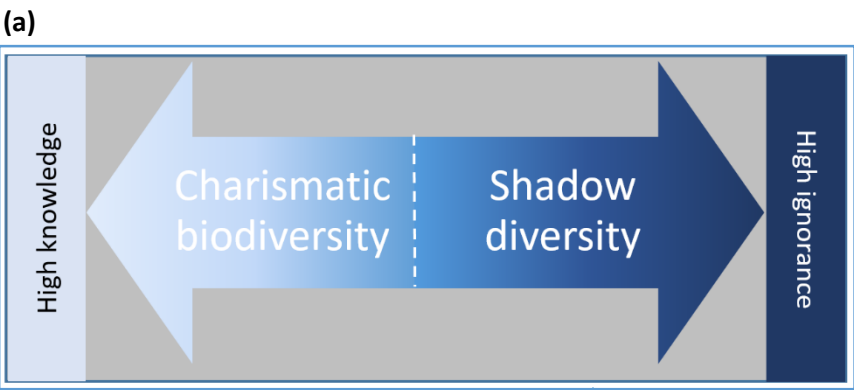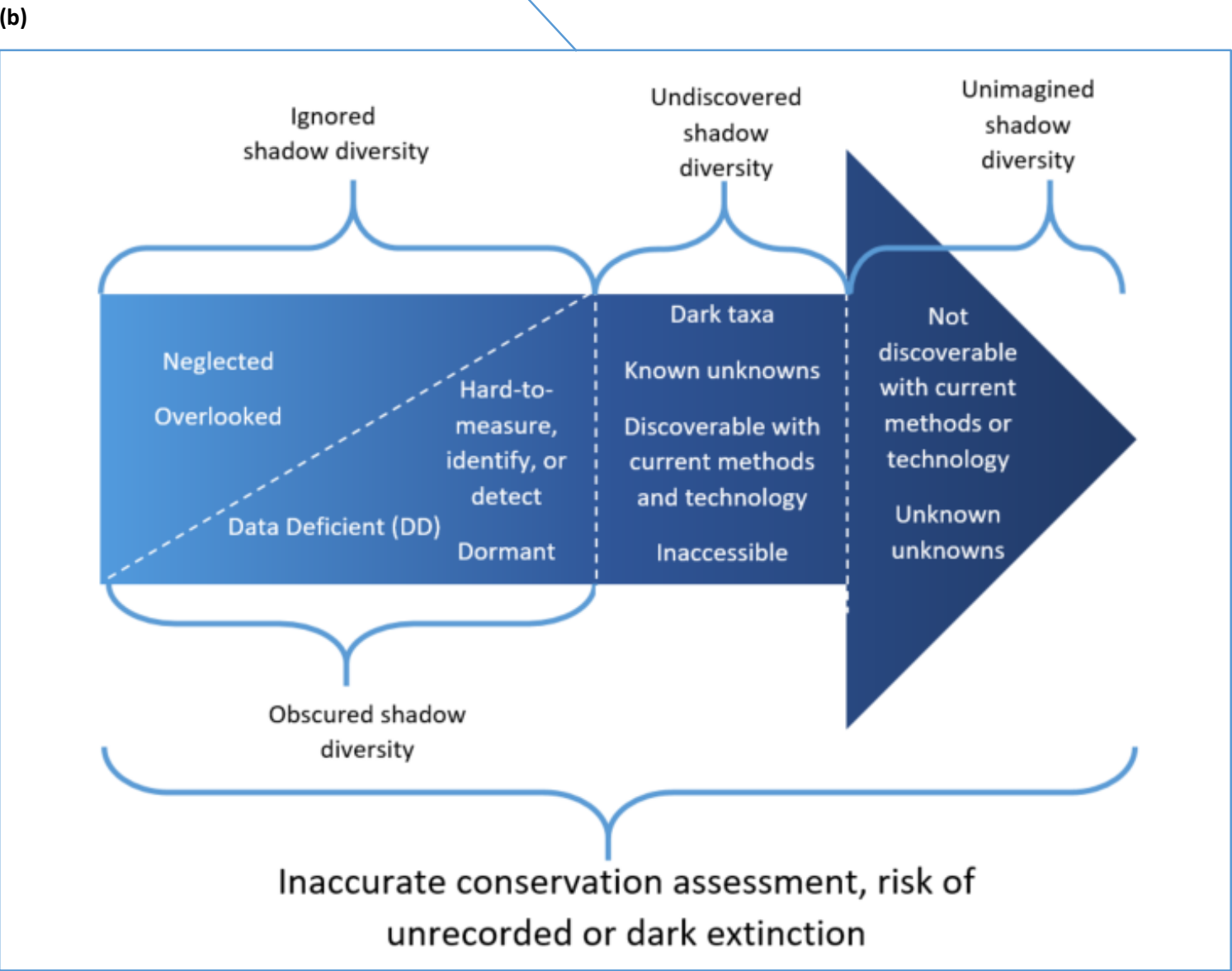

Supplement: Turton-Hughes et al. supplementary material 3 — Turton-Hughes et al. supplementary material [file S2755095824000214sup003.pdf]

Figure 4:

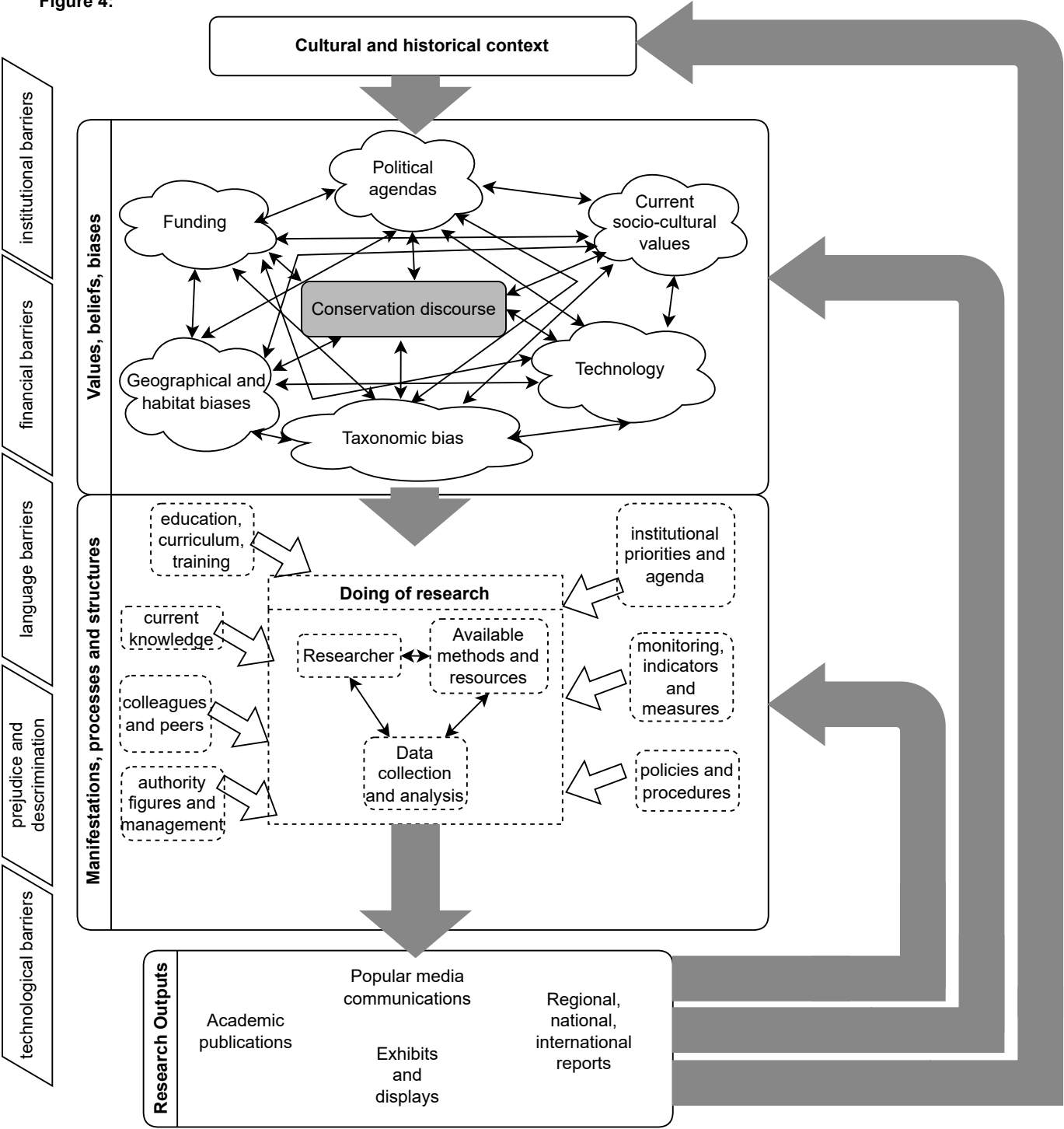

Supplement: Turton-Hughes et al. supplementary material 4 — Turton-Hughes et al. supplementary material [file S2755095824000214sup004.pdf]
