# Supplementary material for: The diversity of ignorance and the ignorance of diversity: origins and implications of “shadow diversity” for conservation biology and extinction
Source: Camb Prism Extinct. 2024 Nov 22;2:e18. doi: 10.1017/ext.2024.21 (PMC11895729; doi:10.1017/ext.2024.21)
Supplement: Turton-Hughes et al. supplementary material 5 — Turton-Hughes et al. supplementary material [file S2755095824000214sup005.pdf]

Shadow diversity: implications for conservation practice

Thinking tools and frameworks

As a conservation concept, shadow diversity enables improved handling of speculative or inaccessible ecosystems, such as previously speculative whale fall ecosystems which are now well-established (Bastian, 2020; Smith *et al.* 2019).

Moves away from biodiversity power relations where all species must be controlled and accounted for by the taxonomic system: potential to feed-into de-growth and de-colonial discourses.

Recognition of the transience of what we choose to conserve now - place on shadow diversity spectrum is not fixed.

Scope for the arts to explore unimagined sides of shadow diversity, perhaps shadow diversity (Figure 4) or its inspired artwork will generate new interpretations of “other worlds” (Carver 2023) which will in turn inspire those working in conservation.

Conservation Biology

Supports moving away from solely species-based conservation, bolstering support for habitat and ecosystem-level conservation.

For species-focused work, shadow diversity can offer a prompt to more consistently and explicitly acknowledge unrecorded biodiversity, using existing metrics.

Ability to distinguish between obscured, undiscovered, unimagined biodiversity, enables targeting specific challenges relevant to levels of ignorance, rather than homogenous ‘unknowns’.

Supports critical reflections on the origins of shadow diversity (Figure 4), encouraging historically-grounded and reflexive conservation practice and teaching.

Public understanding and support

A public term for shadow diversity means previously marginalised biodiversity becomes more widely discussed, leading to more interest, funding and eventually, taxonomists in their area.

Aids conceptualisation of the unknowns of extinction estimates in a manner which may appeal to individuals less comfortable with statistics.

Bringing unknown biodiversity to the public imagination may lead to increased appreciation for obscured biodiversity, in daily life and local areas. At an individual level, this could impact daily choices, such as composting and diet – with wider implications for health and social equity (Ishaq *et al. 2021,* Robinson *et al.* 2022) .

Aids public understanding and care of unseen biodiversity, thus supporting conservation behaviours in community and private spaces.

Policy and education

Policymakers can use shadow diversity to critique policy prior to implementation and ensure any taxonomic biases are minimized. An example of this would be England (UK)’s Biodiversity Net Gain (BNG) policy, arguably a missed opportunity for invertebrate conservation (Duffus *et al.* 2024).

Policymakers may use shadow diversity as part of justifying larger areas for conservation protection to account for uncertainties and the future potential of yet undiscovered life.

In education, shadow diversity supports a multidisicplinary approach to conservation, providing a way to engage with nature beyond identifying and counting species and including imagined life in local nature (such as in soils and under tree bark). Shadow diversity can support extending cross-curricular biodiversity content beyond charismatic macro-flora, fauna, and funga, and bringing ignored shadow diversity (such as microbes) beyond the biology classroom, helping to shift influence on future scientists, taxonomists, and wider society (Figure 4) .

The suggestions above are not intended to be followed prescriptively but serve to demonstrate how the language and framework of shadow diversity have the potential to instigate ripple effects in surprising directions. We consider a wide view of conservation practice including education, policymaking, and public engagement (see Figure 4).
