# Supplementary material for: The diversity of ignorance and the ignorance of diversity: origins and implications of “shadow diversity” for conservation biology and extinction
Source: Camb Prism Extinct. 2024 Nov 22;2:e18. doi: 10.1017/ext.2024.21 (PMC11895729; doi:10.1017/ext.2024.21)
Supplement: Turton-Hughes et al. supplementary material 6 — Turton-Hughes et al. supplementary material [file S2755095824000214sup006.pdf]

Supplementary Materials

Table S2: Extinction of unknown species review table:

Term	No.	Summary and Analysis	References
**Cryptic**	10	*A mix of uses for the term, including firstly, the extinction of a species that went unnoticed due to being uncharismatic as a species [1]; and extinct species, 'newly described' through close examination of morphology [2]. Secondly, the term refers to potential or suspected cryptic (often genetically analysed) extinctions that have already occurred [3]. Thirdly, two papers specific to the extinction risk for undescribed species of lizard (in two papers) and unspecified endophytes [4]. Finally, one paper had a paleontological focus [5]. All papers, except Schlegel et al., analyse one or more specific named species or guild that are extinct or suspected of extinction; very specific species guild. However, Schlegel's work examines endophytes for a particular tree species, so specificity is a key theme.*	[1]: (Fisher and Ineich 2012);<br>[2]: (Nakamura et al. 2013);<br>[3]: (Leonard et al. 2007; McKinney et al. 2012; Melzer et al. 2019; Scarsbrook et al. 2021);<br>[4]: (McDonald et al. 2022; Oliver et al. 2023; Schlegel et al. 2018);<br>[5]: (Cowman and Bellwood 2011)
**Crypto**	2	*One paper uses a similar definition and meaning to that of dark extinction, though a little less explicit regarding human-induced extinction, the authors mention human activities p. 73 [6]. The other paper uses crypto-extinction in the abstract, but does not mention or define it again. They use the term 'undescribed' much more frequently in the paper [7].*	[6]: (Giam et al. 2011)<br>[7]: (Kristensen et al. 2020)
**Dark**	2	*Clearly define dark extinction as species extinct prior to scientific discovery through human causes ('anthropogenic extinction') [8, 9]. The authors provide precise historical layers and timings set in the environmental history of the location of study [8] and make it clear most dark extinctions will never become specifically named species with specimens or physical remains.*	[8]: (Lambdon and Cronk 2020)<br>[9]: (Boehm and Cronk 2021)
**Hidden**	4	*In two cases, 'hidden extinction' seems to be a synonym for cryptic extinctions, where the loss of specific species was only revealed after later genetic analysis [10]. In two cases, the term was utilised by science communicator and a reporter, the term 'hidden' was not used in the original academic article, suggesting the recognition a more public-facing term would be beneficial [11].*	[10]: (Wu et al. 2022; Yan et al. 2018)<br>[11]: (Callaway 2020; Lawton 2023)
**Invisible**	2	*Both uses of this term come from more popular, non-scientific and non-high-academic sources. The first author [12] enjoys writing about the environment, referring to extinction crisis as invisible to most urban dwellers of Australia because they incorrectly assume wildlife areas hold the wildlife and don't see the losses. The second [13] is a short piece from non-ecology experts, drawing attention to unnoticed anticipated extinction and decline in insects and incorporating art/graphic design to make this message clearer.*	[12]: (Rawson 2018)<br>[13]: (Boggs and Siegrist 2020)
**Secret**	1	*The oldest paper for this topic from the Web of Science search (1993) [14]. A paper on genetic biodiversity lost in forests which looks at local population genetic diversity loss that goes unseen.*	[14]: (Ledig 1993)

**Undetected**	3	*The first paper using this term [15] presents a non-parametric model for calculating previously unknown extinctions; the second [16] notes likely extinctions of rare species already extinct in the area of their study. Finally, [17] provide a comparison of two models for calculating undetected extinctions, one model being [15], the other uses the label 'undescribed' [18]*	[15]: (Chisholm et al. 2016)<br>[16]: (Kaye et al. 2019)<br>[17]: (Lum et al. 2021)
**Undescribed**	1	*Full term used is 'undescribed species extinction' [18], though numerous variations and terms are used throughout the article, it seems no one term is chosen or defined. The first quantitative model for estimating theoretical past unknown extinctions.*	[18]: (Tedesco et al. 2014)
**Undocumented**	2	*Both focus on marine extinctions and seem to be pre-cursors to the notion of theoretical unknown extinctions outside of palaeontology: [19] explores the potential for undocumented extinctions in marine life [20] considers suspected previous and future undocumented extinction in marine sediment biodiversity.*	[19]: (Powles et al. 2000)<br>[20]: (Snelgrove 1998)
**Unknown**	2	*Both articles are from environmental humanities scholars and focus on conceptualisation and engaging with absence of unknown species [21].*	[21]: (Bastian 2020; van Dooren 2022)
**Unrecognised**	2	*Both quite different to previous search results: [22] examined the potential extinction of specific ancestral silk genes (a type of spider's silk gene paralog), in this sense, it was most similar in topic to [14]. [23] reanalysed elements of fossil records leading to 'new' extinctions, previously unrecognised. No anthropological factors were noted, more focused on sea level changes due to non-human-induced climate conditions.*	[22]: (Ayoub and Hayashi 2008)<br>[23]: (Pimiento et al. 2017)
**Unrecorded**	3	*Early discussion of theoretical potential for unrecorded extinctions to mean hypothetical extinctions that we cannot know what they were but can be confident species are already extinct due to anthropogenic factors such as deforestation [24]. [25] mentions unrecorded extinctions in a similar meaning to dark extinctions, but the focus is on rats as the cause for these unknown extinctions. [26] sees overlaps with cryptic and hidden extinctions, using the term to mean unaware of previous extinctions (of plants in this case).*	[24] (Cardoso et al. 2010)<br>[25] (Harper and Bunbury 2015)<br>[26] (Vorontsova et al. 2021)
Notes on 'dark extinction' literature search: *All search results from Web of Science. Search results that returned articles which used the terms but not similar meanings were removed from results (for example, 'unknown extinctions' included 6 publications relating to beyond-earth extinctions, such as the extinction of stars, and 3 'false hits', where the words 'unknown' and 'extinction' were found together, but had been taken out of context, the articles were focused on other elements of extinction, such as unknown extinction risks for pandas as their conservation status was downgraded). Where American spelling variations existed, both British and American search terms were used ("unrecognised extinction$" OR "unrecognized extinction$").*			

List of References for Table S2

Ayoub NA and Hayashi CY (2008) Multiple Recombining Loci Encode MaSp1, the Primary Constituent of Dragline Silk, in Widow Spiders (Latrodectus: Theridiidae). *Molecular Biology and Evolution*, **25**(2), 277–286. doi:10.1093/molbev/msm246.

Bastian M (2020) Whale Falls, Suspended Ground, and Extinctions Never Known. *Environmental Humanities*, **12**(2), 454–474. doi:10.1215/22011919-8623219.

Boehm MMA and Cronk QCB (2021) Dark extinction: the problem of unknown historical extinctions. *Biology Letters*, **17**(3), 20210007. doi:10.1098/rsbl.2021.0007.

Boggs J and Siegrist H (2020) Invisible Extinction. *Virginia Quarterly Review*, **96**(4), 16–17.
Callaway E (2020) Supercomputer scours fossil record for Earth's hidden extinctions. *Nature*, **577**(7791), 458–459. doi:10.1038/d41586-020-00117-1.
Cardoso P, Arnedo MA, Triantis KA and Borges PAV (2010) Drivers of diversity in Macaronesian spiders and the role of species extinctions. *Journal of Biogeography*, **37**(6), 1034–1046. doi:10.1111/j.1365-2699.2009.02264.x.
Chisholm RA, Giam X, Sadanandan KR, Fung T and Rheindt FE (2016) A robust nonparametric method for quantifying undetected extinctions. *Conservation Biology*, **30**(3), 610–617. doi:10.1111/cobi.12640.
Cowman PF and Bellwood DR (2011) Coral reefs as drivers of cladogenesis: expanding coral reefs, cryptic extinction events, and the development of biodiversity hotspots. *Journal of Evolutionary Biology*, **24**(12), 2543–2562. doi:10.1111/j.1420-9101.2011.02391.x.
Fisher R and Ineich I (2012) Cryptic extinction of a common Pacific lizard Emoia impar (Squamata, Scincidae) from the Hawaiian Islands. *Oryx*, **46**(2), 187–195. doi:10.1017/S0030605310001778.
Giam X, Scheffers BR, Sodhi NS, Wilcove DS, Ceballos G and Ehrlich PR (2011) Reservoirs of richness: least disturbed tropical forests are centres of undescribed species diversity. *Proceedings of the Royal Society B: Biological Sciences*, **279**(1726), 67–76. doi:10.1098/rspb.2011.0433.
Harper GA and Bunbury N (2015) Invasive rats on tropical islands: Their population biology and impacts on native species. *Global Ecology and Conservation*, **3**, 607–627. doi:10.1016/j.gecco.2015.02.010.
Kaye TN, Bahm MA, Thorpe AS, Gray EC, Pfingsten I and Waddell C (2019) Population extinctions driven by climate change, population size, and time since observation may make rare species databases inaccurate. *PLoS One*, **14**(10), e0210378. doi:10.1371/journal.pone.0210378.
Kristensen NP, Seah WW, Chong KY, Yeoh YS, Fung T, Berman LM, Tan HZ and Chisholm RA (2020) Extinction rate of discovered and undiscovered plants in Singapore. *Conservation Biology*, **34**(5), 1229–1240. doi:10.1111/cobi.13499.
Lambdon P and Cronk Q (2020) Extinction Dynamics Under Extreme Conservation Threat: The Flora of St Helena. *Frontiers in Ecology and Evolution*, **8**. doi:10.3389/fevo.2020.00041.
Lawton G (2023) The hidden extinction. *New Scientist*, **258**(3434), 46–49. doi:10.1016/S0262-4079(23)00681-4.
Ledig F (1993) Secret Extinctions - the Loss of Genetic Diversity in Forest Ecosystems. In M. A. Fenger, E. H. Miller, J. A. Johnson, and E. J. R. Williams, (eds), *Our Living Legacy: Proceedings of a Symposium on Biological Diversity*, Copenhagen V: Royal Danish Academy Sciences & Letters, 127–140.
Leonard JA, Vilà C, Fox-Dobbs K, Koch PL, Wayne RK and Van Valkenburgh B (2007) Megafaunal extinctions and the disappearance of a specialized wolf ecomorph. *Current Biology: CB*, **17**(13), 1146–1150. doi:10.1016/j.cub.2007.05.072.
Lum D, Tedesco PA, Hugueny B, Giam X and Chisholm RA (2021) Quantifying the relative performance of two undetected-extinction models. *Conservation Biology*, **35**(1), 239–248. doi:10.1111/cobi.13562.
McDonald PJ, Brown RM, Kraus F, Bowles P, Arifin U, Eliades SJ, Fisher RN, Gaulke M, Grismer LL, Ineich I, Karin BR, Meneses CG, Richards SJ, Sanguila MB, Siler CD and Oliver, PM (2022) Cryptic extinction risk in a western Pacific lizard radiation. *Biodiversity and Conservation*, **31**(8), 2045–2062. doi:10.1007/s10531-022-02412-x.

McKinney LV, Thomsen IM, Kjær ED, Bengtsson SBK and Nielsen LR (2012) Rapid invasion by an aggressive pathogenic fungus (Hymenoscyphus pseudoalbidus) replaces a native decomposer (Hymenoscyphus albidus): a case of local cryptic extinction? *Fungal Ecology*, **5**(6), 663–669. doi:10.1016/j.funeco.2012.05.004.

Melzer S, Hitchmough RA, Bell T, Chapple DG and Patterson GB (2019) Lost and Found: Taxonomic revision of the speckled skink (Oligosoma infrapunctatum; Reptilia; Scincidae) species complex from New Zealand reveals a potential cryptic extinction, resurrection of two species, and description of three new species. *Zootaxa*, **4623**(3), 441–484. doi:10.11646/zootaxa.4623.3.2.

Nakamura Y, Takahashi A and Ota H (2013) Recent cryptic extinction of squamate reptiles on Yoronjima Island of the Ryukyu Archipelago, Japan, inferred from garbage dump remains. *Acta Herpetologica*, **8**(1), 19–34. doi:10.13128/Acta_Herpetol-11924.

Oliver PM, Iskandar DT, and Richards SJ (2023) A new species of torrent-breeding treefrog (Pelodryadidae: Litoria) from the mountains of Papua, Indonesia, with new records and observations of Litoria dorsivena (Tyler, 1968). *Vertebrate Zoology*, **73**, 127–139. doi:10.3897/vz.73.e91111.

Pimiento C, Griffin JN, Clements CF, Silvestro D, Varela S, Uhen MD and Jaramillo C (2017) The Pliocene marine megafauna extinction and its impact on functional diversity. *Nature Ecology & Evolution*, **1**(8), 1100–1106. doi:10.1038/s41559-017-0223-6.

Powles H, Bradford MJ, Bradford RG, Doubleday WG, Innes S and Levings CD (2000) Assessing and protecting endangered marine species. *ICES Journal of Marine Science*, **57**(3), 669–676. doi:10.1006/jmsc.2000.0711.

Rawson J (2018) The invisible extinctions. *Meanjin*, **77**(3), 54–62. doi:10.3316/informit.984838453421962.

Scarsbrook L, Sherratt E, Hitchmough RA, and Rawlence NJ (2021) Skeletal variation in extant species enables systematic identification of New Zealand's large, subfossil diplodactylids. *BMC Ecology and Evolution*, **21**(1), 67. doi:10.1186/s12862-021-01808-7.

Schlegel M, Queloz V, and Sieber TN (2018) The Endophytic Mycobiome of European Ash and Sycamore Maple Leaves – Geographic Patterns, Host Specificity and Influence of Ash Dieback. *Frontiers in Microbiology*, **9**. Retrieved from https://www.frontiersin.org/articles/10.3389/fmicb.2018.02345

Snelgrove PVR (1998) The biodiversity of macrofaunal organisms in marine sediments. *Biodiversity & Conservation*, **7**(9), 1123–1132. doi:10.1023/A:1008867313340.

Tedesco PA, Bigorne R, Bogan AE, Giam X, Jézéquel C and Hugueny B (2014) Estimating How Many Undescribed Species Have Gone Extinct. *Conservation Biology*, **28**(5), 1360–1370. doi:10.1111/cobi.12285.

van Dooren T (2022) In Search of Lost Snails: Storying Unknown Extinctions. *Environmental Humanities*, **14**(1), 89–109. doi:10.1215/22011919-9481451.

Vorontsova MS, Lowry PP, Andriambololonera SR, Wilme L, Rasolohery A, Govaerts R, Ficinski SZ and Humphreys AM (2021) Inequality in plant diversity knowledge and unrecorded plant extinctions: An example from the grasses of Madagascar. *Plants People Planet*, **3**(1), 45–60. doi:10.1002/ppp3.10123.

Wu MY, Lau CJ, Ng EYX, Baveja P, Gwee CY, Sadanandan K, Ferasyi TR, Haminuddin, Ramadhan R, Menner JK and Rheindt FE (2022) Genomes From Historic DNA Unveil Massive Hidden Extinction and Terminal Endangerment in a Tropical Asian Songbird Radiation. *Molecular Biology and Evolution*, **39**(9), msac189. doi:10.1093/molbev/msac189.

Yan F, Lü J, Zhang B, Yuan Z, Zhao H, Huang S, Wei G, Mi X, Zou D, Xu W, Chen S, Wang J, Xie F, Wu M, Xiao H, Liang Z, Jin J, Wu S, Xu C, Tapley B, Turvey ST, Papenfuss TJ, Cunningham AA, Murphy RW, Zhang Y and Che J (2018) The Chinese giant salamander exemplifies the hidden extinction of cryptic species. *Current Biology*, **28**(10), R590–R592. doi:10.1016/j.cub.2018.04.004.

Figure S6: A bar chart showing twenty-one labels found in literature relating to shadow diversity language and the total number of publications found in the Web of Science data base for each term under a ‘topic’ search. The highest bars are for cryptic species and taxa, undescribed species and taxa, and unknown species and taxa.

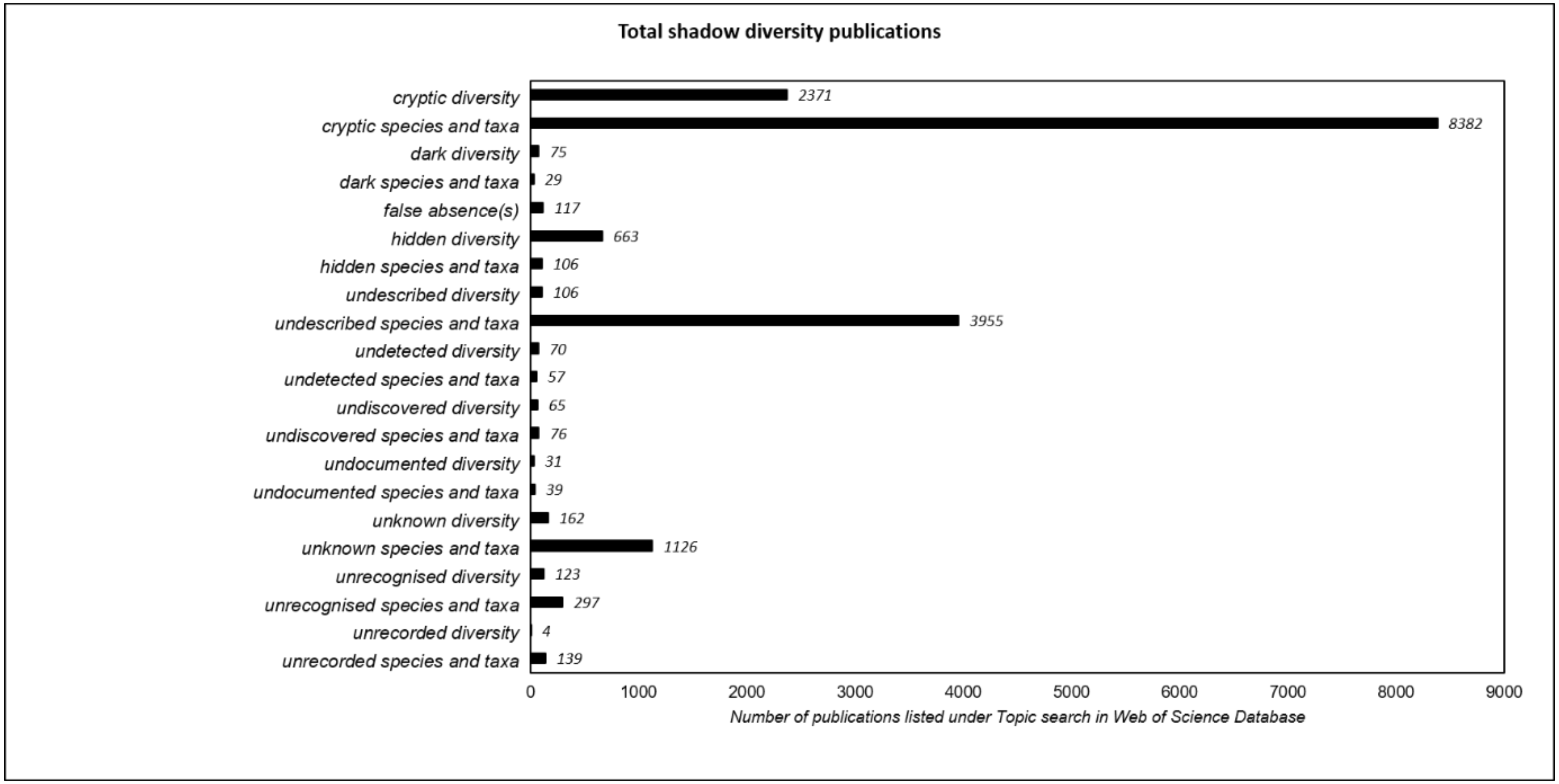

Table S3: Shadow diversity terminology review table

Term	Previous studies	References
**Cryptic**	• *Cryptic species are frequently defined as multiple species previously thought to be one species, with differentiating characteristics shown via genetic sequencing, chemical tests, or other non-morphological techniques such as auditory differences [1]. Most studies using the term "cryptic" use phylogenetic or molecular DNA or RNA-based techniques [2]. Others use cryptic to mean obscured, such as underground species or well-camouflaged [3]. A previous review of cryptic species literature noted the confusion in defining the term and range of meanings it had already been used for by 2007 [1] and pleas for consistent accurate use of language for this concept continue [4].*<br>• *Overall, literature regarding cryptic focus on speciation and species boundaries [5]; estimated numbers of cryptic species [6]; and novel methods of discovery, such as near-infrared spectroscopy [7] to support species differentiation; DNA barcoding [8]; or integrated approaches using eDNA and morphology or traditional identification approaches [9].*<br>• *Several studies note the importance of incorporating cryptic species into ecological and conservation models and approaches [10].*	[1] (Bickford et al. 2007)<br>Examples: (Grabowski et al., 2017; Morinière et al., 2019; Sharkey et al., 2021)<br>[2] (Delić et al. 2017; Grabowski et al. 2017; Hebert et al. 2004; Jofre et al. 2022; Marko and Moran 2009)<br>[3] (Claridge et al. 2004)<br>[4] (Janosik and Halanych 2010)<br>[5] (De Clerck et al. 2013; Jorna et al. 2021)<br>[6] (Baker and Bradley 2006; McDonald et al. 2022)<br>[7] (Klarica et al. 2011)<br>[8] (DeSalle and Goldstein 2019; Hebert et al. 2004)<br>[9] (Mátis et al. 2022; Moraes et al. 2021)<br>[10] (Delić et al. 2017; Fišer et al. 2018)
**Dark**	• *Dark diversity is an estimated measure for absent species that ecologically would be expected to inhabit an area, but do not [11]. Dark diversity is therefore a set term with accompanying methods. The purpose of calculating dark diversity, is the aim to measure actual, rather than false, absences. Up to the 2023, we found 70 papers using the term 'dark diversity' as per this definition and method [12]. We concluded the term is not suitable for describing anticipated dark extinctions without adding to confusing and contradictory definitions.*<br>• *All 'dark species' search results used the term 'dark' in the context of describing and comparing species, for example, darkly-coloured creatures [13], though some studies included finding new species [14], in all cases, the term 'dark' was referring to colour or shade in the context of those articles, therefore only dark taxa and dark taxon were included.*<br>• *'Dark taxa' refers to species for which we have some genetic material, often from genetic sequencing, but no matching described organism in metabarcoding databases, because the species is undiscovered, or unnamed, or because we do not yet have confirmed genetic sequences in the database for the species we have described traditionally [15].*	[11] (Pärtel *et al.* 2011)<br>[12] Examples include: (Boussarie *et al.* 2018; de Bello *et al.* 2016; Fernandes *et al.* 2019; Fløjgaard *et al.* 2020; Ronk *et al.* 2015; Valdez *et al.* 2021)<br>[13] Examples (Damm *et al.* 2010; Ito *et al.* 2015; Johnson and Rutowski 2022; Nupponen and Junnilainen 1998; Phinney *et al.* 2021; Roth *et al.* 2021; Strong *et al.* 1991)<br>[14] (Balboa *et al.* 2017; Hedges and Garrido 1992; Huber *et al.* 2019; Kasparek 2019; Mejdalani and Cavichioli 2014)<br>[15] (Kortmann *et al.* 2022; Morinière *et al.* 2019; Nilsson *et al.* 2019; Page 2011, 2016; Ryberg and Nilsson 2018)

	• *Biological dark matter, or microbial dark matter and viral dark matter [16]: refer to understudied biological particles and substances, often evading visible detection, and previously thought not to be consequential for the biological functioning of a creature or cell.*	[16] (Bernard *et al.* 2018; Bruno *et al.* 2017; Ross 2016; Zamkovaya *et al.* 2021)
**False absences**	• *Almost all papers encountered referred to the importance of accounting for imperfect detection and false absences for conservation purposes and accurate data to inform conservation decisions [17].*<br>• *Most papers deal with various forms of occupancy modelling, however these models are focused on minimising or eliminating false absences of known species, often already a subject of conservation [18]*<br>• *Models centre on calculating detection probabilities of specific species (see list above), method [19], or area, for purposes of calculating minimum survey efforts [20] and to account for false absences [21], providing statistical methods to estimate these [22].*<br>• *Newest models allow for latest methods of data collection, such as remote data rather than only traditional physical survey visits [23].*	[17] (Ariefiandy et al. 2014; Benoit et al. 2018; Issaris et al. 2012)<br>[18] (Bingham et al. 2009; Fusco-Costa et al. 2023; Hanspach et al. 2011; Lauriault and Wiersma 2019; Williams and Berkson 2004)<br>[19] (Wedderburn 2018)<br>[20] (Garrard et al. 2015; Wintle et al. 2005)<br>[21] (Riva et al. 2020)<br>[22] (Wilson et al. 2011; Wintle et al. 2005)<br>[23] (Mohankumar and Hefley 2022; Rhinehart et al. 2022)
**Hidden**	*Mixed use:*<br>• *This can be speciation, often used interchangeably with 'cryptic species' [24] to refer to new discoveries previously 'hidden' within a genus, often revealed via DNA or new methodologies, or anticipated future cryptic species still in hiding.*<br>• *Practical nature of false absences [25]*<br>• *Similar to 'unknown' species, focus on habitats or microhabitats previously under-researched, such as a plant rhizosphere [26]. The focus is usually on one genus or taxonomic group per study.*<br>• *Hidden diversity has a similar application, but is additionally used to refer to estimates of further anticipated novel species, such as in [27].*<br>• *Some cultural 'hidden diversity', such as [28], referring to multiple species by one local, vernacular term. In this sense, it resembles a cultural version of cryptic diversity.*	[24] (Milić et al. 2019; Stork 2018)<br>[25] (Birks et al. 2016; Pärtel 2014)<br>[26] (Bass et al. 2018)<br>[27] (Kynčlová et al. 2010)<br>[28] (Cavalcanti and Albuquerque 2013)
**Invisible**	• *Invisible diversity is seldom utilised in ecology: Of the two papers on "invisible diversity", only one is widely available: This examines co-extinction of parasites, a focus of study neglected by ecologists according to Votýpka and colleagues [29].*<br>• *Beyond ecology 'invisible diversity' is more commonly used to discuss and study human equality diversity and refers to invisible traits and characteristics within this [30]. This would make it a confusing term to adopt for quite different purposes in biological setting.*<br>• *Invisible species and taxa is used infrequently. The main use seems to relate to molecular level species, which are quite literally, invisible, within biophysics and*	[29] (Votýpka et al. 2020)<br>[30] (Blank 2005; Peters 2021; Popova 2018)<br>[31] (Hansel et al. 2015; Ross 2016)<br>[32] (Hurrell and Puentes 2017; Puentes et al. 2019)

	biochemistry. The exception to this are two recent articles who utilise 'invisibility' regarding invisible, biocultural knowledge of plants, which owes its invisibility to use of different cultures Chinese immigrants to Argentina and commercial invisibility of some taxa [32].<br>• *Overall, the term 'invisible' seems to be used either to denote literal invisibility at a molecular level, or in the context of social and cultural invisibilities. With less than ten papers (six in total) referring to invisible diversity or species/taxa, this term was excluded from Figure 4.*	
**Neglected**	• *'Neglected' is used to highlight previous lack of attention towards biodiversity of specific regions [33], groups of species [34], or specific species for taxonomic revision [35]. Though even where the neglect cited is for a wide geographical range [33], the study realistically focuses on just one or two hitherto cryptic species. In this sense, 'neglected' diversity is being used as a synonym for overlooked areas of biodiversity [36], in practice, most often looking at specifically previously overlooked species.*<br>• *Around half of the search results for neglected species related to plant science [36], suggesting 'neglected' is a term particularly favoured by plant taxonomists or commonly used within that taxonomic specialism.*	[33] (Caković et al. 2018)<br>[34] (Durigon et al. 2019; Gorbalenya and Siddell 2021; Lehnert et al. 2009; Lopes et al. 2021; Lopez-Vaamonde et al. 2019; Reumont and Marcus 2018; Wanninger 2008)<br>[35] (Bakalin et al. 2019; Luna et al. 2021)<br>[36] (Wyckhuys et al. 2021)<br>[37] (Martínez-Azorín et al. 2018; Uotila 1979)
**Overlooked**	• *Heavily dominated by studies focused on less popular species [38], uncharismatic species [39] and those difficult to detect [40].*<br>• *Clear crossover of concept with the label 'neglected' as a synonym for this label, with many of the same patterns, including a high number of plant science-based papers [41] (with the topic 'plant science' making up around 40% of results, compared to approximately 30% listed under zoology, etymology and marine freshwater topics combined).*	[38] (Bronne et al. 2022)<br>[39] (Boom 2021)<br>[40] (Mannerkoski 2000; Sampaio et al. 2010)<br>[41] (Lumbsch et al. 2003; Maldonado et al. 2017; Metusala 2019)
**Undescribed**	• *We estimate around half of articles focus on the discovery of novel species and use 'undescribed' as a linguistic tool to emphasise the taxonomic novelty and significance of the new descriptions: we eliminated over 45% (1,750 of 3,955 articles) of search results for 'undescribed species' by excluding entries preceded by 'hitherto-', 'previously-' and excluding entries encased by a number and 'of' a particular species. We still saw numerous entries describing new species even after these exclusions. Examples of newly discovered species using this term in their title, abstract or keywords: [42].*<br>• *Of newly described species, some used traditional methods such as morphology or culturing [43], most frequent was the sole or combined use of DNA sequencing, such as use of mitochondrial DNA and morphological features for the snail genus Lithasa [44], molecular phylogenetics used to distinguish deep-*	[42] (Doucette 2012; Dumont and Batra 1978; Long 1916; van der Linde et al. 2022)<br>[43] (Catania and Romero 2005)<br>[44] (Minton and Lydeard 2003)<br>[45] (Horton et al. 2020)<br>[46] (Belluardo et al. 2022)<br>[47] (Decker 2016; Ebejer and Barták 2019; Fujikura et al. 2010; García-Avendaño and Guerrero 2018; Hawksworth 2004; Stork et al. 2008; Timm 1998)<br>[48] (Smith and Heard 2001)<br>[49] (De Laubenfels and Adema 1998; Nuryadi et al. 2020; Orange 2004)

	sea amphipods [45], and Madagascan ‘narrow-mouthed frogs of the genus Rhombophryne’ [46].<br>• *Beyond describing new species, a large portion of articles used ‘undescribed’ to differentiate between an organism that had been found, collected, or in some was discovered, but not yet formally described [47]. In some cases, this use of the term ‘undescribed’ became mixed with the now newly described [48]. Others used this differentiation in tandem with calls for further studies required [49], or to alert their taxonomic peers to newly described species as well as likely future undescribed diversity [50].*<br>• *A smaller subset of articles focused on where or how much undescribed remains. These articles provided macro-level analysis, utilising large-scale datasets; statistical estimates or modelling, and often at global scales. They include undescribed mammals [51], fungi [52], amphibians and land mammals [53].*<br>• *One article looked at dark extinction estimates, again at global level using statistical modelling [54].*	[50] (Orihara and Smith 2017)<br>[51] (Fisher et al. 2018)<br>[52] (Hawksworth and Rossman 1997)<br>[53] (Giam et al. 2011)<br>[54] (Tedesco et al. 2014)
**Undetected**	• *High numbers of papers use this term to refer to statistical models used to estimate numbers of species not captured by sampling efforts [55] and especially dominated by Chao’s work [56]. In this sense, we see high crossover with ‘false absences’, see above.*<br>• *Still some studies focussed on particular species that have gone unnoticed [57], new species taxonomically delimited via phylogenetic methods [58] and use of new methods to find species [59] and predictions for where further undetected species might yet be found [60].*<br>• *Finally, though looking at local extinction, we also find a further dark extinction statistical estimate for butterflies in Singapore [61].*	[55] (Gotelli et al. 2010; Iknayan et al. 2014; Mao and Colwell 2005; Montgomery et al. 2021; Yamaura et al. 2011)<br>[56] (Chao et al. 2015, 2017a, 2017b; Chao and Shen 2004; Huber and Chao 2019)<br>[57] (Berger 2008; Madani 2021; Slapcinsky and Lasley 2007)<br>[58] (Lavin et al. 2018)<br>[59] (Webster et al. 2022)<br>[60] (Hohlfeld and Arndt 2022; Hsieh and Linsenmair 2011)<br>[61] (Theng et al. 2020)
**Undiscovered**	• *Focus on patterns of, discovery and predictions for locating undiscovered species, often on macro-scale quantitative studies [62] or how many species of a kingdom are yet to be discovered [63]. The term is strongly linked to species discovery curves and models using this [64].*<br>• *A small number of papers describe novel species [65]: similar to ‘undescribed’, see above. However a larger number use the term to refer to the continued need to study a particular geographical or taxonomic area due to high likelihood of yet undiscovered species [66] and the concerns for conservation implications [67] as a result.*	[62] (Costello et al. 2015; Fisher et al. 2018; Giam et al. 2010; Hawksworth and Rossman 1997; Joppa et al. 2011; Lu et al. 2021; Lücking 2012; Moura and Jetz 2021)<br>[63] (Stork et al. 2008)<br>[64] (Solow and Smith 2005)<br>[65] (Almeida et al. 2012; Godeas and Arambarri 1996; Liu et al. 2022)<br>[66] (Fernandes et al. 2015)

		[67] (Ota et al. 2015; Payo-Payo and Lobo 2016)
**Unknown**	• *Focus on discoveries of new species [68]*<br>• *Focus on habitats which have led to the 'discovery' of multiple new species. [69]*<br>• *Methodology often utilises DNA sequencing [70].*<br>• *Less frequent under this label, but still some quantitative estimates of unknown biodiversity [71]*	[68] (Agudelo-Zamora et al. 2020; Kumar et al. 2017; Schuster and Machado 2021)<br>[69] (Schmidt et al. 2015)<br>[70] (Bråte et al. 2010; Vieira et al. 2019)<br>[71] (Gustafsson et al. 2019)
**Unrecognised**	• *Commonly used as a synonym or alternative term to 'cryptic diversity' [72] and helping to clarify species boundaries where confusion existed [73].*<br>• *Some overlap with hidden species [74].*<br>• *At times used to mean previously overlooked taxa, such as in the case of investigating orthoreoviruses in North American bats (Feng et al. 2022), or the gut parasites of small crustaceans, copepods [75]. Janosik and Halanych (2010) highlight this linguistic distinction, stating 'we argue that there is a need for using more precise scientific language when discussing whether lineages of distinct species are difficult to detect (i.e., cryptic) or merely have not been noticed (i.e., unrecognized)[76]. Despite their plea, however, cryptic and unrecognised species are often used somewhat interchangeably, as noted above.*<br>• *Majority of articles focus on newly discovered species, as per references above and almost exclusively utilise DNA analysis to support the recognition of previously unrecognised species [77]. The exception to this being papers published prior to genetic sequencing, in this case dealing with disagreements over the 'correct' placement of species within the taxonomic system [78].*<br>• *As with cryptic diversity, the vast majority of studies are hyper-specific to a particular sub-set of species or sub-genus [79], either from a defined, local area [80], or reviewing the taxonomy of a specific area of phylogenetics or taxonomy. These hyper-precise micro studies are reflected in that the most frequent publications are Zootaxa and Zookeys, followed largely by an array of specialist publications such as Mycotaxon and Canadian Entomologist. This is indicative that many of these studies deal with finer points of precise, niche, specialist taxonomy.*	[72] (Baker and Bradley 2006; Francis et al. 2010; Jofre et al. 2022; Mátis et al. 2022; Muis et al. 2023; Pereira and Prado 2022; Říčan et al. 2022; Rowley et al. 2015; Schilthuizen et al. 2022; Stefan et al. 2018; Wright et al. 2022)<br>[73] (PAcheco et al. 2020)<br>[74] (Brownstein et al. 2022; Packer and Taylor 1997)<br>[75] (Skovgaard et al. 2012)<br>[76] (Janosik and Halanych 2010)<br>[77] (Akob et al. 2022; Caldwell and Chatterton 1995; Craig and Felder 2022; Eitner 1995; Huang and Shen 2016; Oberprieler et al. 2018; Prihatini et al. 2016; Saha et al. 2022)<br>[78] (Welden 1958)<br>[79] (Marko and Moran 2009)<br>[80] (López et al. 2007)
**Unrecorded**	• *We see a high crossover with 'unrecognized', 'unknown' and 'undescribed' for the term unrecorded: this includes 'hitherto' unrecorded species to highlight novelty [81]*<br>• *Unlike labels 'overlooked' and 'neglected', which had higher use for studies on plants, we found the highest number of unrecorded species publications were categorized under 'biodiversity conservation' and within this, around two thirds of publications focused on Korean and South Korean new species [82], which may*	[81] (del Carmen H. Rodríguez et al. 2021; Miranda 2017)<br>[82] (Choi et al. 2020; Humala et al. 2022; Kim et al. 2012, 2020; Roh et al. 2016)

	suggest particular cultural or linguistic preferences (or translation practicalities) for using one term in English over another.
**Other**	Other terms searched for with ten or fewer relevant results: **mystery** (0 (bio)diversity, 1 species, 0 taxa); **concealed** (3 for (bio)diversity, 7 for species, 0 for taxa); **unnoticed** (4 (bio)diversity, 4 for species, 1 for taxa); **secret** (1 for (bio)diversity, 1 for species 0 for taxa).
All terms were searched for using the Web of Science database "topic" search, for species, taxa, taxon, diversity and biodiversity, with US spelling variations included where appropriate (such as 'unrecognized' and 'unrecognised'), time period from earliest available in Web of Science, to end of 2022.	

List of References for Table S3

Agudelo-Zamora HD, Tavera J, Murillo YD and Ortega-Lara A (2020) The unknown diversity of the genus Characidium (Characiformes: Crenuchidae) in the Chocó biogeographic region, Colombian Andes: Two new species supported by morphological and molecular data. *Journal of Fish Biology*, **97**(6), 1662–1675. doi:10.1111/jfb.14527.

Akob DM, Sutton JM, Bushman TJ, Baesman SM, Klein E, Shrestha Y, Andrews R, Fierst JL, Kolton M, Gushgari-Doyle S, Oremland RS and Freeman, JL (2022) Acetylenotrophic and Diazotrophic Bradyrhizobium sp. Strain I71 from TCE-Contaminated Soils. *Applied and Environmental Microbiology*, **88**(22), e01219-22. doi:10.1128/aem.01219-22.

Almeida AL, Kishimoto RG and Carbayo F (2012) Two new land planarian species (Platyhelminthes: Tricladida) from the Brazilian Atlantic Forest. *Zoologia (Curitiba)*, **29**, 430–438. doi:10.1590/S1984-46702012000500006.

Ariefiandy A, Purwandana D, Seno A, Chrismiawati M, Ciofi C, and Jessop TS (2014) Evaluation of three field monitoring-density estimation protocols and their relevance to Komodo dragon conservation. *Biodiversity and Conservation*, **23**(10), 2473–2490. doi:10.1007/s10531-014-0733-3.

Bakalin VA, Fedosov VE, Fedorova AV and Nguyen VS (2019) Integrative Taxonomic Revision of Marsupella (Gymnomitriaceae, Hepaticae) Reveals Neglected Diversity in Pacific Asia. *Cryptogamie, Bryologie*, **40**(7), 59–85. doi:10.5252/cryptogamie-bryologie2019v40a7.

Baker RJ and Bradley RD (2006) Speciation in Mammals and the Genetic Species Concept. *Journal of Mammalogy*, **87**(4), 643–662. doi:10.1644/06-MAMM-F-038R2.1.

Balboa C, Hinojosa-Díaz I and Ayala R (2017) New dark species of the bee genus Colletes (Hymenoptera, Colletidae) from Mexico and Guatemala. *Zootaxa*, **4320**(3). doi:10.11646/zootaxa.4320.3.1.

Bass D, van der Gast C, Thomson S, Neuhauser S, Hilton S and Bending GD (2018) Plant Rhizosphere Selection of Plasmodiophorid Lineages from Bulk Soil: The Importance of "Hidden" Diversity. *Frontiers in Microbiology*, **9**, 168. doi:10.3389/fmicb.2018.00168.

Belluardo F, Scherz MD, Santos B, Andreone F, Antonelli A, Glaw F, Munoz-Pajares AJ, Randrianirina JE, Raselimanana AP, Vences M and Crottini A (2022) Molecular taxonomic identification and species-level phylogeny of the narrow-mouthed frogs of the genus Rhombophryne (Anura: Microhylidae: Cophylinae) from Madagascar. *SYSTEMATICS AND BIODIVERSITY*, **20**(1), 1–13. doi:10.1080/14772000.2022.2039320.

Benoit D, Jackson DA and Ridgway MS (2018) Assessing the impacts of imperfect detection on estimates of diversity and community structure through multispecies occupancy modeling. *Ecology and Evolution*, **8**(9), 4676–4684. doi:10.1002/ece3.4023.

Berger J (2008) Undetected species losses, food webs, and ecological baselines: a cautionary tale from the Greater Yellowstone Ecosystem, USA. *Oryx*, **42**(1), 139–142. doi:10.1017/S0030605308001051.

Bernard G, Pathmanathan JS, Lannes R, Lopez P and Bapteste E (2018) Microbial Dark Matter Investigations: How Microbial Studies Transform Biological Knowledge and Empirically Sketch a Logic of Scientific Discovery. *Genome Biology and Evolution*, **10**(3), 707–715. doi:10.1093/gbe/evy031.

Bickford D, Lohman DJ, Sodhi NS, Ng PKL, Meier R, Winker K, Ingram KK and Das I (2007) Cryptic species as a window on diversity and conservation. *Trends in Ecology & Evolution*, **22**(3), 148–155. doi:10.1016/j.tree.2006.11.004.

Bingham E, Kabán A and Fortelius M (2009) The aspect Bernoulli model: multiple causes of presences and absences. *Pattern Analysis and Applications*, **12**(1), 55–78. doi:10.1007/s10044-007-0096-4.

Birks HJB, Felde VA, Bjune AE, Grytnes J-A, Seppä H and Giesecke T (2016) Does pollen-assemblage richness reflect floristic richness? A review of recent developments and future challenges. *Review of Palaeobotany and Palynology*, **228**, 1–25. doi:10.1016/j.revpalbo.2015.12.011.

Blank TO (2005) Gay Men and Prostate Cancer: Invisible Diversity. *Journal of Clinical Oncology*, **23**(12), 2593–2596. doi:10.1200/JCO.2005.00.968.

Boom PPG van den (2021) New records of Bacidina (Ramalinaceae) and Xenonectriella (Nectriaceae) from the Netherlands with special notes on some smaller Bacidina species. *Herzogia*, **34**(2), 519–523. doi:10.13158/heia.34.2.2021.519.

Boussarie G, Bakker J, Wangensteen OS, Mariani S, Bonnin L, Juhel J-B, Kiszka JJ, Kulbicki M, Manel S, Robbins WD, Vigliola L and Mouillot, D (2018) Environmental DNA illuminates the dark diversity of sharks. *Science Advances*, **4**(5), eaap9661. doi:10.1126/sciadv.aap9661.

Bråte J, Klaveness D, Rygh T, Jakobsen KS and Shalchian-Tabrizi K (2010) Telonemia-specific environmental 18S rDNA PCR reveals unknown diversity and multiple marine-freshwater colonizations. *BMC Microbiology*, **10**(1), 168. doi:10.1186/1471-2180-10-168.

Bronne L, Valdueza M and Margry CJPJ (2022) Westward expansion of the known range of the semi-slug Vitrinobrachium breve (A. Férussac, 1821) (Gastropoda, Pulmonata: Vitrinidae) with comments on the use of citizen science in snails. *North-Western Journal of Zoology*, **18**.

Brownstein CD, Kim D, Orr OD, Hogue GM, Tracy BH, Pugh MW, Singer R, Myles-McBurney C, Mollish JM, Simmons JW, David SR, Watkins-Colwell G, Hoffman EA and Near TJ (2022) Hidden species diversity in an iconic living fossil vertebrate. *Biology Letters*, **18**(11), 20220395. doi:10.1098/rsbl.2022.0395.

Bruno A, Sandionigi A, Rizzi E, Bernasconi M, Vicario S, Galimberti A, Cocuzza C, Labra M and Casiraghi M (2017) Exploring the under-investigated “microbial dark matter” of drinking water treatment plants. *Scientific Reports*, **7**, 44350. doi:10.1038/srep44350.

Caković D, Stešević D, Schönswetter P and Frajman B (2018) Long neglected diversity in the Accursed Mountains of northern Albania: Cerastium hekuravense is genetically and morphologically divergent from C. dinaricum. *Plant Systematics and Evolution*, **304**(1), 57–69. doi:10.1007/s00606-017-1448-1.

Caldwell MW and Chatterton BDE (1995) Phylogenetic analysis of some Silurian rostroconchs (Mollusca) from northwestern Canada. *Canadian Journal of Earth Sciences*, **32**(6), 806–827. doi:10.1139/e95-068.

Catania M del V and Romero AI (2005) Two new species of Camarops (Boliniaceae, Ascomycotina) and a key to Argentinean species. Retrieved from https://ri.conicet.gov.ar/handle/11336/29966

Cavalcanti DR and Albuquerque UP (2013) The “Hidden Diversity” of Medicinal Plants in Northeastern Brazil: Diagnosis and Prospects for Conservation and Biological Prospecting. *Evidence-Based Complementary and Alternative Medicine*, **2013**, e102714. doi:10.1155/2013/102714.

Chao A, Chiu C-H, Colwell RK, Magnago LFS, Chazdon RL and Gotelli NJ (2017a) Deciphering the enigma of undetected species, phylogenetic, and functional diversity based on Good-Turing theory. *Ecology*, **98**(11), 2914–2929. doi:10.1002/ecy.2000.

Chao A, Colwell RK, Chiu C-H and Townsend D (2017b) Seen once or more than once: applying Good-Turing theory to estimate species richness using only unique observations and a species list. *METHODS IN ECOLOGY AND EVOLUTION*, **8**(10), 1221–1232. doi:10.1111/2041-210X.12768.

Chao A, Hsieh TC, Chazdon RL, Colwell RK and Gotelli NJ (2015) Unveiling the species-rank abundance distribution by generalizing the Good-Turing sample coverage theory. *Ecology*, **96**(5), 1189–1201. doi:10.1890/14-0550.1.

Chao A and Shen T-J (2004) Nonparametric prediction in species sampling. *Journal of Agricultural, Biological, and Environmental Statistics*, **9**(3), 253–269. doi:10.1198/108571104X3262.

Choi SS, Park SJ, Bum HM, Moon M-O, Kim D-S, Ahn U-S, Lee SH and Kim CH (2020) Unrecorded liverwort species from Korean flora IV: new data on Riccia L. (Marchantiophyta) from Jeju UNESCO World Natural Heritage. *Journal of Asia-Pacific Biodiversity*, **13**(4), 745–748. doi:10.1016/j.japb.2020.06.012.

Claridge AW, Mifsud G, Dawson J, Saxon MJ, Claridge AW, Mifsud G, Dawson J and Saxon MJ (2004) Use of infrared digital cameras to investigate the behaviour of cryptic species. *Wildlife Research*, **31**(6), 645–650. doi:10.1071/WR03072.

Costello MJ, Lane M, Wilson S and Houlding B (2015) Factors influencing when species are first named and estimating global species richness. *Global Ecology and Conservation*, **4**, 243–254. doi:10.1016/j.gecco.2015.07.001.

Craig CW and Felder DL (2022) Two new marine hermit crabs allied with the Paguristes tortugae complex (Crustacea: Decapoda: Anomura) from the western Atlantic. *Zootaxa*, **5178**(1), 1–25. doi:10.11646/zootaxa.5178.1.1.

Damm S, Dijkstra K-DB and Hadrys H (2010) Red drifters and dark residents: The phylogeny and ecology of a Plio-Pleistocene dragonfly radiation reflects Africa’s changing environment (Odonata, Libellulidae, Trithemis). *Molecular Phylogenetics and Evolution*, **54**(3), 870–882. doi:10.1016/j.ympev.2009.12.006.

de Bello F, Fibich P, Zelený D, Kopecký M, Mudrák O, Chytrý M, Pyšek P, Wild J, Michalcová D, Sádlo J, Šmilauer P, Lepš J and Pärtel M (2016) Measuring size and composition of species pools: a comparison of dark diversity estimates. *Ecology and Evolution*, **6**(12), 4088–4101. doi:10.1002/ece3.2169.

De Clerck O, Guiry MD, Leliaert F, Samyn Y and Verbruggen H (2013) Algal taxonomy: a road to nowhere? *Journal of Phycology*, **49**(2), 215–225. doi:10.1111/jpy.12020.

De Laubenfels DJ and Adema F (1998) A taxonomic revision of the genera Cycas and Epicycas gen. nov. (Cycadaceae). *Blumea: Biodiversity, Evolution and Biogeography of Plants*, **43**, 351–400.

Decker P (2016) Phylogenetic analysis of the Australian trans-Bass Strait millipede genus Pogonosternum (Carl, 1912) (Diplopoda, Polydesmida, Paradoxosomatidae) indicates multiple glacial refugia in southeastern Australia. *ZooKeys*, **578**, 15–31. doi:10.3897/zookeys.578.8052.

del Carmen H. Rodríguez M, Evans HC, de Abreu LM, de Macedo DM, Ndacnou MK, Bekele KB and Barreto RW (2021) New species and records of Trichoderma isolated as mycoparasites and endophytes from cultivated and wild coffee in Africa. *Scientific Reports*, **11**(1), 5671. doi:10.1038/s41598-021-84111-1.

Delić T, Trontelj P, Rendoš M and Fišer C (2017) The importance of naming cryptic species and the conservation of endemic subterranean amphipods. *Scientific Reports*, **7**(1), 3391. doi:10.1038/s41598-017-02938-z.

DeSalle R and Goldstein P (2019) Review and Interpretation of Trends in DNA Barcoding. *Frontiers in Ecology and Evolution*, **7**. Retrieved from https://www.frontiersin.org/article/10.3389/fevo.2019.00302

Doucette A (2012) Dracula agnosia (Orchidaceae: Pleurothallidinae), a long confused undescribed species. *Phytotaxa*, **56**, 23–27. doi:10.11646/phytotaxa.56.1.6.

Dumont KP and Batra LR (1978) Sclerotiniaceae XIII. An Undescribed Species on Berberis. *Mycologia*, **70**(1), 61–67. doi:10.1080/00275514.1978.12020202.

Durigon J, Sperotto P, Ferreira PPA, Dettke GA, Záchia RA, Farinaccio MA, Seger GD dos S and Miotto STS (2019) Updates on extratropical region climbing plant flora: news regarding a still-neglected diversity. *Acta Botanica Brasilica*, **33**, 644–653. doi:10.1590/0102-33062018abb0333.

Ebejer MJ and Barták M (2019) An annotated list of the Chamaemyiidae (Diptera, Acalyptrata) of Turkey with new records and additional data. *ZooKeys*, **838**, 35–48. doi:10.3897/zookeys.838.33027.

Eitner BJ (1995) Systematics of the Genus Alopias (Lamniformes: Alopiidae) with Evidence for the Existence of an Unrecognized Species. *Copeia*, **1995**(3), 562–571. doi:10.2307/1446753.

Feng KH, Brown JD, Turner GG, Holmes EC and Allison AB (2022) Unrecognized diversity of mammalian orthoreoviruses in North American bats. *Virology*, **571**, 1–11. doi:10.1016/j.virol.2022.03.012.

Fernandes CS, Batalha MA and Bichuette ME (2019) Dark diversity in the dark: a new approach to subterranean conservation. *Subterranean Biology*, **32**, 69–80. doi:10.3897/subtbiol.32.38121.

Fernandes IO, Souza JLP, C FF, Delabie JHC and Schultz TR (2015) A new species of Simopelta (Hymenoptera: Formicidae: Ponerinae) from Brazil and Costa Rica. *Zootaxa*, **3956**(2), 295–300. doi:10.11646/zootaxa.3956.2.10.

Fišer C, Robinson CT and Malard F (2018) Cryptic species as a window into the paradigm shift of the species concept. *Molecular Ecology*, **27**(3), 613–635. doi:10.1111/mec.14486.

Fisher MA, Vinson JE, Gittleman JL and Drake JM (2018) The description and number of undiscovered mammal species. *Ecology and Evolution*, **8**(7), 3628–3635. doi:10.1002/ece3.3724.

Fløjgaard C, Valdez JW, Dalby L, Moeslund JE, Clausen KK, Ejrnæs R, Pärtel M and Brunbjerg AK (2020) Dark diversity reveals importance of biotic resources and competition for plant diversity across habitats. *Ecology and Evolution*, **10**(12), 6078–6088. doi:10.1002/ece3.6351.

Francis CM, Borisenko AV, Ivanova NV, Eger JL, Lim BK, Guillén-Servent A, Kruskop SV, Mackie I and Hebert PDN (2010) The Role of DNA Barcodes in Understanding and Conservation of Mammal Diversity in Southeast Asia. *PLoS ONE*, **5**(9), e12575. doi:10.1371/journal.pone.0012575.

Fujikura, K, Lindsay, D, Kitazato, H, Nishida, S, and Shirayama, Y (2010) Marine Biodiversity in Japanese Waters. *PLoS ONE*, **5**(8), e11836. doi:10.1371/journal.pone.0011836.

Fusco-Costa R, Ingberman B, Magezi GS and Monteiro-Filho EL de A (2023) Present but not detected: new records increase the jaguar's area of occupancy in the coastal Atlantic Forest. *Oryx*, **57**(1), 72–75. doi:10.1017/S0030605322000151.

García-Avendaño EI, and Guerrero RJ (2018) Taxonomy and distribution of Tapinoma ants (Formicidae: Dolichoderinae) in Colombia. *Revista Colombiana de Entomología*, **44**(2), 223–237.

Garrard GE, Bekessy SA, McCarthy MA and Wintle BA (2015) Incorporating detectability of threatened species into environmental impact assessment. *Conservation Biology*, **29**(1), 216–225. doi:10.1111/cobi.12351.

Giam X, Ng TH, Yap VB and Tan HTW (2010) The extent of undiscovered species in Southeast Asia. *Biodiversity and Conservation*, **19**(4), 943–954. doi:10.1007/s10531-010-9792-2.

Giam X, Scheffers BR, Sodhi NS, Wilcove DS, Ceballos G and Ehrlich PR (2011) Reservoirs of richness: least disturbed tropical forests are centres of undescribed species diversity. *Proceedings of the Royal Society B: Biological Sciences*, **279**(1726), 67–76. doi:10.1098/rspb.2011.0433.

Godeas AM and Arambarri AM (1996) Helicoon septatissimum sp. nov., a new species from Tierra del Fuego (Argentina). *Mycotaxon*, **60**, 481–484.

Gorbalenya AE and Siddell SG (2021) Recognizing species as a new focus of virus research. *PLoS Pathogens*, **17**(3), e1009318. doi:10.1371/journal.ppat.1009318.

Gotelli NJ, Dorazio RM, Ellison AM and Grossman GD (2010) Detecting temporal trends in species assemblages with bootstrapping procedures and hierarchical models. *Philosophical Transactions of the Royal Society B: Biological Sciences*, **365**(1558), 3621–3631. doi:10.1098/rstb.2010.0262.

Grabowski M, Wysocka A and Mamos T (2017) Molecular species delimitation methods provide new insight into taxonomy of the endemic gammarid species flock from the ancient Lake Ohrid. *Zoological Journal of the Linnean Society*, **181**(2), 272–285. doi:10.1093/zoolinnean/zlw025.

Gustafsson DR, Zou F, Oslejskova L, Najer T and Sychra O (2019) Four new species of Brueelia Kéler, 1936 (Phthiraptera: Ischnocera) from African hosts, with a redescription of Nirmus bicurvatus Piaget, 1880. *European Journal of Taxonomy*, (507). doi:10.5852/ejt.2019.507.

Hansel CM, Ferdelman TG and Tebo BM (2015) Cryptic Cross-Linkages Among Biogeochemical Cycles: Novel Insights from Reactive Intermediates. *Elements*, **11**(6), 409–414. doi:10.2113/gselements.11.6.409.

Hanspach J, Kühn I, Schweiger O, Pompe S and Klotz S (2011) Geographical patterns in prediction errors of species distribution models. *Global Ecology and Biogeography*, **20**(5), 779–788. doi:10.1111/j.1466-8238.2011.00649.x.

Hawksworth D (2004) Fungal diversity and its implications for genetic resource collections. *Stud. Mycol.*, **50**.

Hawksworth DL and Rossman AY (1997) Where Are All the Undescribed Fungi? *Phytopathology®*, **87**(9), 888–891. doi:10.1094/PHYTO.1997.87.9.888.

Hebert PDN, Penton EH, Burns JM, Janzen DH and Hallwachs W (2004) Ten species in one: DNA barcoding reveals cryptic species in the neotropical skipper butterfly Astraptes fulgerator. *Proceedings of the National Academy of Sciences*, **101**(41), 14812–14817. doi:10.1073/pnas.0406166101.

Hedges SB and Garrido OH (1992) Cuban Snakes of the Genus arrhyton: Two New Species and a Reconsideration of A. Redimitum Cope. *Herpetologica*, **48**(2), 168–177.

Hohlfeld M and Arndt H (2022) Changes in the Abundance and Taxonomic Composition of Benthic Heterotrophic Protists from Atlantic Sublittoral to Deep-Sea Sediments. *Diversity*, **14**(3), 164. doi:10.3390/d14030164.

Horton T, Cooper H, Vlierboom R, Thurston M, Hauton C and Young CR (2020) Molecular phylogenetics of deep-sea amphipods (Eurythenes) reveal a new undescribed species at the Porcupine Abyssal Plain, North East Atlantic Ocean. *Progress in Oceanography*, **183**, 102292. doi:10.1016/j.pocean.2020.102292.

Hsieh Y-L and Linsenmair KE (2011) Underestimated spider diversity in a temperate beech forest. *Biodiversity and Conservation*, **20**(13), 2953–2965. doi:10.1007/s10531-011-0158-1.

Huang Y-S, and Shen F-T (2016) Bioprospecting of facultatively oligotrophic bacteria from non-rhizospheric soils. *Applied Soil Ecology*, **108**, 315–324. doi:10.1016/j.apsoil.2016.09.004.

Huber BA, Caspar KR, Eberle J, Huber BA, Caspar KR and Eberle J (2019) New species reveal unexpected interspecific microhabitat diversity in the genus Uthina Simon, 1893 (Araneae : Pholcidae). *Invertebrate Systematics*, **33**(1), 181–207. doi:10.1071/IS18002.

Huber BA and Chao A (2019) Inferring global species richness from megatransect data and undetected species estimates. *Contributions to Zoology*, **88**(1), 42–53. doi:10.1163/18759866-20191347.

Humala AE, Lee J-W and Choi J-K (2022) Checklist of the subfamily Orthocentrinae (Hymenoptera: Ichneumonidae) with seven unrecorded species from Korea. *Journal of Asia-Pacific Biodiversity*, **15**(4), 557–567. doi:10.1016/j.japb.2022.05.008.

Hurrell JA and Puentes JP (2017) Plant species and products of the Traditional Chinese Phytotherapy in the Ciudade Autónoma de Buenos Aires, Argentina. *Ethnobiology and Conservation*, **6**. doi:10.15451/ec2017-02-6.1-1-43.

Iknayan KJ, Tingley MW, Furnas BJ and Beissinger SR (2014) Detecting diversity: emerging methods to estimate species diversity. *Trends in Ecology & Evolution*, **29**(2), 97–106. doi:10.1016/j.tree.2013.10.012.

Issaris Y, Katsanevakis S, Salomidi M, Tsiamis K, Katsiaras N and Verriopoulos G (2012) Occupancy estimation of marine species: dealing with imperfect detectability. *Marine Ecology Progress Series*, **453**, 95–106. doi:10.3354/meps09668.

Ito M, Watanabe K and Maeto K (2015) Molecular evidence resolving the confusion of two species of Spilopteron (Hymenoptera: Ichneumonidae) caused by marked geographical colour variation. *EJE*, **112**(3), 543–556. doi:10.14411/eje.2015.068.

Janosik AM and Halanych KM (2010) Unrecognized Antarctic Biodiversity: A Case Study of the Genus Odontaster (Odontasteridae; Asteroidea). *Integrative and Comparative Biology*, **50**(6), 981–992. doi:10.1093/icb/icq119.

Jofre, GI, Singh, A, Mavengere, H, Sundar G, D'Agostino E, Chowdhary A and Matute, DR (2022) An Indian lineage of Histoplasma with strong signatures of differentiation and selection. *Fungal Genetics and Biology*, **158**, 103654. doi:10.1016/j.fgb.2021.103654.

Johnson RA and Rutowski RL (2022) Color, activity period, and eye structure in four lineages of ants: Pale, nocturnal species have evolved larger eyes and larger facets than their dark, diurnal congeners. *PLoS ONE*, **17**(9), e0257779. doi:10.1371/journal.pone.0257779.

Joppa LN, Roberts DL, Myers N and Pimm SL (2011) Biodiversity hotspots house most undiscovered plant species. *Proceedings of the National Academy of Sciences*, **108**(32), 13171–13176. doi:10.1073/pnas.1109389108.

Jorna J, Linde JB, Searle PC, Jackson AC, Nielsen M-E, Nate MS, Saxton NA, Grewe F, Herrera-Campos M de los A, Spjut RW, Wu H, Ho B, Lumbsch HT and Leavitt SD (2021) Species boundaries in the messy middle—A genome-scale validation of species delimitation in a recently diverged lineage of coastal fog desert lichen fungi. *Ecology and Evolution*, **11**(24), 18615–18632. doi:10.1002/ece3.8467.

Kasparek M (2019) A new species in the Trachusa ovata species group (Hymenoptera: Apoidea) from Peninsular Malaysia with an overview of the old-world species within the genus Trachusa. *Journal of Natural History*, **53**(17–18), 1079–1094. doi:10.1080/00222933.2019.1632953.

Kim C-J, Mita T and Lee J-W (2012) One new species and three unrecorded species of Anteoninae (Hymenoptera: Dryinidae) from South Korea. *Entomological Research*, **42**(2), 99–103. doi:10.1111/j.1748-5967.2011.00364.x.

Kim S, Heo U-H and Lee S (2020) New record of genus Depressaria Haworth (Lepidoptera: Depressariidae) from Korea: A checklist of the genus in Korea with new biological data. *Journal of Asia-Pacific Biodiversity*, **13**(4), 594–598. doi:10.1016/j.japb.2020.06.015.

Klarica J, Bittner L, Pallua J, Pezzei C, Huck-Pezzei V, Dowell F, Schied J, Bonn GK, Huck C, Schlick-Steiner BC and Steiner, FM (2011) Near-Infrared Imaging Spectroscopy as a Tool to Discriminate Two Cryptic Tetramorium Ant Species. *Journal of Chemical Ecology*, **37**(6), 549–552. doi:10.1007/s10886-011-9956-x.

Kortmann M, Roth N, Buse J, Hilszczański J, Jaworski T, Morinière J, Seidl R, Thorn S and Müller JC (2022) Arthropod dark taxa provide new insights into diversity responses to bark beetle infestations. *Ecological Applications*, **32**(2), e2516. doi:10.1002/eap.2516.

Kumar V, Griswold T and Belavadi VV (2017) The Resin and Carder bees of south India (Hymenoptera: Megachilidae: Anthidiini). *Zootaxa*, **4317**(3), 436–468. doi:10.11646/zootaxa.4317.3.2.

Kynčlová A, Škaloud P and Škaloudová M (2010) Unveiling hidden diversity in the Synura petersenii species complex (Synurophyceae, Heterokontophyta). *Nova Hedwigia, Beihefte*, 283–298. doi:10.1127/1438-9134/2010/0136-0283.

Lauriault P and Wiersma YF (2019) Reducing the rate of false absences of cryptic species in inventory and sampling work. *The Bryologist*, **122**(4), 578–585. doi:10.1639/0007-2745-122.4.578.

Lavin M, Pennington RT, Hughes CE, Lewis GP, Delgado-Salinas A, Duno de Stefano R, de Queiroz LP, Cardoso D and Wojciechowski MF (2018) DNA Sequence Variation among Conspecific Accessions of the Legume Coursetia caribaea Reveals Geographically Localized Clades Here Ranked as Species. *Systematic Botany*, **43**(3), 664–675. doi:10.1600/036364418X697382.

Lehnert M, Kottke I, Setaro S, Pazmiño LF, Suárez JP and Kessler M (2009) Mycorrhizal Associations in Ferns from Southern Ecuador. *American Fern Journal*, **99**(4), 292–306. doi:10.1640/0002-8444-99.4.292.

Liu M, Liu Y, Zhang T, Lu B, Gao F, Gu J, Al-Farraj SA, Hu X and Song W (2022) Integrative studies on the taxonomy and molecular phylogeny of four new Pleuronema species (Protozoa, Ciliophora, Scuticociliatia). *Marine Life Science & Technology*, **4**(2), 179–200. doi:10.1007/s42995-022-00130-5.

Long WH (1916) Five Undescribed Species of Ravenelia. *Botanical Gazette*, **61**(5), 417–424. doi:10.1086/331797.

Lopes, AV, Porto, RG, Cruz-Neto, O, … Tabarelli, M (2021) Neglected diversity of crop pollinators: Lessons from the world's largest tropical country. *Perspectives in Ecology and Conservation*, **19**(4), 500–504. doi:10.1016/j.pecon.2021.06.004.

López, H, Contreras-Díaz, HG, Oromí, P, and Juan, C (2007) Delimiting species boundaries for endangered Canary Island grasshoppers based on DNA sequence data. *Conservation Genetics*, **8**(3), 587–598. doi:10.1007/s10592-006-9199-5.

Lopez-Vaamonde, C, Sire, L, Rasmussen, B, … Lees, DC (2019) DNA barcodes reveal deeply neglected diversity and numerous invasions of micromoths in Madagascar. *Genome*, **62**(3), 108–121. doi:10.1139/gen-2018-0065.

Lu M, Gao L, Li H and He F (2021) The patterns of vascular plant discoveries in China. *Ecology and Evolution*, **11**(18), 12378–12388. doi:10.1002/ece3.7971.

Lücking R (2012) Predicting species richness in tropical lichenized fungi with 'modular' combinations of character states. *Biodiversity and Conservation*, **21**(9), 2341–2360. doi:10.1007/s10531-011-0217-7.

Lumbsch HT, Messuti MI and Iii THN (2003) New or Overlooked Species in the Lecanora subfusca Group from Southwestern North America (Lecanorales, Ascomycotina). *The Bryologist*, **106**(4), 552–559. doi:10.1639/0007-2745(2003)106[552:NOOSIT]2.0.CO;2.

Luna N, Pessoa EM and Alves M (2021) Taxonomic Study of the Species of Maranta Plum ex. L. (Marantaceae) from Northeastern Brazil: A Neglected Diversity Center for the Genus with Five New Species. *Systematic Botany*, **46**(3), 582–610. doi:10.1600/036364421X16312067913345.

Madani GF (2021) Range extensions and habitat use of Forrest's mouse (Leggadina forresti) in New South Wales. *Australian Mammalogy*, **44**(1), 143–148. doi:10.1071/AM20036.

Maldonado C, Persson C, Alban J, Antonelli A and Rønsted N (2017) Cinchona anderssonii (Rubiaceae), a new overlooked species from Bolivia. *Phytotaxa*, **297**(2), 203–208. doi:10.11646/phytotaxa.297.2.8.

Mannerkoski I (2000) *Clambus nigrellus* Reitter (Coleoptera: Clambidae), an overlooked beetle species in Finland. *Entomologica Fennica*, **11**(4), 229–230. doi:10.33338/ef.84071.

Mao CX and Colwell RK (2005) Estimation of Species Richness: Mixture Models, the Role of Rare Species, and Inferential Challenges. *Ecology*, **86**(5), 1143–1153. doi:10.1890/04-1078.

Marko PB and Moran AL (2009) Out of sight, out of mind: high cryptic diversity obscures the identities and histories of geminate species in the marine bivalve subgenus Acar. *Journal of Biogeography*, **36**(10), 1861–1880. doi:10.1111/j.1365-2699.2009.02114.x.

Martínez-Azorín M, Pinter M, Crespo MB, Alonso-Vargas MÁ and Wetschnig W (2018) Massonia visseriae (Asparagaceae, Scilloideae): rediscovery of a neglected species of Neobakeria and its transfer to Massonia. *Phytotaxa*, **334**(1), 70–74. doi:10.11646/phytotaxa.334.1.11.

Mátis A, Malkócs T, Kuhn T, Laczkó L, Moysiyenko I, Szabó A, Bădărău AS and Sramkó G (2022) Hiding in plain sight: Integrative analyses uncover a cryptic Salvia species in Europe. *TAXON*, **72**(1), 78–97. doi:10.1002/tax.12818.

McDonald PJ, Brown RM, Kraus F, Bowles P, Arifin U, Eliades SJ, Fisher RN, Gaulke M, Grismer LL, Ineich I, Karin BR, Meneses CG, Richards SJ, Sanguila MB, Siler CD and Oliver PM (2022) Cryptic extinction risk in a western Pacific lizard radiation. *Biodiversity and Conservation*, **31**(8), 2045–2062. doi:10.1007/s10531-022-02412-x.

Mejdalani G and Cavichioli RR (2014) The Sharpshooter Genus Geitogonalia Young: Descriptions of a New Brazilian Species and of the Female of Geitogonalia quatuordecimmaculata (Taschenberg) (Hemiptera: Cicadellidae). *Neotropical Entomology*, **43**(4), 373–379. doi:10.1007/s13744-014-0220-2.

Metusala D (2019) A New Name for an Overlooked Species of Eulophia (Orchidaceae) from Wallacea. *Phytotaxa*, **415**(4), 217–224. doi:10.11646/phytotaxa.415.4.6.

Milić D, Radenković S, Ačanski J and Vujić A (2019) The importance of hidden diversity for insect conservation: a case study in hoverflies (the Merodon atratus complex, Syrphidae, Diptera). *Journal of Insect Conservation*, **23**(1), 29–44. doi:10.1007/s10841-018-0111-7.

Minton RL and Lydeard C (2003) Phylogeny, taxonomy, genetics and global heritage ranks of an imperilled, freshwater snail genus Lithasia (Pleuroceridae). *Molecular Ecology*, **12**(1), 75–87. doi:10.1046/j.1365-294X.2003.01719.x.

Miranda GFG (2017) Identification key for the genera of Syrphidae (Diptera) from the Brazilian Amazon and new taxon records. *Acta Amazonica*, **47**, 53–62. doi:10.1590/1809-4392201601022.

Mohankumar NM and Hefley TJ (2022) Using machine learning to model nontraditional spatial dependence in occupancy data. *Ecology*, **103**(2), e03563. doi:10.1002/ecy.3563.

Montgomery FA, Reid SM and Mandrak NE (2021) Imperfect detection biases extinction-debt assessments. *Conservation Science and Practice*, **3**(7), e427. doi:10.1111/csp2.427.

Moraes S de S, Murillo-Ramos L, Machado PA, Ghanavi HR, Magaldi LM, Silva-Brandão KL, Kato MJ, Freitas AVL and Wahlberg N (2021) A double-edged sword: Unrecognized cryptic diversity and taxonomic impediment in Eois (Lepidoptera, Geometridae). *Zoologica Scripta*, **50**(5), 633–646. doi:10.1111/zsc.12488.

Morinière J, Balke M, Doczkal D, Geiger MF, Hardulak LA, Haszprunar G, Hausmann A, Hendrich L, Regalado L, Rulik B, Schmidt S, Wägele J-W and Hebert PDN (2019) A DNA barcode library for 5,200 German flies and midges (Insecta: Diptera) and its implications for metabarcoding-based biomonitoring. *Molecular Ecology Resources*, **19**(4), 900–928. doi:10.1111/1755-0998.13022.

Moura MR and Jetz W (2021) Shortfalls and opportunities in terrestrial vertebrate species discovery. *Nature Ecology & Evolution*, **5**(5), 631–639. doi:10.1038/s41559-021-01411-5.

Muis A, Ryley MJ, Tan YP, Suharjo R, Nonci N, Danaatmadja Y, Hidayat I, Widiastuti A, Widinugraheni S, Shivas RG and Thines M (2023) Peronosclerospora neglecta sp. nov.—a widespread and overlooked threat to corn (maize) production in the tropics. *Mycological Progress*, **22**(2), 12. doi:10.1007/s11557-022-01862-5.

Nilsson RH, Larsson K-H, Taylor AFS, Bengtsson-Palme J, Jeppesen TS, Schigel D, Kennedy P, Picard K, Glöckner FO, Tedersoo L, Saar I, Kõljalg U and Abarenkov K (2019) The UNITE database for molecular identification of fungi: handling dark taxa and parallel taxonomic classifications. *Nucleic Acids Research*, **47**(D1), D259–D264. doi:10.1093/nar/gky1022.

Nupponen K and Junnilainen J (1998) *Ephestia animella* sp. n. (Lepidoptera: Pyralidae, Phycitinae) from the Ural Mountains and southern Siberia. *Entomologica Fennica*, **9**(3), 143–146. doi:10.33338/ef.83977.

Nuryadi H, Sumimoto S, Teruya T, Suenaga K and Suda S (2020) Characterization of Macroscopic Colony-Forming Filamentous Cyanobacteria from Okinawan Coasts as Potential Sources of Bioactive Compounds. *Marine Biotechnology*, **22**(6), 824–835. doi:10.1007/s10126-020-10010-7.

Oberprieler SK, Andersen AN and Moritz CC (2018) Ants in Australia's Monsoonal Tropics: CO1 Barcoding Reveals Extensive Unrecognised Diversity. *Diversity*, **10**(2), 36. doi:10.3390/d10020036.

Orange A (2004) The Verrucaria fuscella group in Great Britain and Ireland. *The Lichenologist*, **36**(3–4), 173–182. doi:10.1017/S0024282904014252.

Orihara T and Smith ME (2017) Unique phylogenetic position of the African truffle-like fungus, Octaviania ivoryana (Boletaceae, Boletales), and the proposal of a new genus, Afrocastellanoa. *Mycologia*, **109**(2), 323–332. doi:10.1080/00275514.2017.1301750.

Ota RR, Message HJ, Graça WJ da and Pavanelli CS (2015) Neotropical Siluriformes as a Model for Insights on Determining Biodiversity of Animal Groups. *PLoS ONE*, **10**(7), e0132913. doi:10.1371/journal.pone.0132913.

PAcheco C, Carballo JL, Cruz-Barraza JA, Schönberg CHL and Calcinai B (2020) Delimiting boundaries between species: excavating sponges close to Cliona mucronata (Demospongiae). *Systematics and Biodiversity*, **18**(6), 573–591. doi:10.1080/14772000.2020.1776785.

Packer L and Taylor JS (1997) Forum: How many species are there? An appliation of the phylogenetic species concept to genetic data for some comparatively well known bee "species." *The Canadian Entomologist*, **129**(4), 587–594. doi:10.4039/Ent129587-4.

Page R (2011, April 12) iPhylo: Dark taxa: GenBank in a post-taxonomic world. Retrieved from https://iphylo.blogspot.com/2011/04/dark-taxa-genbank-in-post-taxonomic.html

Page RDM (2016) DNA barcoding and taxonomy: dark taxa and dark texts. *Philosophical Transactions of the Royal Society B: Biological Sciences*, **371**(1702), 20150334. doi:10.1098/rstb.2015.0334.

Pärtel M (2014) Community ecology of absent species: hidden and dark diversity. *Journal of Vegetation Science*, **25**(5), 1154–1159. doi:10.1111/jvs.12169.

Pärtel M, Szava-Kovats R and Zobel M (2011) Dark diversity: shedding light on absent species. *Trends in Ecology & Evolution*, **26**(3), 124–128. doi:10.1016/j.tree.2010.12.004.

Payo-Payo A and Lobo JM (2016) The unpredictable characteristics of the localities where new Iberian species will be discovered. *Diversity and Distributions*, **22**(1), 73–82. doi:10.1111/ddi.12386.

Pereira JBS and Prado J (2022) Taxonomy of Cryptic Isoëtes Species from the Brazilian Semi-Arid Region, with a Note about the Putative Maternal Origin of Polyploids. *American Fern Journal*, **112**(3), 190–206. doi:10.1640/0002-8444-112.3.190.

Peters U (2021) Hidden figures: epistemic costs and benefits of detecting (invisible) diversity in science. *European Journal for Philosophy of Science*, **11**(1), 33. doi:10.1007/s13194-021-00349-6.

Phinney NH, Gauslaa Y, Palmqvist K and Esseen P-A (2021) Macroclimate drives growth of hair lichens in boreal forest canopies. *Journal of Ecology*, **109**(1), 478–490. doi:10.1111/1365-2745.13522.

Popova M (2018) Inactionable/Unspeakable: Bisexuality in the Workplace. *Journal of Bisexuality*, **18**(1), 54–66. doi:10.1080/15299716.2017.1383334.

Prihatini I, Glen M, Wardlaw TJ and Mohammed CL (2016) Diversity and identification of fungi associated with needles of Pinus radiata in Tasmania. *Southern Forests: A Journal of Forest Science*, **78**(1), 19–34. doi:10.2989/20702620.2015.1092345.

Puentes JP, Arenas PM and Hurrell JA (2019) Chinese functional foods and nutraceuticals: plants and products commercialized in the Ciudad Autónoma de Buenos Aires, Argentina. *Ethnobiology and Conservation*, **8**. Retrieved from https://ethnobioconservation.com/index.php/ebc/article/view/293

Reumont V and Marcus B (2018) Studying Smaller and Neglected Organisms in Modern Evolutionary Venomics Implementing RNASeq (Transcriptomics)—A Critical Guide. *Toxins*, **10**(7), 292. doi:10.3390/toxins10070292.

Rhinehart TA, Turek D and Kitzes J (2022) A continuous-score occupancy model that incorporates uncertain machine learning output from autonomous biodiversity surveys. *Methods in Ecology and Evolution*, **13**(8), 1778–1789. doi:10.1111/2041-210X.13905.

Říčan O, Říčanová Š, Rodriguez Haro LR and Rodriguez Haro CE (2022) Unrecognized species diversity and endemism in the cichlid genus Bujurquina (Teleostei: Cichlidae) together with a molecular phylogeny document large-scale transformation of the western Amazonian river network and reveal complex paleogeography of the Ecuadorian Amazon. *Hydrobiologia*, **850**(10), 2199–2229. doi:10.1007/s10750-022-05019-z.

Riva F, Gentile G, Bonelli S, Acorn JH, Denes FV, Crosby AD and Nielsen SE (2020) Of detectability and camouflage: evaluating Pollard Walk rules using a common, cryptic butterfly. *Ecosphere*, **11**(4), e03101. doi:10.1002/ecs2.3101.

Roh SJ, Banasiak G and Byun B-K (2016) A new and an unrecorded species of the family Psychidae (Lepidoptera) from Korea, with an annotated catalogue. *Journal of Natural History*, **50**(11–12), 669–680. doi:10.1080/00222933.2015.1082654.

Ronk A, Szava-Kovats R and Pärtel M (2015) Applying the dark diversity concept to plants at the European scale. *Ecography*, **38**(10), 1015–1025. doi:10.1111/ecog.01236.

Ross JL (2016) The Dark Matter of Biology. *Biophysical Journal*, **111**(5), 909–916. doi:10.1016/j.bpj.2016.07.037.

Roth N, Hacker HH, Heidrich L, Friess N, García-Barros E, Habel JC, Thorn S and Müller J (2021) Host specificity and species colouration mediate the regional decline of nocturnal moths in central European forests. *Ecography*, **44**(6), 941–952. doi:10.1111/ecog.05522.

Rowley JJL, Tran DTA, Frankham GJ, Dekker AH, Le DTT, Nguyen TQ, Dau VQ and Hoang HD (2015) Undiagnosed Cryptic Diversity in Small, Microendemic Frogs (Leptolalax) from the Central Highlands of Vietnam. *PLoS ONE*, **10**(5), e0128382. doi:10.1371/journal.pone.0128382.

Ryberg M and Nilsson RH (2018) New light on names and naming of dark taxa. *MycoKeys*, (30), 31–39. doi:10.3897/mycokeys.30.24376.

Saha S, Song N, Yu Z, Baki MA, McKay RJ, Qin J and Gao T (2022) Descriptions of Two New Species, Sillago muktijoddhai sp. nov. and Sillago mengjialensis sp. nov. (Perciformes: Sillaginidae) from the Bay of Bengal, Bangladesh. *Fishes*, **7**(3), 93. doi:10.3390/fishes7030093.

Sampaio R, Munari DP, Röhe F, Ravetta AL, Rubim P, Farias IP, Silva MNF da and Cohn-Haft M (2010) New distribution limits of Bassaricyon alleni Thomas 1880 and insights on an overlooked species in the Western Brazilian Amazon., **74**(3), 323–327. doi:10.1515/mamm.2010.008.

Schilthuizen M, Thompson C, Vries R de, Peursen A van, Paterno M, Maestri S, Marcolongo L, Esposti C, Delledonne M and Njunjić I (2022) A new giant keelback slug of the genus Limax from the Balkans, described by citizen scientists. *Biodiversity Data Journal*, **10**, e69685. doi:10.3897/BDJ.10.e69685.

Schmidt RC, Bart HL and Nyingi WD (2015) Two new species of African suckermouth catfishes, genus Chiloglanis (Siluriformes: Mochokidae), from Kenya with remarks on other taxa from the area. *Zootaxa*, **4044**(1), 45–64. doi:10.11646/zootaxa.4044.1.2.

Schuster PA and Machado RJP (2021) Unknown diversity: survey of Neuroptera (Insecta) in Paraná, southern Brazil, reveals 14 species newly recorded from the state and country. *Check List*, **17**(3), 993–1005. doi:10.15560/17.3.993.

Skovgaard A, Karpov S and Guillou L (2012) The Parasitic Dinoflagellates Blastodinium spp. Inhabiting the Gut of Marine, Planktonic Copepods: Morphology, Ecology, and Unrecognized Species Diversity. *Frontiers in Microbiology*, **3**. Retrieved from https://www.frontiersin.org/articles/10.3389/fmicb.2012.00305

Slapcinsky J and Lasley R (2007) Three new species of Paryphantopsis (Gastropoda: Pulmonata: Charopidae) from the Nakanai Mountains, New Britain, Papua New Guinea. *The Nautilus*, **121**(4).

Smith DG and Heard RW (2001) A New Species of Chelorchestia (Amphipoda: Talitridae) from Southwest Florida, with Comments on Other Species within the Genus. *Journal of Crustacean Biology*, **21**(4), 1031–1041. doi:10.1163/20021975-99990195.

Solow AR and Smith WK (2005) On estimating the number of species from the discovery record. *Proceedings of the Royal Society B: Biological Sciences*, **272**(1560), 285–287. doi:10.1098/rspb.2004.2955.

Stefan LM, Gómez-Díaz E, Mironov SV, González-Solís J and McCoy KD (2018) “More Than Meets the Eye”: Cryptic Diversity and Contrasting Patterns of Host-Specificity in Feather Mites Inhabiting Seabirds. *Frontiers in Ecology and Evolution*, **6**. Retrieved from https://www.frontiersin.org/articles/10.3389/fevo.2018.00097

Stoev P, Komerički A, Akkari N, Liu S, Zhou X, Weigand A, Hostens J, Hunter C, Edmunds S, Porco D, Zapparoli M, Georgiev T, Mietchen D, Roberts D, Faulwetter S, Smith V and Penev L (2013) *Eupolybothrus cavernicolus* Komerički & Stoev sp. n. (Chilopoda: Lithobiomorpha: Lithobiidae): the first eukaryotic species description combining transcriptomic, DNA barcoding and micro-CT imaging data. *Biodiversity Data Journal*, **1**, e1013. doi:10.3897/BDJ.1.e1013.

Stork NE (2018) How Many Species of Insects and Other Terrestrial Arthropods Are There on Earth? In M. R. Berenbaum, ed., *Annual Review of Entomology, Vol 63*, Vol. 63, Palo Alto: Annual Reviews, , 31–45. doi:10.1146/annurev-ento-020117-043348.

Stork NE, Grimbacher PS, Storey R, Oberprieler RG, Reid C and Slipinski SA (2008) What determines whether a species of insect is described? Evidence from a study of tropical forest beetles. *Insect Conservation and Diversity*, **1**(2), 114–119. doi:10.1111/j.1752-4598.2008.00016.x.

Strong LL, Gilmer DS and Brass JA (1991) Inventory of Wintering Geese with a Multispectral Scanner. *The Journal of Wildlife Management*, **55**(2), 250–259. doi:10.2307/3809147.

Tedesco PA, Bigorne R, Bogan AE, Giam X, Jézéquel C, and Hugueny B (2014) Estimating How Many Undescribed Species Have Gone Extinct. *Conservation Biology*, **28**(5), 1360–1370. doi:10.1111/cobi.12285.

Theng M, Jusoh WFA, Jain A, Huertas B, Tan DJX, Tan HZ, Kristensen NP, Meier R and Chisholm RA (2020) A comprehensive assessment of diversity loss in a well-documented tropical insect fauna: Almost half of Singapore’s butterfly species extirpated in 160 years. *Biological Conservation*, **242**, 108401. doi:10.1016/j.biocon.2019.108401.

Timm T (1998) Lumbriculidae (Oligochaeta) of Lake Taimyr. *Journal of Natural History*, **32**(9), 1291–1301. doi:10.1080/00222939800770641.

Uotila P (1979) Chenopodium exsuccum, a neglected species of the C. foliosum aggregate. *Annales Botanici Fennici*, **16**(3), 237–240.

Valdez JW, Brunbjerg AK, Fløjgaard C, Dalby L, Clausen KK, Pärtel M, Pfeifer N, Hollaus M, Wimmer MH, Ejrnæs R and Moeslund JE (2021) Relationships between macro-fungal dark diversity and habitat parameters using LiDAR. *Fungal Ecology*, **51**, 101054. doi:10.1016/j.funeco.2021.101054.

van der Linde EJ, Pichova K, Pazoutova S, Stodulkova E, Flieger M, Novak P and Kolarik M (2022) Pre-invasion assessment on African invasive grasses revealed five new species of ergot fungi, Claviceps section Pusillae. *Fungal Biology*, **126**(11–12), 752–763. doi:10.1016/j.funbio.2022.09.004.

Vieira C, De Clerck O, Millet L and Payri CE (2019) Description of ten new Lobophora species from the Bismarck Sea (Papua New Guinea). *Phycological Research*, **67**(3), 228–238. doi:10.1111/pre.12372.

Votýpka J, Kment P, Yurchenko V and Lukeš J (2020) Endangered monoxenous trypanosomatid parasites: a lesson from island biogeography. *Biodiversity and Conservation*, **29**(13), 3635–3667. doi:10.1007/s10531-020-02041-2.

Wanninger A (2008) Comparative lophotrochozoan neurogenesis and larval neuroanatomy: Recent advances from previously neglected taxa. *Acta Biologica Hungarica*, **59**, 127–136. doi:10.1556/ABiol.59.2008.Suppl.21.

Webster RP, Hughes C and Sweeney JD (2022) The Coleoptera of the Province of Prince Edward Island, Canada: 295 new records from Lindgren funnel traps and a checklist to species. *ZooKeys*, **1107**, 1–158. doi:10.3897/zookeys.1107.82976.

Wedderburn SD (2018) Multi-species monitoring of rare wetland fishes should account for imperfect detection of sampling devices. *Wetlands Ecology and Management*, **26**(6), 1107–1120. doi:10.1007/s11273-018-9634-7.

Welden AL (1958) Two Unrecognized Species of Cytidia. *Mycologia*, **50**(2), 304–306. doi:10.2307/3756198.

Williams AK and Berkson J (2004) Reducing false absences in survey data: detection probabilities of Red-backed Salamanders. *Journal of Wildlife Management*, **68**(2), 418–428. doi:10.2193/0022-541X(2004)068[0418:RFAISD]2.0.CO;2.

Wilson H, Kendall B and Possingham H (2011) Variability in Population Abundance and the Classification of Extinction Risk. *Conservation Biology : The Journal of the Society for Conservation Biology*, **25**, 747–57. doi:10.1111/j.1523-1739.2011.01671.x.

Wintle BA, Kavanagh RP, McCarthy MA and Burgman MA (2005) Estimating and Dealing with Detectability in Occupancy Surveys for Forest Owls and Arboreal Marsupials. *The Journal of Wildlife Management*, **69**(3), 905–917. doi:10.2193/0022-541X(2005)069[0905:EADWDI]2.0.CO;2.

Wright JJ, Bruce SA, Sinopoli DA, Palumbo JR, and Stewart DJ (2022) Phylogenomic analysis of the bowfin (Amia calva) reveals unrecognized species diversity in a living fossil lineage. *Scientific Reports*, **12**(1), 16514. doi:10.1038/s41598-022-20875-4.

Wyckhuys KAG, Ha N and Fonte SJ (2021) Artefactual depiction of predator-prey trophic linkages in global soils. *Scientific Reports*, **11**(1), 23861. doi:10.1038/s41598-021-03234-7.

Yamaura Y, Andrew Royle J, Kuboi K, Tada T, Ikeno S and Makino S (2011) Modelling community dynamics based on species-level abundance models from detection/nondetection data. *Journal of Applied Ecology*, **48**(1), 67–75. doi:10.1111/j.1365-2664.2010.01922.x.

Zamkovaya T, Foster JS, de Crécy-Lagard V and Conesa A (2021) A network approach to elucidate and prioritize microbial dark matter in microbial communities. *The ISME Journal*, **15**(1), 228–244. doi:10.1038/s41396-020-00777-x.

Table S4: Summary of additional sources supporting Figure 4's cycle of factors perpetuating ignorance of shadow diversity in the conservation discourse.

Section of diagram	Explanatory notes / main points	References
**Cultural and historical context**	• *Encompasses the way in which biodiversity has historically been approached and the traditions and institutional precedents that still hold power to inform the ways in which researching biodiversity is done now*<br>• *Botanical gardens were once centres for driving training of botanists and sending forth "botanical conquistadors" [1].*<br>• *Kew Botanical Gardens is still world-leading in its plant research [2]. Botanical gardens are still the providers of funding and knowledge, as well as specimen storage [3]: the power dynamic is retained to a degree.*	[1] (Bleichmar 2018)<br>[2] (Farjon 2018)<br>[3] (Kew Royal Botanic Gardens 2020)
**Geographical and habitat biases**	• *Numerous studies highlight that we have more biodiversity records and conservation data from the global north, areas closest to the Greenwich Meridian line, and temperate areas, as well as higher data collection and study of species found in wealthier nations [4]*	[4] (Costello et al. 2015; Moussy et al. 2021; Nuñez et al. 2021; Titley et al. 2017)
**Socio-cultural values**	• *Academic communities, such as adhering to particular species concepts and taxonomic norms expected of scientific inquiry [5].*<br>• *Crisis discipline of conservation [6].*<br>• *Species constructs and conservation terms, such as umbrella [7] and keystone species [8].*<br>• *Popular public opinion can rally around one species whilst ignoring or attacking another [9].*<br>• *Some studies conclude public opinion is one of the biggest factors in perpetuating taxonomic bias [10].*	[5] (Meier et al. 2021)<br>[6] (Sarkar 2019; Soulé 1985)<br>[7] (Breckheimer et al. 2014)<br>[8] (Mills et al. 1993)<br>[9] (Crowley et al. 2018; Garibaldi and Turner 2004; Hooykaas et al. 2022; RSPB 2016; Tsing 2015)<br>[10] (Troudet et al. 2017)
**Funding**	• *Vertebrates and megafauna tend to receive the greatest amounts of funding at species-level research [11]*<br>• *NGO conservation fundraising is an example of the result of this focus on mega fauna [12]*<br>• *Exceptions include recent funding allocated for the Society for Protection of Underground Networks [13], and continued calls for greater funding towards megafauna [14].*	[11] (Di Marco et al. 2017; Ford et al. 2017)<br>[12] (Smith et al. 2012)<br>[13] (Society for Protection of Underground Networks (SPUN) 2021)<br>[14] (Ripple et al. 2016)
**Technology**	• *Availability of technology which supports particular types of conservation studies, ranging from the invention and availability of the microscope [15], to DNA sequencing, particularly the High Throughput Sequencing we see now*<br>• *Researchers tend to choose species more practical to study, those that offer accessible sampling and standardised taxonomic approaches [16]. Physical traits, or "biological impediments" make particular species less visible and*	[15] (Ainsworth 1976)<br>[16] (Pawar 2003; Wilson et al. 2007)<br>[17] (Mammola et al. 2021)

	therefore less likely to show in ecological sampling data and more difficult to sample or study [17].	
**Taxonomic bias**	• *In addition to habitat, geographical, and funding mentioned above we also note a well-documented taxonomic bias towards vertebrates (Bonnet et al. 2002; Seddon et al. 2005; Donaldson et al. 2016; Troudet et al. 2017).*<br>• *Lorimer's [19] notion of 'nonhuman charisma' suggests particular species possess characteristics more appealing than others through aesthetic, ecological and corporeal charisma. The result is that more research occurs on vertebrates most closely resembling humans, those who have a high emotional impact when interacted with, and those easy to recognise.*<br>• *Invertebrates, plants and parasites are last to be mentioned in research where they are mentioned at all [20].*<br>• *Popular opinion receives blame over the choices of individual scientists for this taxonomic bias [21].*	[18] (Bonnet et al. 2002; Donaldson et al. 2016; Seddon et al. 2005; Troudet et al. 2017)<br>[19] (Lorimer 2015, 43–60)<br>[20] (Ceballos et al. 2020)<br>[21] (Troudet et al. 2017; Wilson et al. 2007)
**Training**	• *Education and training availability and uptake has contributed to a shortage of taxonomists, particularly for less popular species. This 'taxonomic impediment' includes a lack of trained professionals, amongst other factors, resulting in taxonomic blindness [22].*<br>• *A well-cited example is "plant blindness" [23], which includes lack of experience with plants, failure to notice plants, and failure to recognise their ecological value. Plant blindness has led to a lack of taxonomists with adequate interest or training in botany and plant identification.*<br>• *As Knapp [24] notes, plant blindness could reasonably be extended as blindness to all non-vertebrate species. This is a position echoed by a recent German project to study dark taxa of insects: this required training taxonomists with new specialist skills [25].*<br>• *Whilst Joppa and colleagues [26] debate the relative drop in numbers of taxonomists compared to species, most literature across neglected taxa seems to agree that there are not enough taxonomists trained in species beyond large vertebrates.*	[22] (Engel et al. 2021)<br>[23] (Wandersee and Schussler 1999)<br>[24] (Knapp 2019)<br>[25] (German Barcode of Life (GBOL) 2020)<br>[26] (Joppa et al. 2011)

List of References for Table S4

Ainsworth GC (1976 [2009]) *Introduction to the History of Mycology*, [re-printed edition 2009], Cambridge: Cambridge University Press.

Bleichmar D (2018) Botanical conquistadors. In E. C. Spary, H. A. Curry, J. A. Secord and N. Jardine (eds), *Worlds of Natural History.* Cambridge: Cambridge University Press, 236–254. doi:10.1017/9781108225229.015.

Bonnet X, Shine R and Lourdais O (2002) Taxonomic chauvinism. *Trends in Ecology & Evolution*, **17**(1), 1–3. doi:10.1016/S0169-5347(01)02381-3.

Breckheimer I, Haddad NM, Morris WF, Trainor AM, Fields WR, Jobe RT, Hudgens BR, Moody A and Walters, JR (2014) Defining and Evaluating the Umbrella Species Concept for Conserving and Restoring Landscape Connectivity. *Conservation Biology*, **28**(6), 1584–1593. doi:10.1111/cobi.12362.

Ceballos G, Ehrlich PR and Raven PH (2020) Vertebrates on the brink as indicators of biological annihilation and the sixth mass extinction. *Proceedings of the National Academy of Sciences*, **117**(24), 13596–13602. doi:10.1073/pnas.1922686117.

Costello MJ, Lane M, Wilson S and Houlding B (2015) Factors influencing when species are first named and estimating global species richness. *Global Ecology and Conservation*, **4**, 243–254. doi:10.1016/j.gecco.2015.07.001.

Crowley SL, Hinchliffe S and McDonald RA (2018) Killing squirrels: Exploring motivations and practices of lethal wildlife management. *Environment and Planning E: Nature and Space*, **1**(1–2), 120–143. doi:10.1177/2514848617747831.

Di Marco M, Chapman S, Althor G, Kearney S, Besancon C, Butt N, Maina JM, Possingham HP, Rogalla von Bieberstein K, Venter O and Watson JEM (2017) Changing trends and persisting biases in three decades of conservation science. *Global Ecology and Conservation*, **10**, 32–42. doi:10.1016/j.gecco.2017.01.008.

Donaldson MR, Burnett NJ, Braun DC, Suski CD, Hinch SG, Cooke SJ and Kerr JT (2016) Taxonomic bias and international biodiversity conservation research. *FACETS*, 1, 105-113. doi:10.1139/facets-2016-0011.

Engel MS, Ceríaco LMP, Daniel GM, … Zacharie CK (2021) The taxonomic impediment: a shortage of taxonomists, not the lack of technical approaches. *Zoological Journal of the Linnean Society*, **193**(2), 381–387. doi:10.1093/zoolinnean/zlab072.

Farjon A (2018) The Kew Review: Conifers of the World. *Kew Bulletin*, **73**(1), 8. doi:10.1007/s12225-018-9738-5.

Ford AT, Cooke SJ, Goheen JR and Young TP (2017) Conserving Megafauna or Sacrificing Biodiversity? *BioScience*, **67**(3), 193–196. doi:10.1093/biosci/biw163.

Garibaldi A and Turner N (2004) Cultural Keystone Species: Implications for Ecological Conservation and Restoration. *Ecology and Society*, **9**(3). https://www.jstor.org/stable/26267680.

German Barcode of Life (GBOL) (2020) Dark Taxa – GBOL III: Dark Taxa. https://bolgermany.de/home/gbol3/dark-taxa-2/ (accessed 9 December 2021).

Hooykaas MJD, Holierhoek MG, Westerveld JS, Schilthuizen M and Smeets I (2022) Animal biodiversity and specificity in children's picture books. *Public Understanding of Science*, **31**(5), 671–688. doi:10.1177/09636625221089811.

Joppa LN, Roberts DL and Pimm SL (2011) The population ecology and social behaviour of taxonomists. *Trends in Ecology & Evolution*, **26**(11), 551–553. doi:10.1016/j.tree.2011.07.010.

Kew Royal Botanic Gardens (2020) Africa: Research, curation, safeguarding, identifying and naming of Kew's Herbarium specimens from Africa. https://www.kew.org/science/our-science/departments/accelerated-taxonomy/africa (accessed 3 June 2023).

Knapp S (2019) Are humans really blind to plants? *Plants, People, Planet*, **1**(3), 164–168. doi:10.1002/ppp3.36.

Lorimer J (2015) *Wildlife in the Anthropocene: Conservation after Nature.* Minneapolis: University of Minnesota Press.

Mammola S, Lunghi E, Bilandžija H, Cardoso P, Grimm V, Schmidt S, Hesselberg T and Martínez A (2021) Collecting eco-evolutionary data in the dark: Impediments to subterranean research and how to overcome them. *Ecology and Evolution*, **11**(11), 5911–5926. doi:10.1002/ece3.7556.

Meier R, Blaimer BB, Buenaventura E, Hartop E, von Rintelen T, Srivathsan A and Yeo D (2022) A re-analysis of the data in Sharkey et al.’s (2021) minimalist revision reveals that BINs do not deserve names, but BOLD Systems needs a stronger commitment to open science. *Cladistics*, **38**(2):264-275. doi: 10.1111/cla.12489.

Mills LS, Soulé ME and Doak DF (1993) The Keystone-Species Concept in Ecology and Conservation. *BioScience*, **43**(4), 219–224. doi:10.2307/1312122.

Moussy C, Burfield IJ, Stephenson PJ, Newton AFE, Butchart SHM, Sutherland WJ, Gregory RD, McRae L, Bubb P, Roesler I, Ursino C, Wu Y, Retief EF, Udin JS, Urazaliyev R, Sánchez-Clavijo LM, Lartey E and Donald PF (2021) A quantitative global review of species population monitoring. *Conservation Biology*, 1–14. doi:10.1111/cobi.13721.

Nuñez MA, Chiuffo MC, Pauchard A and Zenni RD (2021) Making ecology really global. *Trends in Ecology & Evolution*, **36**(9), 766–769. doi:10.1016/j.tree.2021.06.004.

Pawar S (2003) Taxonomic Chauvinism and the Methodologically Challenged. *BioScience*, **53**(9), 861–864. doi:10.1641/0006-3568(2003)053[0861:TCATMC]2.0.CO;2.

Ripple WJ, Chapron G, López-Bao JV, Durant SM, Macdonald DW, Lindsey PA, Bennett EL, Beschta RL, Bruskotter JT, Campos-Arceiz A, Corlett RT, Darimont CT, Dickman AJ, Dirzo R, Dublin HT, Estes JA, Everatt KT, Galetti M, Goswami VR, Hayward MW, Hedges S, Hoffmann M, Hunter LTB, Kerley GIH, Letnic M, Levi T, Maisels F, Morrison JC, Nelson MP, Newsome TM, Painter L, Pringle RM, Sandom CJ, Terborgh J, Treves A, Van Valkenburgh B, Vucetich JA, Wirsing AJ, Wallach AD, Wolf C, Woodroffe R, Young H and Zhang L (2016) Saving the World’s Terrestrial Megafauna. *BioScience*, **66**(10), 807–812. doi:10.1093/biosci/biw092.

RSPB (2016) Hedgehog Wins Favourite UK Mammal Poll. https://www.rsb.org.uk/news/www.rsb.org.uk//news/hedgehog-wins-favourite-uk-mammal-poll (accessed 3 June 2022).

Sarkar S (2019) What Should “Biodiversity” Be? In E. Casetta, J. Marques da Silva, and D. Vecchi, eds., *From Assessing to Conserving Biodiversity: Conceptual and Practical Challenges*, Cham: Springer International Publishing, 375–399. doi:10.1007/978-3-030-10991-2_18.

Seddon PJ, Soorae PS and Launay F (2005) Taxonomic bias in reintroduction projects. *Animal Conservation*, **8**(1), 51–58. doi:https://doi.org/10.1017/S1367943004001799.

Smith RJ, Veríssimo D, Isaac NJB and Jones KE (2012) Identifying Cinderella species: uncovering mammals with conservation flagship appeal. *Conservation Letters*, **5**(3), 205–212. doi:10.1111/j.1755-263X.2012.00229.x.

Society for Protection of Underground Networks (SPUN) (2021) Press. https://spun.earth/press/ (accessed 9 December 2021).

Soulé ME (1985) What Is Conservation Biology? *BioScience*, **35**(11), 727–734. doi:10.2307/1310054.

Titley MA, Snaddon JL and Turner EC (2017) Scientific research on animal biodiversity is systematically biased towards vertebrates and temperate regions. *PLoS ONE*, **12**(12), e0189577. doi:10.1371/journal.pone.0189577.

Troudet J, Grandcolas P, Blin A, Vignes-Lebbe R and Legendre F (2017) Taxonomic bias in biodiversity data and societal preferences. *Scientific Reports*, **7**(1), 9132. doi:10.1038/s41598-017-09084-6.

Tsing AL (2015) *The mushroom at the end of the world: on the possibility of life in capitalist ruins*, Princeton, New Jersey: Princeton University Press.

Wandersee JH and Schussler EE (1999) Preventing Plant Blindness. *The American Biology Teacher*, **61**(2), 82–86. doi:10.2307/4450624.

Wilson JR, Procheş Ş, Braschler B, Dixon ES and Richardson DM (2007) The (bio)diversity of science reflects the interests of society. *Frontiers in Ecology and the Environment*, **5**(8), 409–414. doi:https://doi.org/10.1890/060077.1.
