# Supplementary material for: The diversity of ignorance and the ignorance of diversity: origins and implications of “shadow diversity” for conservation biology and extinction
Source: Camb Prism Extinct. 2024 Nov 22;2:e18. doi: 10.1017/ext.2024.21 (PMC11895729; doi:10.1017/ext.2024.21)
Supplement: Turton-Hughes et al. supplementary material 7 — Turton-Hughes et al. supplementary material [file S2755095824000214sup007.docx]

**Table 1: Exploring categories of ignorance from Gross (2007) in relation to shadow diversity sub-categories**

| Term | Definition | Interpretation for Shadow Diversity | Example |
| --- | --- | --- | --- |
| Ignored | ***Negative knowledge*** “Knowledge about what is not known, but considered as unimportant or even dangerous” (Gross 2007, 751) | *This transposes neatly onto scenarios and species that are neglected in biodiversity sampling or studies: species known to science, yet not considered sufficiently valuable or significant for measurement or study. It refers to a level of intentional choice (see Proctor 2008).* | Distribution of urban rabbits or rats. This category is in danger of unnoticed extinctions, as in the case of Berger (2008). |
| Obscured | Missing from Gross’s definitions and process of ignorance, we compare this sub-category to ***unknown knowns.*** | *For the purposes of biodiversity, this relates to species for which there are scientific records, but where practicalities prevent further knowledge or make this difficult to achieve. Examples of this would collected species in herbariums and museums, yet to be described and lacking taxonomic expertise; holding a comprehensive list of species of a certain kingdom on record, but no taxonomic expertise available to survey a region being studied (both considered forms of “taxonomic impediment”); or habitats in extreme locations where species inhabiting the area may already be known to science, but cannot be surveyed accurately due to physical inaccessibility of a site, also referred to as “habitat impediment”(Mammola et al. 2021).* | Missing data for bryophytes, orchids, asters, and begonias (Bánki et al. 2022; Cornwell et al. 2019; Ratnasingham and Hebert 2007); herbarium and museum specimens, yet to be described and lacking taxonomic expertise. |
| Undiscovered | ***Non-knowledge*** “Knowledge about what is not known but taking it into  account for future planning” (Gross 2007, 751) | *This speaks to the work of predictions for undiscovered biodiversity at global (Moura and Jetz 2021) and national scales (such as Lu et al., 2021). The scientific community knows undiscovered species remain and has estimated numbers and locations for this, although they have yet to be found. This shares similarities with Kerwin’s (1993) known unknowns but goes further in the inclusion in planning and prioritising future research. This action-oriented aspect is particularly relevant for the field of conservation which is action-oriented and calls for urgent work.* | Cone Snails (Chivian *et al. 20*03); mycorrhizal fungi (Society for Protection of Underground Networks (SPUN) 2021); likelihood of new fungal species (Banchi et al. 2018; Cheek et al. 2020); suspected additional cryptic species amongst insects thought to be generalists (Sheikh et al. 2022) and lichens (Lücking et al. 2014); habitat type: inaccessible tropical location (Scheffers et al. 2012); deep sea (Jamieson et al. 2021). |
| Unimagined | ***Nescience*** “Lack of any knowledge: prerequisite for a total surprise beyond any type of anticipation – can lead to ignorance and non-knowledge, but belongs to a different epistemic class from the above terms.” (Gross 2007, 751) | *Reserved for extreme unknown-unknown forms of biodiversity, beyond our current capabilities of awareness and only accessible in retrospect.* | Awareness during (or prior to) the 1970s of the *Chromista* taxonomic kingdom, established in 1981 (Cavalier-Smith 2018). |
