# Supplementary material for: The diversity of ignorance and the ignorance of diversity: origins and implications of “shadow diversity” for conservation biology and extinction
Source: Camb Prism Extinct. 2024 Nov 22;2:e18. doi: 10.1017/ext.2024.21 (PMC11895729; doi:10.1017/ext.2024.21)
Supplement: Peer review history

# Editor report: Recommendation: The diversity of ignorance and the ignorance of diversity: origins and implications of “shadow diversity” for conservation biology and extinction — R0/PR2

**DOI:** 10.1017/ext.2024.21.pr2

**Affiliations:** Center for the Advancement of the Steady State Economy, United States

The first two reviewers made diametrically opposed judgments about the suitability of this manuscript for publication, namely “Accept” vs. “Reject”, with the third one tipping the balance toward acceptance. Together, the three offer many important and substantive critiques and suggestions. When you re-submit the article, please describe, in a separate document, the changes you have made in response to the reviews, and in case you disagree with any parts of them, spell out why.

# Editor report: Decision: The diversity of ignorance and the ignorance of diversity: origins and implications of “shadow diversity” for conservation biology and extinction — R0/PR3

**DOI:** 10.1017/ext.2024.21.pr3

**Affiliations:** School of Natural Sciences, University of Tasmania, Australia

No accompanying comment.

# Editor report: Recommendation: The diversity of ignorance and the ignorance of diversity: origins and implications of “shadow diversity” for conservation biology and extinction — R1/PR5

**DOI:** 10.1017/ext.2024.21.pr5

**Affiliations:** Center for the Advancement of the Steady State Economy, United States

Given the revision of the article in response to the comments of all three reviewers, and the change of recommendation to “accept” by the one reviewer who originally said “reject”, it seems fine to publish this.

# Editor report: Decision: The diversity of ignorance and the ignorance of diversity: origins and implications of “shadow diversity” for conservation biology and extinction — R1/PR6

**DOI:** 10.1017/ext.2024.21.pr6

**Affiliations:** School of Natural Sciences, University of Tasmania, Australia

No accompanying comment.
